# Microbial diversity and mineral composition of weathered serpentine rock of the Khalilovsky massif

**DOI:** 10.1371/journal.pone.0225929

**Published:** 2019-12-12

**Authors:** Irina V. Khilyas, Alyona V. Sorokina, Anna A. Elistratova, Maria I. Markelova, Maria N. Siniagina, Margarita R. Sharipova, Tatyana A. Shcherbakova, Megan E. D’Errico, Michael F. Cohen

**Affiliations:** 1 Institute of Fundamental Medicine and Biology, Kazan (Volga Region) Federal University, Kazan, Russian Federation; 2 FSUE Central Research Institute of Geology of Non-metallic Mineral Resources, Kazan, Russian Federation; 3 School of Science and Technology, Sonoma State University, Rohnert Park, CA, United States of America; 4 Department of Biology, Sonoma State University, Rohnert Park, CA, United States of America; University of Utah, UNITED STATES

## Abstract

Endolithic microbial communities survive nutrient and energy deficient conditions while contributing to the weathering of their mineral substrate. This study examined the mineral composition and microbial communities of fully serpentinized weathered rock from 0.1 to 6.5 m depth at a site within the Khalilovsky massif, Orenburg Region, Southern Ural Mountains, Russia. The mineral composition includes a major content of serpentinite family (mostly consisting of lizardite and chrysotile), magnesium hydrocarbonates (hydromagnesite with lesser amounts of hydrotalcite and pyroaurite) concentrated in the upper layers, and clay minerals. We found that the deep-seated weathered serpentinites are chrysotile-type minerals, while the middle and surface serpentinites mostly consist of lizardite and chrysotile types. Microbial community analysis, based on 16S rRNA gene sequencing, showed a similar diversity of phyla throughout the depth profile. The dominant bacterial phyla were the *Actinobacteria* (of which unclassified genera in the orders *Acidimicrobiales* and *Actinomycetales* were most numerous), *Chloroflexi* (dominated by an uncultured P2-11E order) and the *Proteobacteria* (predominantly class *Betaproteobacteria*). Densities of several groups of bacteria were negatively correlated with depth. Occurrence of the orders *Actinomycetales*, *Gaiellales*, *Solirubrobacterales*, *Rhizobiales* and *Burkholderiales* were positively correlated with depth. Our findings show that endolithic microbial communities of the Khalilovsky massif have similar diversity to those of serpentine soils and rocks, but are substantially different from those of the aqueous environments of actively serpentinizing systems.

## Introduction

Microbial communities colonizing mineral surfaces are of significant evolutionary and ecological interest. Mineral surfaces are extreme econiches, having microbiota affected by a large number of exogenous factors such as temperature, light, salinity, high pH, deficiency and unavailability of nutrients [1]. The mineral surface microtopography, structure, chemistry and reactivity are selective factors for microbial colonization, formation of biofilms and ultimately for the whole microbial community structure. Mineral-associated microorganisms often contribute to geochemical processes including mineral formation, dissolution or deterioration [2, 3].

One enigmatic mineral considered as a potential locus for the origin of life on Earth is serpentinite [4]. Serpentinite comprising of the antigorite, lizardite, and chrysotile group minerals is an ultramafic rock mineral that occurs in all ancient orogenic belts [5] within the oceanic lithosphere at surfaces and faults[6, 7]. Formation of serpentinite occurs at relatively low temperatures through hydration of olivine-rich ultramafic rocks to serpentine, hydrogen, methane, low-molecular organic compounds [8] and, under conditions of sufficient SiO_2_, magnetite (Fe_3_O_4_) [9].

In natural environments, terrestrial serpentinites (Mg,Fe,Ni,Mn,Zn)_2-3_(Si,Al,Fe)_2_O_5_(OH)_4_ undergo a weathering process accompanied by consistent physical and chemical changes of minerals through dissolution, decomposition and carbonation. Hydrolysis of serpentinites is accompanied by release of Mg^2+^, HCO^3 –^and SiO_2_, which is followed by formation of magnesium hydrocarbonates and amorphous silica oxide [10]. Serpentinite weathering is associated with increasing of alkalinity of deep-seated minerals within the geologic formation [11, 12]. We hypothesized that such changes in serpentine rocks impact the resident microbiota, which in turn could have a role in the development of serpentinite-derived infertile soils.

The Khalilovsky massif is located near the town of Novorudnyy, Gaysky District, Orenburg Region, Southern Ural Mountains, Russia (S1A and S1B Fig). The approximately 260 km^2^ massif was deposited in the middle Paleozoic era bordered by the Uraltausky anticlinorium and greenstone synclinorium [13]. The main rock types within the massif are serpentinized lizardite and chrysotile (95–98%) with lesser amounts of clay minerals, chlorite, dolomite, calcite and magnesite[13]. The serpentine associated with the ophiolite complexes in this region are derived from harzburgite and dunite, reflecting a more melt depleted oceanic mantle[14]. Presently, the massif is characterized by rolling hills covered in some places by 1–5 cm layer of soil with grass and streams running through the deposit (S1C Fig). One of the most significant waterways of the region is the Guberlya River. The Southern Ural Mountains have an arid continental climate. The average temperature for the coldest (January) and the warmest (July) months of the year is –20°C and +40°C, respectively. The deposit receives an average of 181 mm of precipitation per year with a mean annual evaporation rate of 988 mm.

The purposes of this study were to (1) characterize the microbial community diversity of the fully serpentinized weathered Khalilovsky massif subsurface samples and compare them with those reported for other serpentinite-hosted systems, and (2) to perform geochemical and mineralogical analyses and determine the role, if any, of the resident microbial community in the weathering process. The results presented here show that bacterial diversity, as indicated by 16S rRNA gene sequence analysis, did not vary significantly at the phyla level with depth within our sampling site, although differences in the dominant microbial orders and genera were found. We also demonstrate that the microbial community does not appear to substantially alter its serpentine mineral substrate.

## Materials and methods

### Site characteristics and sample collection

Using a dry drilling rig (URB-2A2, Spezburmash, Russia), a single 112 mm-diameter, 6.85 m deep core was taken from an exposed section of weathered serpentine rock, without soil overlay, located in the central part of the Khalilovsky massif (Latitude: N 51°30’ Longitude: E 58° 07’) in August 2017. Two duplicate samples (55.0–60.0 g) taken from opposite sides of the core were collected at the depths specified in Table 1 into sterile plastic boxes. Each sample was crushed under sterile laboratory conditions using a mortar and pestle. The mineral particle size for analysis was approximately 0.01 mm. The pH of water extracted samples ranged between pH 8.98 to pH 9.06. For the collection of serpentine from the Khalilovky massif a specific permission is not required. The Khalilovsky massif is not related to a national park or other protected areas of land of the Russian Federation.

**Table 1:** Sample X-Ray diffraction mineral identification and percent composition in the serpentine rock core taken at the Khalilovsky massif^a^.

Sample depth (m)	Serpentinite (%)	Hydrotalcite-type and pyroaurite group minerals (%)	Hydromagnesite (%)	Clay minerals (%)	Calcite (%)
**0.1**	72.3 ±2.5	10.8 ±1	15.1 ±1	1.8 ±0.5	-
**0.85**	82.1 ±2.0	6.7 ±0.7	9.3 ±0.8	2.0 ±0.5	-
**1.6**	79.0 ±2.1	9.6 ±0.8	9.2 ±0.8	2.2 ±0.5	-
**2.35**	72.1 ±2.5	12.2 ±1	15.5 ±1	1.2 ±0.5	-
**3.1**	84.0 ±1.6	7.8 ±0.8	8.2 ±0.8	-	-
**3.85**	81.4 ±2.1	11.3 ±1	4.9 ±0.6	2.5 ±0.5	-
**4.6**	81.6 ±2.2	9.8±1	7.3 ±0.7	1.3 ±0.5	-
**5.35**	87.5 ±2.0	6.6 ±0.7	3.7 ±0.6	1.5 ±0.5	0.8±0.2
**6.1**	89.0 ±1.7	5.6 ±0.6	4.0 ±0.6	1.4 ±0.5	-
**6.85**	93.5 ±1.1	4.1 ±0.6	-	2.3 ±0.5	-

^a^Values are the mean ± range of duplicate samples of the core taken at a given depth.

### X-ray diffraction (XRD)

Analyses of the mineralogical samples were performed on powdered samples using a D8 Advance XRD2 Bruker diffractometer, with CuK-alpha radiation, at 40 kV, 30 mA. X-ray diffractograms were collected within the 2θ range (5–40°) with 0.05/1 sec step. Mineralogical composition was determined with advance diffract plus evaluation software (DIFFRACplusTOPAS Software package).

### Scanning electron microscopy (SEM)

The mineralogical samples were coated with a conductive layer of Au/Pd alloy at an 80/20 ratio using a cathode sputtering method in the Quorum Q150T ES vacuum chamber. The thickness of the conductive layer was 15 nm. Measurements were conducted using a high-resolution field-emission scanning electron microscope Merlin (Carl Zeiss). Imaging of the surface morphology was carried out with 5 kV acceleration voltage of the primary electrons and 300 pA probe current for minimal impact on the object of research.

Elemental analysis was conducted using an energy dispersive spectrometer (EDS) AZtec X-Max (resolution 127 eV) under 20 kV acceleration voltages of the electron probe and 10 mm working distance in order to avoid minimal errors in the microprobe analysis. The probing depth was approximately 1 μm.

### Transmission electron microscopy (TEM)

Analysis of samples was carried out using a Hitachi HT7700 Exalens transmission electron microscope. To prepare the sample 5 μl of the mineralogical suspension was placed on a Formvar/carbon 3 mm coated copper grid and dried at room temperature. After drying, the grid was transferred into the transmission electron microscope. Analysis was conducted at an accelerating voltage of 100kV in TEM mode.

### DNA extraction, 16S rRNA gene amplification and sequencing

To extract DNA 10 g of crushed rock from each depth sample was submersed in 5 ˗ 10 mL DNAse-RNase-free water for one week at room temperature. The mineral suspension was subjected to three liquid N_2_ freeze/thaw cycles[15], lysozyme/Proteinase K treatment, followed by a phenol-chloroform extraction step. DNA washing and precipitation was performed using 80% cold ethanol. DNA was resuspended in the DNAse-RNase-free water[15]. The quantity of the extracted DNA was measured using a Qubit 2.0 Fluorometer (Invitrogen, Carlsbad, CA). Bacterial 16S rRNA gene amplicons (∼500-bp fragments) were produced using the 337F (5’-CCTACGGGNGGCWGCAG-3’) and 805R (5’*-*GACTACHVGGGTATCTAATCC-3’) primers[16]. Archaeal 16S rRNA gene fragments were amplified with the forward primer 344F (5'-ACGGGGYGCAGCAGGCGCGA-3’) and the reverse primer 806R (5'- GGACTACVSGGGTATCTAAT-3’)[17]. Reaction products were purified using Ampure® XP paramagnetic beads (Beckman Coulter Inc., USA). The purified PCR products were amplified with Illumina primers containing the unique barcodes. The pooled multiplex reactions were loaded on an Illumina MiSeq instrument with the 2x300 v3 reagent mix.

### Analysis of the specific functional genes in the serpentinite-associated microbial communities

The *mcrA* gene encoding methyl‐coenzyme M reductase (MCR) and *hydA* gene encoding hydrogenase were used to investigate the potential methanogenic activity and hydrogen oxidation in the mineralogical samples using specific primers mcrA-F (5’-GGTGGTGTMGGATTCACACARTAYGCWACAGC-3’) and mcrA-R (5’-TTCATTGCRTAGTTWGGRTAGTT-3’), and FeFe27F (5’-GCHGAYMTBACHATWATGGARGA-3’) and FeFe27R (5’-GCNGCYTCCATDACDCCDCCNGT-3’), respectively[18, 19].

### Bioinformatics analysis

Sequencing of 16S rRNA gene V3-V4 variable regions was performed on the Illumina MiSeq platform in 2x300bp mode. Reads were further processed and analyzed using the QIIME software, version 1.9.1 (http://qiime.org/) [20] according to protocols. Before filtering, there were 66287 read pairs per sample on average. Paired-end reads were initially merged and then processed to remove low quality and chimeric sequence data. The rarefaction step was performed to reduce sequencing depth heterogeneity between samples. After quality filtering, chimera filtering and rarefying, we analyzed 24442 joined read pairs. Sequences were clustered into operational taxonomic units (OTU) based on the 97% identity threshold (open reference-based OTU picking strategy), the latest SILVA database v.132, 13-12-17 [21] was used. Each OTU was required to contain at least 2 sequences for inclusion in the final OTU list. The number of observed OTUs varied from 1702 to 3578. To characterize the richness and evenness of the bacterial community, alpha diversity indices were calculated using Chao1, Shannon and Simpson metrics (S2 Table). Similarities between microbial compositions of samples were evaluated using the beta diversity characteristics, which were estimated using weighted and unweighted Unifrac measures with further non-metric multidimensional scaling (nMDS) visualization.

Raw reads are deposited in the ENA under Project ID PRJEB29885 in the fastq format (https://www.ebi.ac.uk/ena/data/view/PRJEB29885).

## Results and discussion

### Mineral morphology and geochemical composition

Rocks containing the serpentine group of minerals are present in all orogenic belts worldwide, typically forming large massifs along continental margins and mélange, faults and shear zones. The geochemical, mineralogical and microbiological features of serpentinites attract much interest among researchers in the field of geology, mineralogy, geochemistry and microbiology. The process of weathering results in serpentinites of different mineral maturities. Mineral rocks of the Khalilovsky massif are represented by massive and coarsely fractured lizardite and chrysotile with small amounts of bastite [13]. We observed two distinct serpentine zones of the massif: (1) the soil-grass serpentine ecosystem; and (2) the solid rocky nutrient poor serpentine ecosystem from which we obtained our core (S1 Fig).

We collected duplicate samples at given depths from 0.1 m to 6.85 m from a single core drilled at a site presently undergoing erosion and weathering (Table 1). This weathered serpentinite has a distinct greenish grey color and fine to medium-grained texture, which is distinct from rocky serpentinites that are characterized by a dark greenish grey color and dense homogenization (S2 Fig). The mineralogical composition of the rock core samples was evaluated by X-ray diffraction and found to be represented by serpentinite minerals and hydromagnesite, with a minority of hydrotalcite, pyroaurite, clay minerals and calcite (Table 1, S3 Fig). Magnesium hydrocarbonate minerals (hydrotalcite-type and pyroaurite group minerals and hydromagnesite) are known to accrete in veins and fissures formed through surface weathering[22]. Consistent with this, the content of these minerals decreased in depth from 0.1 m to 6.85 m and, conversely, the proportion of serpentinite group minerals increased in the deep-seated layers. Calcium carbonates (e.g. calcite) were negligible or completely absent throughout.

**Fig 1:**
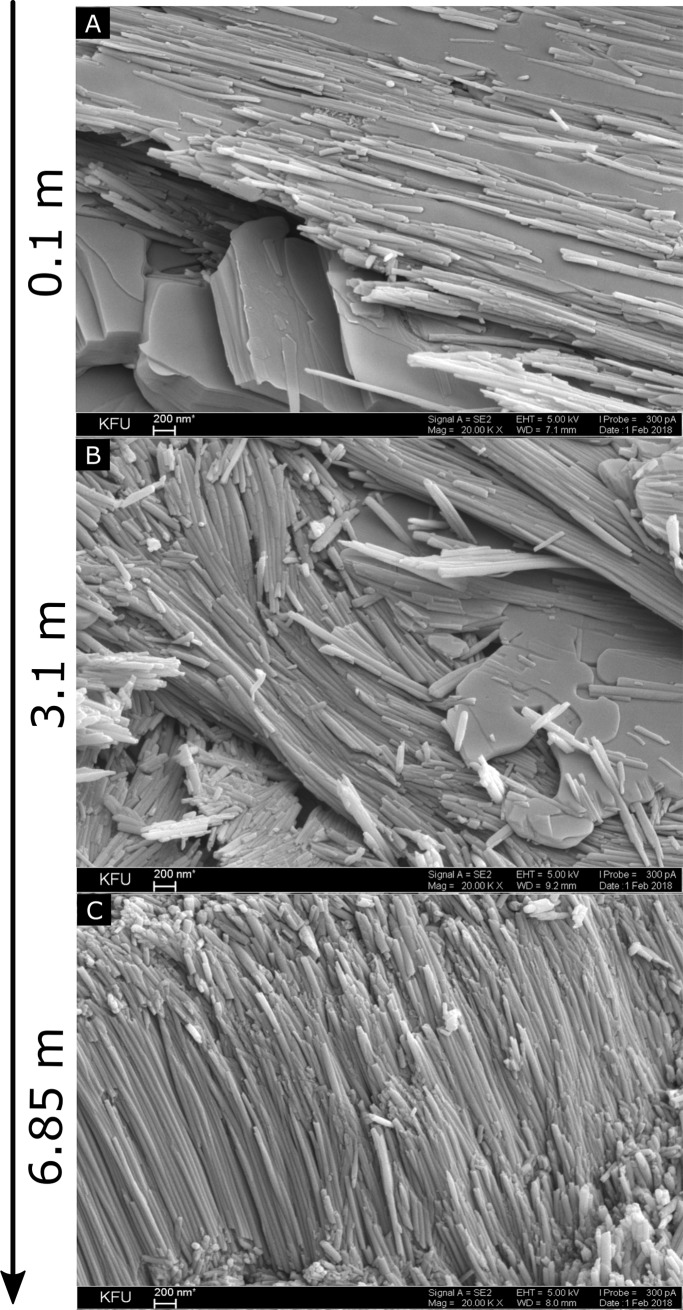
SEM of Khalilovsky massif serpentine rock core samples collected at (A) 0.1 m, (B) 3.1 m and (C) 6.85 m in depth.

Serpentine rocks include a range of major minerals, such as lizardite, chrysotile, antigorite and other minorities characterized by different texture and structure[12]. Scanning electron microscopy (SEM) and energy dispersive X-ray spectrometry (EDS) was used to characterize the surface morphology and determine the chemical phases of serpentinites collected from different depths. A plate-like morphology, characteristic of lizardite[23], with a minor inclusion of cylindrical fibers was observed in serpentinite collected from 0.1 m depth (Fig 1A). Plates and unoriented fibers, often arranged in parallel strands, were characteristic of the serpentinite collected from the 3.1 m depth (Fig 1B). The deep-seated serpentinites (6.85 m in depth) displayed well oriented, needle-shaped hollow fibers, or nanotubes, that are characteristic of chrysotile [23] (Fig 1C). The sizes of the particles observed in this study ranged from less than 1 μm to greater than 200 μm. As determined by EDS, oxygen, magnesium, silicon and iron were the predominant elements in the mineralogical samples (S4 Fig). Additionally, the transition metals nickel and chromium were detected in the upper layers of the serpentine deposit, only nickel was found in the middle layers, but both elements were absent in the deep-seated serpentinites (S4 Fig). Transmission electron microscopy (TEM) was applied to investigate the crystal structure of serpentinites. The irregular shaped plates and hollow cylinders with different thicknesses were typical for all serpentinites (S5 Fig).

### Microbial community composition

**Fig 2:**
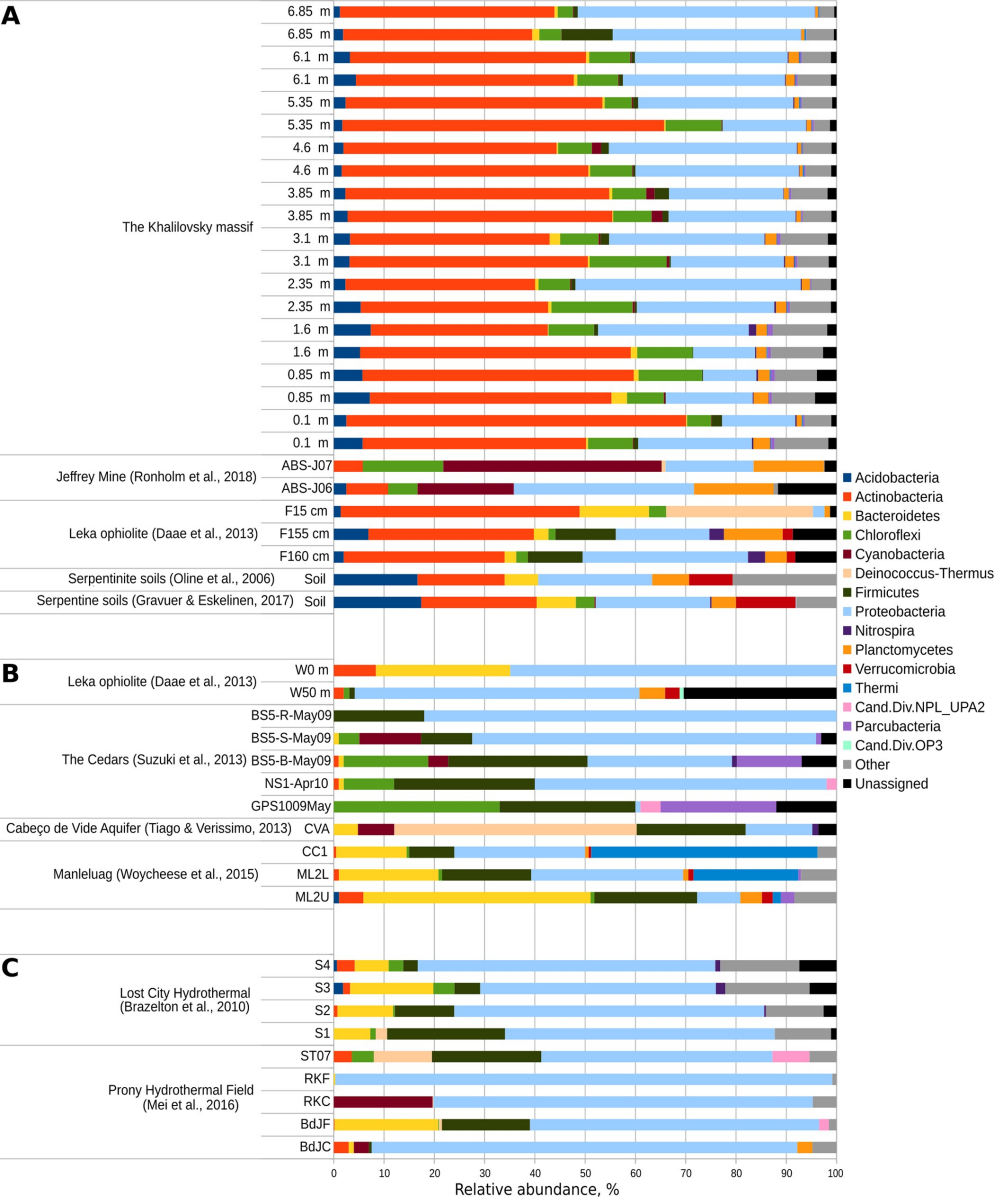
Comparative analysis of mineral- and aqueous-associated serpentine-hosted bacterial communities according to Phylum. (**A**) Community profiles of the Khalilovsky massif compared to those associated with serpentine minerals in other terrestrial environments. (**B**, **C**) Profiles of selected aqueous-associated planktonic communities from (**B**) terrestrial environments and (**C**) seawater serpentine- and ultramafic-hosted systems [24–32].

We characterized the microbial diversity of the weathered serpentine minerals sampled from the core obtained from a solid rocky zone of the Khalilovsky massif. The composition of microbial communities was similar throughout the 0.1 m to 6.85 m depth profile at the phylum/class level. Among the 31 different phyla found, *Actinobacteria*, *Proteobacteria* and *Chloroflexi* were the most abundant phyla in all of the mineralogical samples (Fig 2).

**Fig 3:**
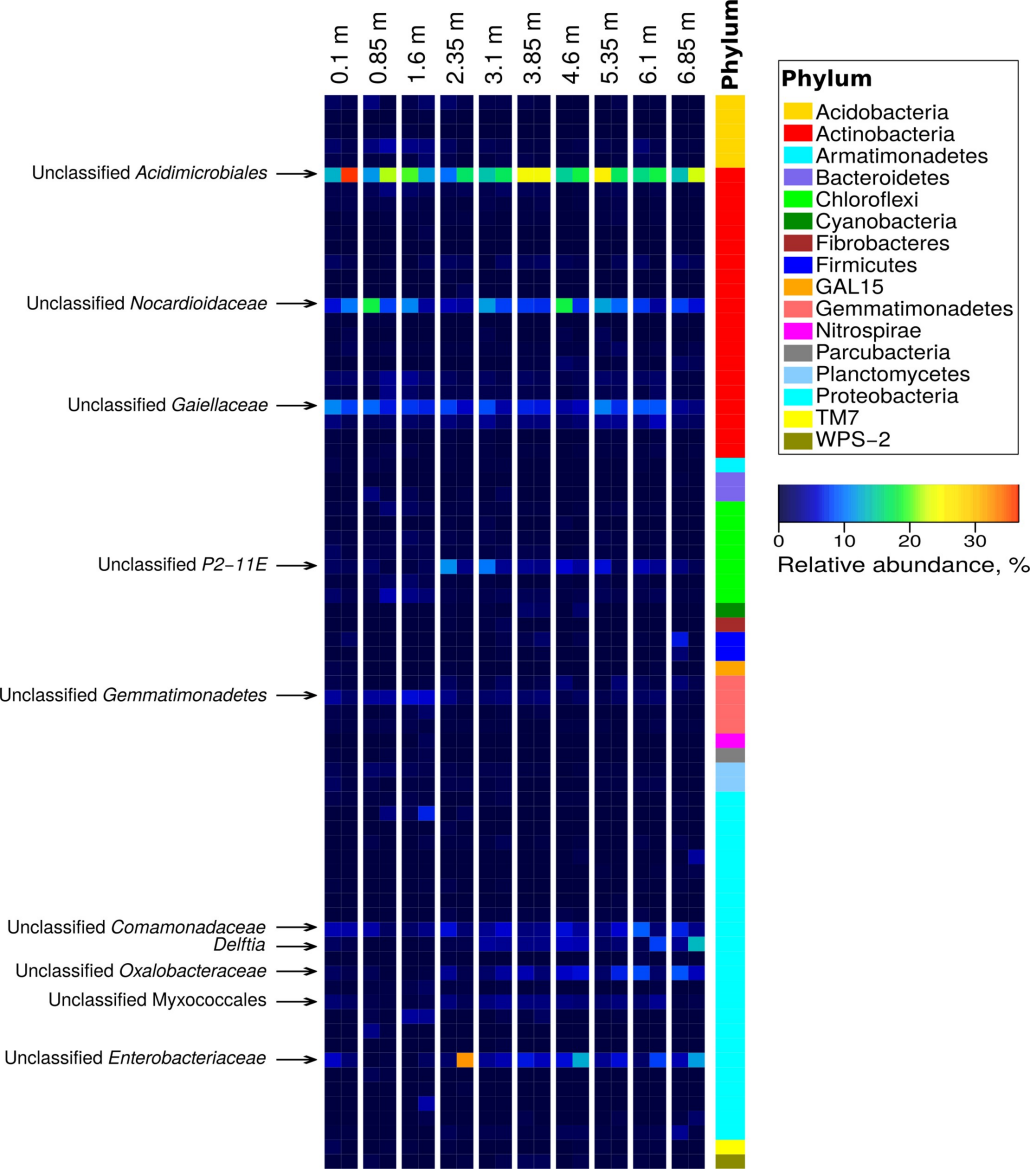
A heat map of the bacterial genera found in serpentine rock core samples collected at different depths. Colors indicate percent relative abundance of 74 genus level taxonomic groupings (i.e. 97% OTU sequence identity) that occur within the major phyla (each representing ≥0.5% of all OTUs) detected at different depths. Names of the most highly represented genera are given.

The dominant phylum *Actinobacteria* throughout the core depths was represented primarily by an unclassified genus within the order *Acidimicrobiales*, comprising 11.0% to 38.0% of all OTU sequences, and members of the order *Actinomycetales* (*Nocardioidaceae* family), comprising 9.9% to 21.5% of OTUs (Fig 3, S3 Table). The phenotypic basis for the success of these particular groups within this habitat remains to be determined. The phylum *Proteobacteria* was represented mainly by the classes *Betaproteobacteria* (*Comamonadaceae*, *Oxalobacteraceae* and *Delftia*), *Gammaproteobacteria* (*Enterobacteriaceae* family, *Serratia* genus), and *Deltaproteobacteria* (*Myxococcales* order, *Haliangiaceae* family). Members of the genus *Delftia* are known to be distributed in the microbial communities of a deep subsurface thermal aquifer system (Siberia, Russia), a deep subsurface crystalline rock the Pyhäsalmi mine (Finland), and ornamental limestone from buildings [33–35]. Among the phylum *Chloroflexi* there was an abundance of an uncultured P2-11E order of bacteria.

### Microbial community composition correlating with depth

**Fig 4:**
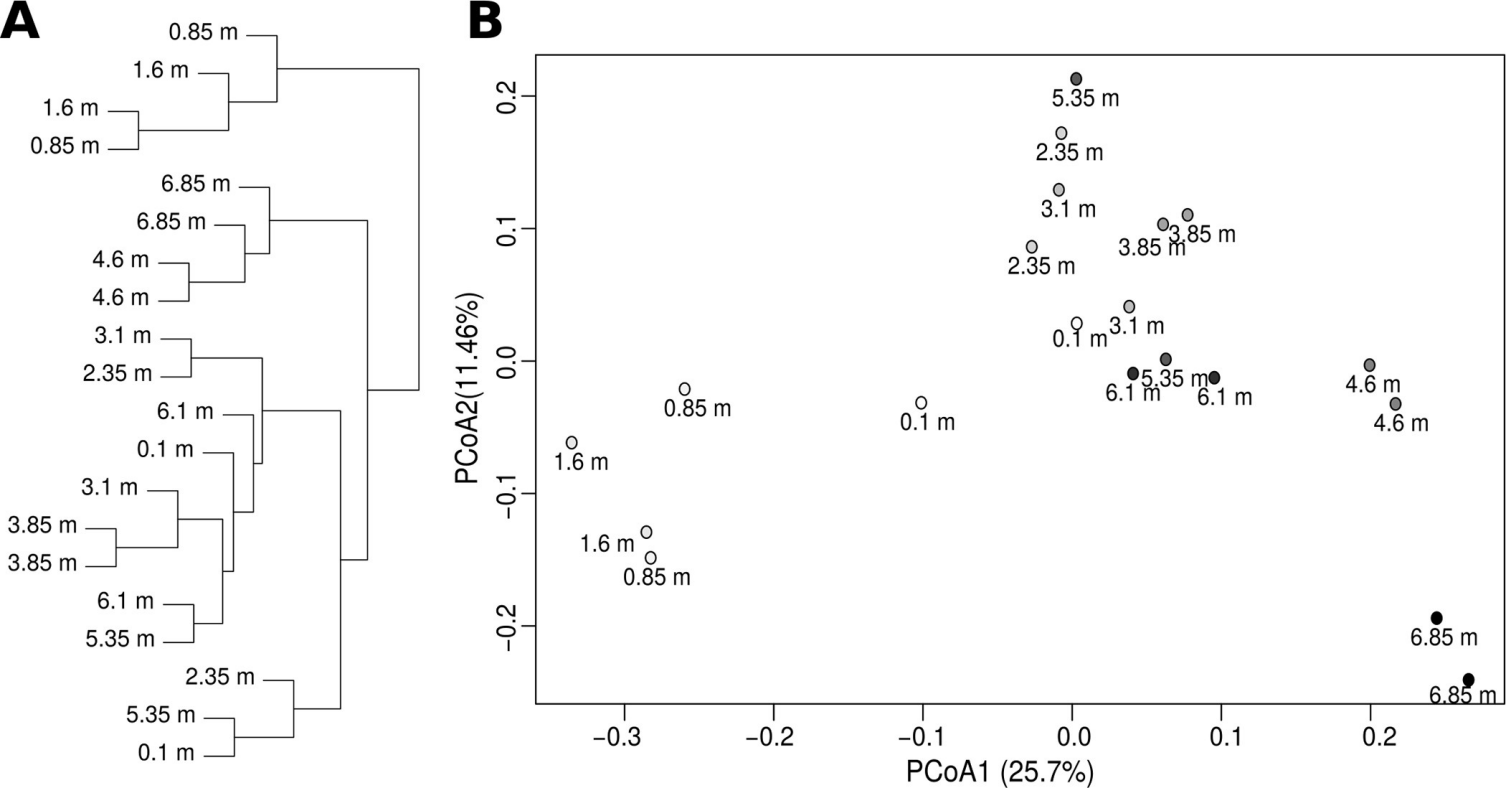
Clustering analyses performed based on Bray-Curtis dissimilarity distances of prokaryotic OTU sequence abundance. (A) A hierarchical cluster tree; and (B) principal coordinate analysis (PCoA) scatter plot. PCoA1 and PCoA2 explained 25.7% and 11.46% of the observed variation. Black and grey-colored circles indicate the depth of the microbial communities.

Non-metric Bray-Curtis dissimilarity analysis (at a 97% similarity level) showed that microbial communities of the serpentine core samples collected from the different depths were closely related (Fig 4). Similar results were obtained using PCoA analysis, which explained 37.16% of the observed variation (Fig 4B).

Mineral porosity and water circulation are expected to be important factors influencing the microbial community of the Khalilovsky massif. Weathering-induced cracking at shallower depths as well as the fibrous tubular-type serpentinite nanostructures contribute to rock porosity [12] that should in turn positively impact serpentine-associated microflora since microbial colonization can only occur within fissures of rocks. Statistically significant correlations of the OTU abundances from the rock core relative to their distribution according to depth are presented in Table S4 Table. The orders *Acidimicrobiales*, *Gaiellales*, *Solirubrobacterales*, *Gemmatimonadetes*, *Rhizobiales*, *Burkholderiales*, P2-11E and unidentified members related to the GAL15 phylum positively correlated with depth. Additionally, several orders show a negative correlation with depth. Precipitation of hydromagnesite from magnesium-rich alkaline water percolating through fissures can occur without microbial mediation [36]but extracellular polymeric substances of adherent biofilms can accelerate such precipitation[37]. Whether the microbes residing in the serpentine rock fissures have contributed to the hydromagnesite deposition cannot by ascertained from our results.

### Comparison to other serpentine-related microbial communities

To date, investigations have largely focused on microbial communities hosted in aqueous actively serpentinizing systems, enriched hydrogen and methane[24, 26–32]. However, relatively limited publications have reported on microbial communities associated with fully serpentinized rocks. Our results indicate that the microbial community of the mature serpentine Khalilovsky massif are more similar to serpentine mineral surface-associated (Fig 2A) than aqueous phase-associated microbial communities (Fig 2B & 2C).

These findings are consistent with those of Daae et al. [25]who found the groundwater microbial communities to be substantially different from the fracture-coating microbial communities within partially serpentinized dunite from the Leka ophiolite, Norway (Fig 2A & 2B). The *Actinobacteria* phylum, similar to our findings, predominated in the mineral fractions taken from 15 and 160 cm in depth, whereas in the fluid passing through minerals were dominated by members of *Betaproteobacteria*.

Some progress has been made in characterizing microbial communities associated with serpentinite-derived soils. A study of bacterial community structure by 16S rRNA gene clone library analysis found the dominant bacterial phyla in serpentine soil to be *Actinobacteria*, *Acidobacteria*, *Alphaproteobacteria*, *Verrucomicrobia*, Green-nonsulfur-related bacteria and *Gemmatimonadetes*[28], whose members are known to be well adapted to low moisture soils. We found *Gemmatimonadetes* consistently at frequencies ranging up to 6% in all of our mineral samples from the Khalilovsky massif (S3 Table).

Endolithic microbial communities of the heavily serpentinized open-pit chrysotile asbestos Jeffrey mine (Québec, Canada) were dominated by *Proteobacteria*, *Cyanobacteria* and *Planctomycetes*[29].

Analysis of ultramafic serpentine soils including harsh and lush serpentines of the McLaughlin Reserve in the Northern Coast Ranges of California revealed that *Proteobacteria* and *Actinobacteria* were two abundant phyla in the microbial communities of minerals taken from a depth of 7.5 cm[26]. Among *Actinobacteria*, the orders *Acidimicrobiales* and *Gaiellales* were relatively abundant.

*Firmicutes*, a ubiquitous bacterial phylum inhabiting a wide range of ecosystems, is more highly represented in actively serpentinizing systems. Within the deep-seated weathered serpentinite of the arid Khalilovsky massif, the capacity to survive desiccation conferred by endospores may be responsible for the status of *Bacillus* as the dominant genus for this phylum (S3 Table). Acceleration of serpentinite dissolution by a *Bacillus* species under laboratory conditions was postulated to occur through the action of secreted organic acids and ligands[38]. Thus, *Bacillus* as well as other bacteria that secrete such compounds could have a role in initiating and widening fissures within serpentine rock, leading to soil formation.

Similar to our findings at the Khalilovsky massif, Archaea were not found on the rocks from the Jeffery mine [29] and are proportionally less represented on mineral surface communities within other serpentinized systems [25, 26] relative to aqueous-associated communities of actively serpentinizing fields[8, 24, 31, 32, 39–41]. Consistent with the lack of Archaea, we did not detect the methyl coenzyme-M reductase (*mcrA*) gene, which is a common indicator of methane production within actively serpentinizing environments [8]. Similarly, we were not able to amplify from our samples the *hydA* gene, a marker of bacterial hydrogen metabolism in actively serpentinizing environments[8, 27, 42]. Furthermore, the H_2_-oxidizing genus *Hydrogenophaga* did not exceed 0.26% of the sequences in any given core sample (S3 Table), whereas they reached up to 58% of the total bacterial community in samples collected from a chimney of the PHF[27].

## Conclusions

The results of this study demonstrate the texture, microstructure, geochemical composition and microbial diversity within nutrient-poor, fully serpentinized weathered rock found at the Khalilovsky massif, Russia. SEM analysis revealed plate and fibrous tubular structures characteristic of lizardite and chrysotile, respectively, of the serpentinites collected from different depths. Geochemical analysis showed increasing serpentinite-type mineral content and decreasing content of magnesium hydrocarbonates with depth, indicative of weathering-induced fissuring closer to the surface. 16S rRNA phylogenetic analysis of the endolithic microbial communities demonstrated a similar distribution of phyla throughout the 0.1 m to 6.85 m sampled depth, with *Actinobacteria*, and *Proteobacteria* being the predominant phyla followed by *Chloroflexi* and *Acidobacteria*. The basis for the dominance among the *Actinobacteria* of a single unclassified genus of *Acidimicrobiales* deserves further study. The Khalilovsky massif rock core microbial communities were substantially different from aqueous serpentine environments and relatively close to those of fully serpentinized rock surfaces and soils, notably in regard to the predominance of *Actinobactera*.

## Supporting information

S1 Fig The Khalilovsky massif location and sample collection site. (A) The Gaysky District, Orenburg Region, Southern Ural Mountains, Russia (B) The Khalilovsky massif (C) The serpentinite rolling hills.
(PDF)

S2 Fig Photographs of serpentinite minerals before (A) and after crushing (B).
(PDF)

S3 Fig **Representative X-ray diffraction (XRD) patterns of rock core samples collected at (A) 0.1 m, (B) 3.1 m and (С) 6.85 m in a depth.** HM–Hydromagnesite, Sp–Serpentinites, HT–Hydrotalcite, Py–Pyroaurite.
(PDF)

S4 Fig Backscattered electron images (BSE) with elemental spectra of the serpentinites collected at (A) 0.1 m, (B) 3.1 m and (C) 6.85 m in depth.
(PDF)

S5 Fig Transmission electron micrographs (TEM) of serpentinites collected at (A) 0.1 m, (B) 3.1 m and (C) 6.85 m in a depth.
(PDF)

S6 Fig Rarefaction curve of OTUs (operational taxonomic units) for 20 rock core samples. (PDF)

S1 Table The geochemical composition of serpentinites at the Khalilovsky massif. % = С х 10^n^, C–content of elements, n–degree.
(PDF)

S2 Table The diversity indices correlated with species richness (Shannon's index), species diversity (Simpson's index) and evenness and species richness estimators (Chao1). (PDF)

S3 Table Taxonomic assignments for all 16S rRNA tag sequences from rock core samples of the Khalilovsky massif, Russia. (PDF)

S4 Table Spearman’s rank correlation coefficients between relative abundance of OTUs and depth of serpentine minerals. (PDF)
