# Supplementary material for: Microbial diversity and mineral composition of weathered serpentine rock of the Khalilovsky massif
Source: PLoS One. 2019 Dec 12;14(12):e0225929. doi: 10.1371/journal.pone.0225929 (PMC6907791; doi:10.1371/journal.pone.0225929)
Supplement: S1 Fig — (A) The Gaysky District, Orenburg Region, Southern Ural Mountains, Russia (B) The Khalilovsky massif (C) The serpentinite rolling hills. (PDF) [file pone.0225929.s001.pdf]

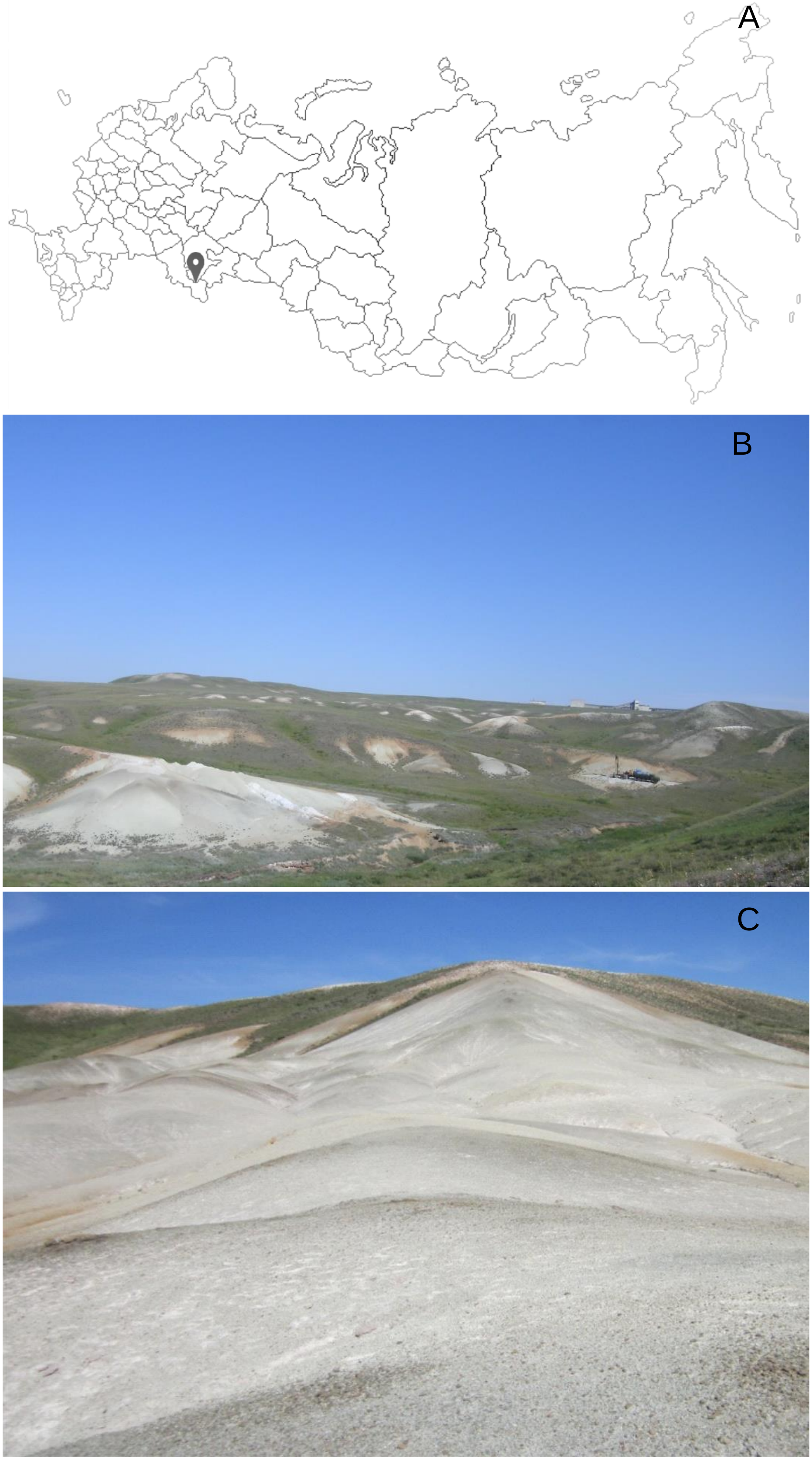

S1 Fig. The Khalilovsky massif location and sample collection. (A) The Gaysky District, Orenburg Region, Southern Ural Mountains, Russia (B) The Khalilovsky massif (C) The serpentinite rolling hills.
