# Supplementary figures and images for: Microbial diversity and mineral composition of weathered serpentine rock of the Khalilovsky massif
Source: PLoS One. 2019 Dec 12;14(12):e0225929. doi: 10.1371/journal.pone.0225929 (PMC6907791; doi:10.1371/journal.pone.0225929)

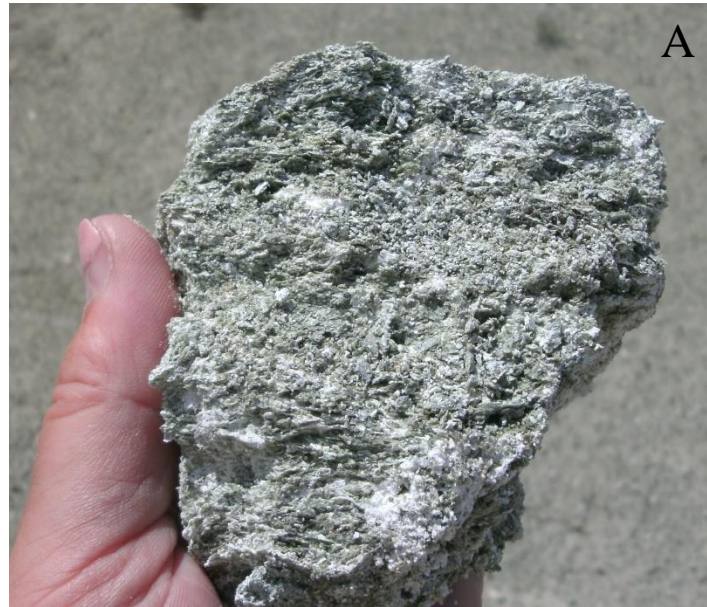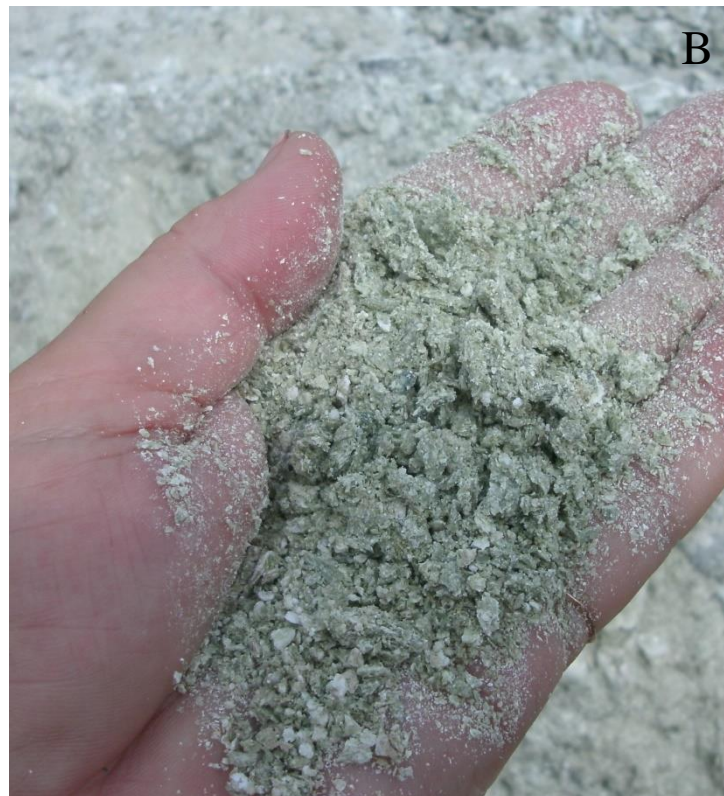

**S2 Fig. Photographs of serpentinite mineral before (A) and after crushing (B).**

Supplement: S2 Fig — Photographs of serpentinite minerals before (A) and after crushing (B). (PDF) [file pone.0225929.s002.pdf]

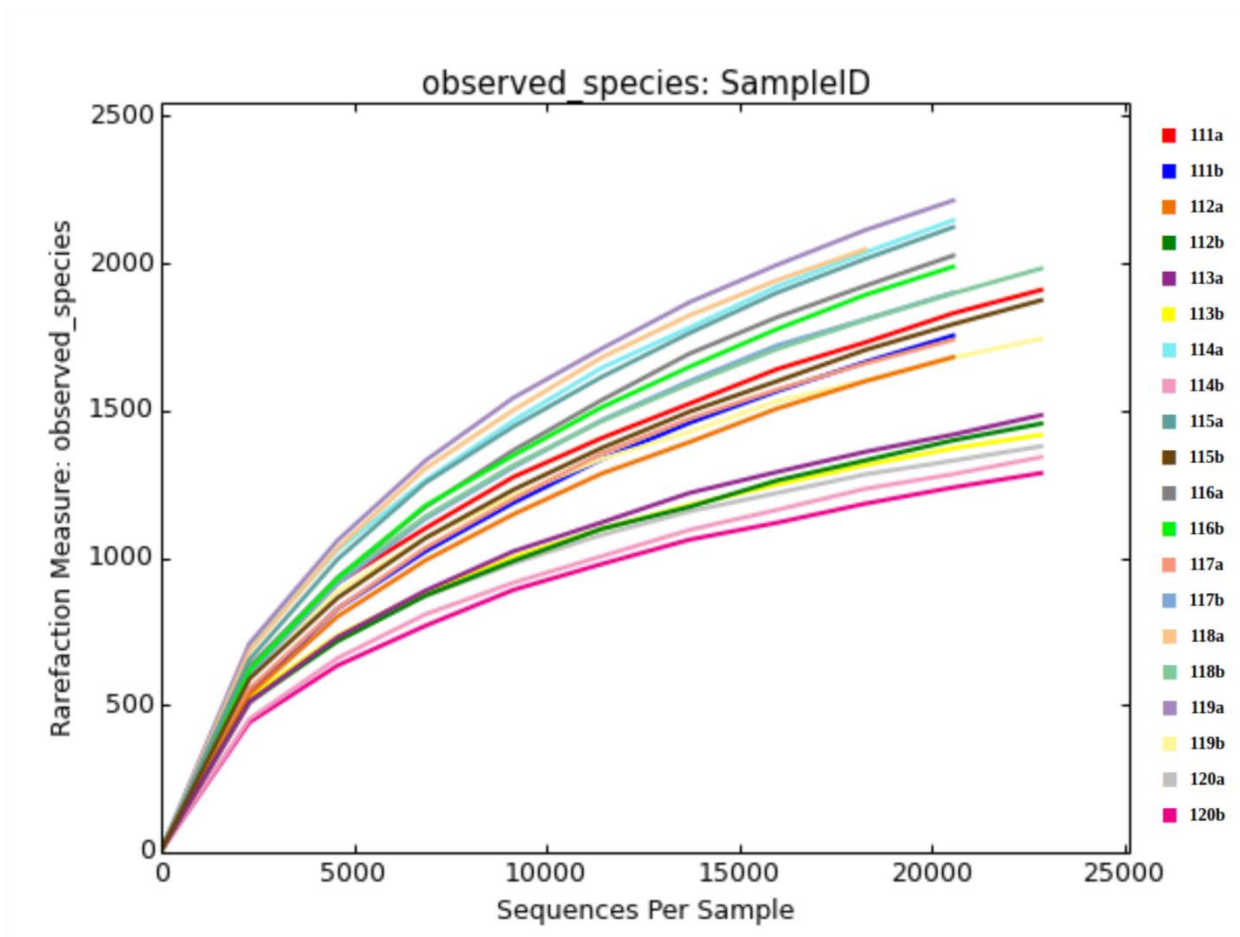

**S6 Fig. Rarefaction curve of OTU (operational taxonomic unit) for 20 serpentinite samples.**

Supplement: S6 Fig — (PDF) [file pone.0225929.s006.pdf]
