# Supplementary material for: Microbial diversity and mineral composition of weathered serpentine rock of the Khalilovsky massif
Source: PLoS One. 2019 Dec 12;14(12):e0225929. doi: 10.1371/journal.pone.0225929 (PMC6907791; doi:10.1371/journal.pone.0225929)
Supplement: S3 Fig — Representative X-ray diffraction (XRD) patterns of rock core samples collected at (A) 0.1 m, (B) 3.1 m and (С) 6.85 m in a depth. HM–Hydromagnesite, Sp–Serpentinites, HT–Hydrotalcite, Py–Pyroaurite. (PDF) [file pone.0225929.s003.pdf]

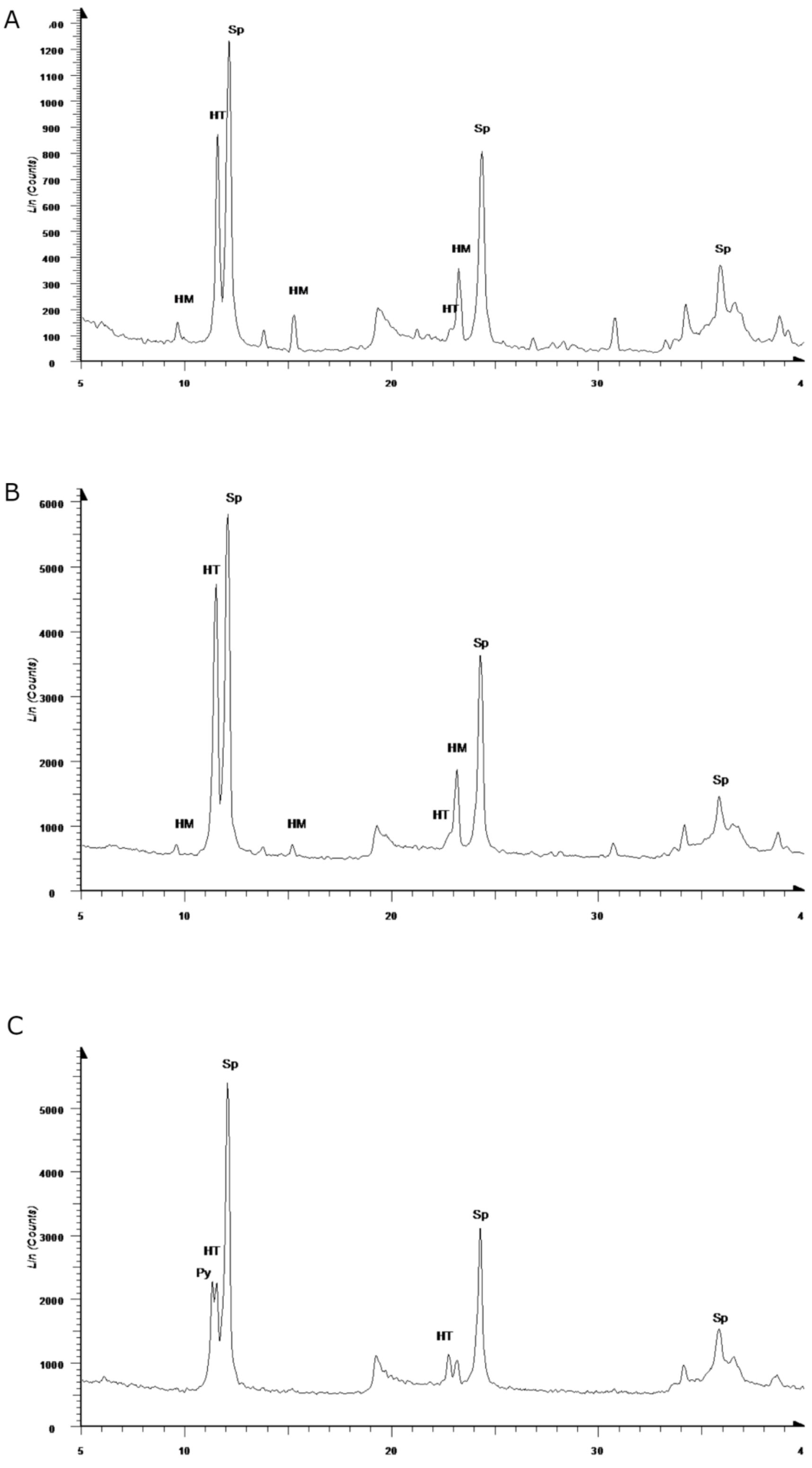

S3 Fig. A representative the X-ray diffraction (XRD) pattern of the serpentinites collected at (A) 0.1 m, (B) 3.1 m and (C) 6.85 m in a depth. HM – Hydromagnesite, Sp – Serpentinites, HT – Hydrotalcite Py – Pyroaurite.
