# Supplementary material for: Microbial diversity and mineral composition of weathered serpentine rock of the Khalilovsky massif
Source: PLoS One. 2019 Dec 12;14(12):e0225929. doi: 10.1371/journal.pone.0225929 (PMC6907791; doi:10.1371/journal.pone.0225929)
Supplement: S5 Fig — Transmission electron micrographs (TEM) of serpentinites collected at (A) 0.1 m, (B) 3.1 m and (C) 6.85 m in a depth. (PDF) [file pone.0225929.s005.pdf]

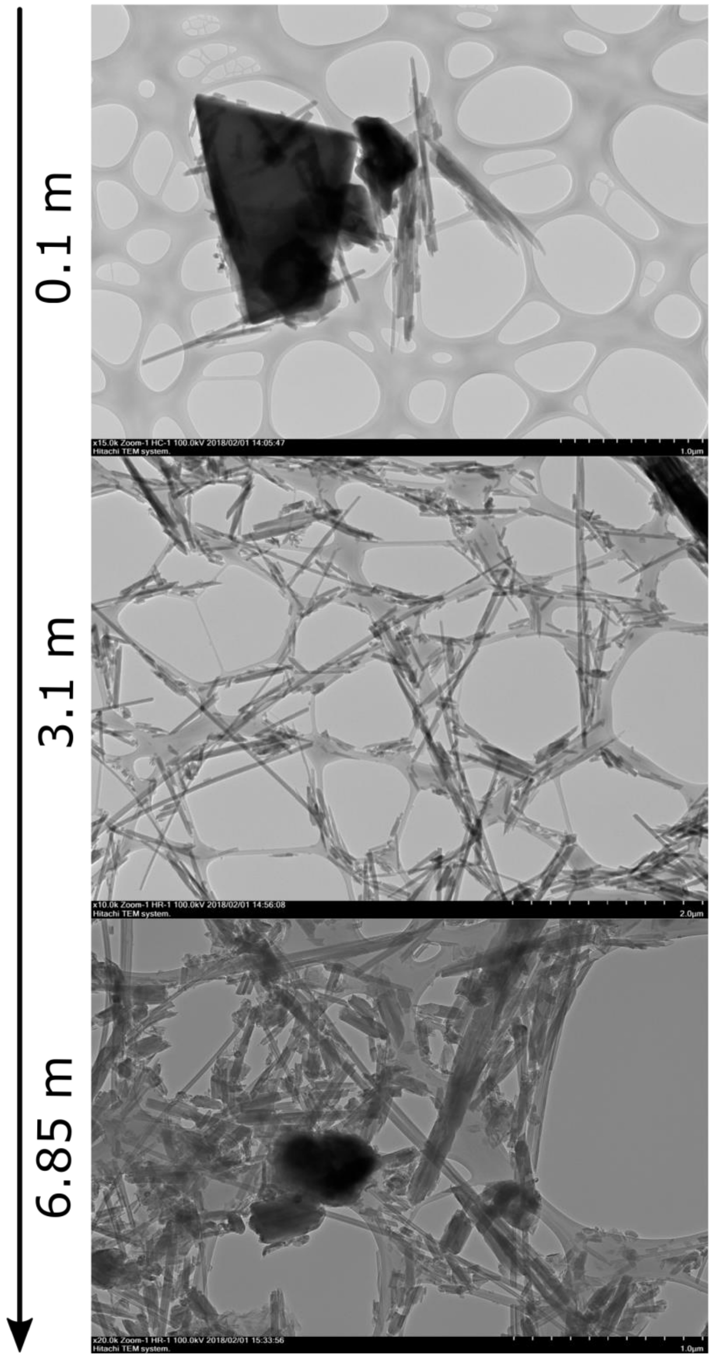

S4 Fig. Transmission electron micrographs (TEM) of serpentinites collected at (A) 0.1 m, (B) 3.1 m and (C) 6.85 m in a depth.
