# Supplementary material for: Microbial diversity and mineral composition of weathered serpentine rock of the Khalilovsky massif
Source: PLoS One. 2019 Dec 12;14(12):e0225929. doi: 10.1371/journal.pone.0225929 (PMC6907791; doi:10.1371/journal.pone.0225929)
Supplement: S1 Table — % = С х 10n, C–content of elements, n–degree. (PDF) [file pone.0225929.s007.pdf]

S1 Table. Geochemical composition of serpentinites at the Khalilovsky massif. % = C x 10^n, C – content of elements, n – degree

n	Elements	111 (0.1 m)	112 (0.85 m)	113 (1.6 m)	114 (2.35 m)	115 (3.1 m)	116 (3.85 m)	117 (4.6 m)	118 (5.35 m)	119 (6.1 m)	120 (6.85 m)
-3	La	<0,2	<0,2	<0,2	<0,2	<0,2	<0,2	<0,2	<0,2	<0,2	<0,2
-2	Ba	0,5	0,3	0,3	0,3	0,3	0,3	0,3	0,5	0,3	0,3
-4	Be	<0,8	<0,8	<0,8	<0,8	<0,8	<0,8	<0,8	<0,8	<0,8	<0,8
-2	As	0,02	0,04	0,04	0,04	0,04	0,02	0,04	0,04	0,02	0,04
0	Ca	<0,02	<0,02	<0,02	<0,02	<0,02	0,5	0,5	0,8	<0,02	<0,02
0	Fe	4,5	5	5	5	5	5,5	4,8	5,5	5,5	6
-3	Co	8,5	7,5	8,5	8,5	10	7,5	7,5	7,5	10	7,5
-3	B	0,8	0,5	0,3	0,5	0,8	0,8	0,5	0,8	1,2	1,5
-3	Sc	0,5	0,5	0,3	0,5	0,5	0,5	0,5	0,3	0,5	0,8
-1	P	0,5	0,3	0,5	0,3	0,3	0,1	0,5	0,8	0,5	0,5
-3	Pb	0,1	0,1	0,1	0,1	0,1	0,1	0,1	0,1	0,1	0,1
-4	Sn	3,5	4,5	3	2	4,5	4,5	3	3,5	2,5	2,5
0	Mg	>10	>10	>10	>10	>10	>10	>10	>10	>10	>10
-3	V	0,1	0,1	0,1	0,1	0,1	0,1	0,1	0,5	0,1	0,1
-3	Ga	0,1	0,1	0,1	0,1	0,1	0,1	0,1	0,1	0,1	0,1
-2	Mn	7,5	7,5	6,5	7,5	7,5	7,5	8,5	7,5	6,8	5,8
-1	Ti	0,003	0,01	0,001	0,003	0,003	0,01	0,003	0,01	0,003	0,003
-3	Nb	0,1	0,1	0,1	0,1	0,1	0,1	0,1	0,1	0,1	0,3
-3	Cr	200	200	180	180	280	280	150	250	220	200
-4	Ge	1	1	1	1	1	1	1	1	1	1
0	Al	0,1	0,5	0,3	0,3	0,1	1,8	1,2	1,5	0,5	0,5
-3	Ni	120	110	120	100	120	100	80	120	150	120
-4	Mo	0,8	0,8	0,8	0,8	0,8	0,8	1,2	0,8	0,8	1,2
-3	Li	2	2	2	2	2	2	2	2	2	2
-2	Zr	0,02	0,04	0,04	0,02	0,02	0,04	0,04	0,08	0,04	0,02
-3	Cu	0,3	0,8	0,8	0,3	0,5	0,5	0,5	0,5	0,8	0,3
-4	Yb	1,8	2	1,5	1,8	1,5	1,8	1,2	1,2	1,5	1,5
-1	Na	0,1	0,1	0,1	0,1	0,1	0,1	0,1	0,3	0,3	0,1
-3	Y	0,3	0,5	0,3	0,3	0,5	0,3	0,3	0,7	0,8	0,5
-2	Zn	0,8	0,9	0,9	0,5	0,8	0,8	0,7	0,8	0,8	0,7
-2	Sr	0,8	0,8	0,8	0,8	0,8	0,8	0,8	0,8	0,8	0,8
-1	K	2	2	4	2	4	2	2	2	2	2
0	Si	15	18	18	15	18	15	18	18	20	20
