# Supplementary material for: Microbial diversity and mineral composition of weathered serpentine rock of the Khalilovsky massif
Source: PLoS One. 2019 Dec 12;14(12):e0225929. doi: 10.1371/journal.pone.0225929 (PMC6907791; doi:10.1371/journal.pone.0225929)
Supplement: S2 Table — (PDF) [file pone.0225929.s008.pdf]

S2 Table. The diversity indices correlated with species richness (Shannon's index), species diversity (Simpson's index) and evenness and species richness estimators (Chao1).

The depth of the sample collection, m	PD_whole_tree	Chao1	Observed_OTUs	Shannon	Simpson
0.1	62,08688	4542,0275	2537	8,74806952	0,99233576
	53,66034	4724,78261	2582	7,61043988	0,95877737
0.85	51,14977	4470	2523	7,99310976	0,97571675
	55,05366	3053,88153	1829	8,05760036	0,98844645
1.6	54,07964	2983,4552	1812	7,91512241	0,9840352
	59,53867	2586,30233	1702	8,14882488	0,98845314
2.35	64,65808	6447,52918	3280	9,02118771	0,98858521
	62,54557	3212,41111	1822	6,73133172	0,92973233
3.1	61,53807	5972,81261	3265	8,63542227	0,98416634
	61,79264	4801,00247	2556	8,28082917	0,98549583
3.85	55,03816	6016,4008	3071	8,30574531	0,98065736
	60,03839	6011,71743	3056	8,34414934	0,98046968
4.6	55,20996	4815,00637	2703	7,87501357	0,97441021
	60,11378	5293,00192	2953	8,27721118	0,98132172
5.31	54,12265	5918,23565	3437	8,5284713	0,97336187
	68,98591	5446,47945	2835	8,61617893	0,98823103
6.1	58,85417	6211,49706	3578	9,15183085	0,99205485
	63,91576	3803,61308	2303	8,31619296	0,98457407
6.85	68,37193	2811,16038	1868	7,88913245	0,98482496
	64,54274	2689,56184	1705	6,88104779	0,95716962
