# Supplementary material for: Microbial diversity and mineral composition of weathered serpentine rock of the Khalilovsky massif
Source: PLoS One. 2019 Dec 12;14(12):e0225929. doi: 10.1371/journal.pone.0225929 (PMC6907791; doi:10.1371/journal.pone.0225929)
Supplement: S3 Table — (PDF) [file pone.0225929.s009.pdf]

S3 Table. Taxonomic assignments for all 16S rRNA tag sequences from rock core samples of the Khalilovsky massif, Russia.

#OTU ID	0.1m		0.85m		1.6m		2.35m		3.1m		3.85m		4.6m		5.31m		6.1m		6.85m	
Unassigned;Other;Other;Other;Other;Other;Other	1,650865	1,055922	4,293381	3,908457	2,689847	1,862664	1,137779	1,132231	1,57685	1,741925	1,033458	1,799747	1,071188	1,003834	1,316266	0,875759	1,131262	1,126527	0,538465	0,470122
k__Archaea;p__Crenarchaeota;c__Thaumarchaeota;o__Nitrososphaerales;f__Nitrososphaeraceae;g__Candidatus Nitrososphaera;s__SCA1145	0	0	0	0	0	0	0	0,004132	0,004332	0	0	0	0	0,004308	0	0,00419	0	0	0	0
k__Bacteria;p__;c__;o__;f__;g__;s__	0	0	0	0	0	0	0,004294	0	0	0,004228	0	0	0	0	0	0	0,004402	0	0	0
k__Bacteria;p__Acidobacteria;c__Acidobacteria-6;o__iii1-15;f__;g__;s__	1,808882	0,815171	2,853934	0,527496	0,938986	1,879112	2,112404	1,008264	0,810085	0,909014	0,736339	0,705534	0,328947	0,262806	0,262348	0,997276	0,369751	0,612963	0,262971	0,169079
k__Bacteria;p__Acidobacteria;c__Acidobacteria-6;o__iii1-15;f__mb2424;g__;s__	0,532269	0,325224	0,32866	0,116298	0,21322	0,509868	0,545275	0,169421	0,255588	0,283274	0,146407	0,130967	0,156039	0,116324	0,085942	0,18018	0,158465	0,265065	0,183662	0,14846
k__Bacteria;p__Acidobacteria;c__Acidobacteriia;o__Acidobacteriales;f__Acidobacteriaceae;g__;s__	0	0	0	0	0	0	0	0,008264	0	0	0	0	0	0	0	0	0	0	0	0
k__Bacteria;p__Acidobacteria;c__DA052;o__Ellin6513;f__;g__;s__	0	0	0	0	0	0	0	0,008264	0	0	0	0	0	0	0	0,00419	0	0	0	0
k__Bacteria;p__Acidobacteria;c__Solibacteres;o__Solibacterales;f__;g__;s__	0,045742	0,08025	0,079045	0,095531	0,127112	0,037007	0,068696	0,057851	0,051984	0,029596	0,043061	0,021124	0,04639	0,017233	0,01357	0,062854	0,110045	0,070408	0,100179	0,082478
k__Bacteria;p__Acidobacteria;c__[Chloracidobacteria];o__RB41;f__;g__;s__	0,544744	0,11404	0,507551	0,411198	0,405937	0,349507	0,17174	0,070248	0,186276	0,109927	0,159325	0,126743	0,067476	0,142174	0,149267	0,079614	0,224492	0,32719	0,229578	0,065982
k__Bacteria;p__Acidobacteria;c__[Chloracidobacteria];o__RB41;f__Ellin6075;g__;s__	1,971058	0,810948	2,766568	4,107825	2,993275	2,705592	1,635825	0,690083	1,299601	0,98089	0,706196	0,654837	0,442814	0,831502	0,850371	0,653677	2,324148	1,014703	0,500897	0,428884
k__Bacteria;p__Acidobacteria;c__iii1-8;o__DS-18;f__;g__;s__	0,852462	0,371684	0,65732	0,448579	0,553551	1,899671	0,84582	0,330579	0,550165	0,93861	0,977479	0,675961	0,577767	0,555771	0,357337	0,393882	1,232503	0,927728	0,609425	0,35053
k__Bacteria;p__Actinobacteria;Other;Other;Other;Other;Other	0	0,016895	0,00416	0	0,0041	0,004112	0,004294	0	0,008664	0,004228	0,008612	0,016899	0,004217	0,012925	0,01357	0,00419	0,004402	0,016567	0	0,004124
k__Bacteria;p__Actinobacteria;c__Acidimicrobiia;o__Acidimicrobiales;Other;Other;Other	0	0,008447	0,00416	0	0,0041	0	0,012881	0,004132	0,008664	0	0,004306	0,021124	0,016869	0,004308	0,031663	0,020951	0,022009	0,004142	0	0,004124
k__Bacteria;p__Actinobacteria;c__Acidimicrobiia;o__Acidimicrobiales;f__;g__;s__	13,02395	36,59824	11,58214	21,6855	19,83352	11,90789	9,746254	16,73967	13,92306	16,86115	24,50157	24,42755	14,70142	17,94839	25,61969	17,11712	15,52073	17,94989	13,83312	22,61949
k__Bacteria;p__Actinobacteria;c__Acidimicrobiia;o__Acidimicrobiales;f__AKIW874;g__;s__	0,004158	0,025342	0,008321	0,012461	0,012301	0	0,021468	0,012397	0,004332	0	0,004306	0,025349	0,025304	0,030158	0,063325	0,016761	0,026411	0,012425	0	0,012372
k__Bacteria;p__Actinobacteria;c__Acidimicrobiia;o__Acidimicrobiales;f__C111;g__;s__	0,765136	0,91654	1,3188	2,633328	1,894374	1,393914	0,944571	1,140496	0,979033	1,746153	1,253068	1,389945	0,290992	0,499763	0,809662	1,148125	0,400563	0,886312	0,288016	0,42476

k__Bacteria;p__Actinobacteria;c__Acidimicrobiia;o__Acidimicrobiales;f__EB1017;g__;s__	0,461577	0,494171	0,091526	0,211829	0,229621	0,180921	0,266197	0,107438	0,220932	0,194487	0,297119	0,367554	0,126518	0,25419	0,316627	0,343599	0,426974	0,505281	0,187837	0,082478
k__Bacteria;p__Actinobacteria;c__Acidimicrobiia;o__Acidimicrobiales;f__Iamiaceae;g__;s__	0	0	0	0	0	0	0	0	0	0	0	0,004225	0	0	0,009046	0	0	0	0	0,004124
k__Bacteria;p__Actinobacteria;c__Acidimicrobiia;o__Acidimicrobiales;f__Iamiaceae;g__Iamia;s__	0,058217	0,130934	0,00416	0,012461	0,020502	0,016447	0,008587	0,008264	0,008664	0,033824	0,02153	0,042248	0,025304	0,017233	0,018093	0,016761	0,013205	0,016567	0	0,012372
k__Bacteria;p__Actinobacteria;c__Acidimicrobiia;o__Acidimicrobiales;f__Microthrixaceae;g__;s__	0	0	0	0	0,008201	0	0	0	0	0	0	0	0	0	0	0	0	0	0	0
k__Bacteria;p__Actinobacteria;c__Acidimicrobiia;o__Acidimicrobiales;f__koll13;g__;s__	0,191284	0,11404	0,037442	0,037382	0,077907	0,053454	0,094457	0,066116	0,047652	0,054964	0,077509	0,054922	0,016869	0,038775	0,085942	0,087995	0,118848	0,157382	0,008348	0,012372
k__Bacteria;p__Actinobacteria;c__Acidimicrobiia;o__Acidimicrobiales;f__ntu14;g__;s__	0	0	0	0	0	0	0	0	0,004332	0	0,004306	0	0	0	0,004523	0	0	0	0	0
k__Bacteria;p__Actinobacteria;c__Actinobacteria;o__Actinomycetales;Other;Other;Other	0,873253	0,937658	1,031743	1,067453	0,893882	0,555099	0,596797	0,347107	0,740773	0,410113	0,564096	0,785805	0,345816	0,603162	0,72372	0,783574	0,29492	0,617105	0,208707	0,305167
k__Bacteria;p__Actinobacteria;c__Actinobacteria;o__Actinomycetales;f__;g__;s__	0,336826	0,608211	0,3245	0,569031	0,615057	0,386513	0,57533	0,61157	0,41154	0,266362	0,46075	0,464723	0,333165	0,284348	0,578976	0,775194	0,255304	0,414164	0,254623	0,2763
k__Bacteria;p__Actinobacteria;c__Actinobacteria;o__Actinomycetales;f__Actinomycetaceae;g__Actinomyces;s__	0,004158	0,004224	0	0	0	0,016447	0,004294	0	0,008664	0,004228	0	0	0	0	0	0,00838	0	0,008283	0,025045	0
k__Bacteria;p__Actinobacteria;c__Actinobacteria;o__Actinomycetales;f__Actinosynnemataceae;Other;Other	0	0	0,00416	0	0	0	0	0	0,004332	0	0	0,00845	0,033738	0	0,036186	0,012571	0,013205	0,012425	0	0
k__Bacteria;p__Actinobacteria;c__Actinobacteria;o__Actinomycetales;f__Actinosynnemataceae;g__;s__	0,0499	0,054908	0,008321	0,008307	0,012301	0,028783	0,055816	0,024793	0,056316	0,02114	0,064591	0,059147	0,41751	0,021542	0,307581	0,079614	0,154063	0,057983	0,041741	0,061858
k__Bacteria;p__Actinobacteria;c__Actinobacteria;o__Actinomycetales;f__Actinosynnemataceae;g__Kibdelosporangium;s__	0	0	0	0	0	0	0,004294	0	0,012996	0	0	0	0	0	0	0,00419	0	0	0	0
k__Bacteria;p__Actinobacteria;c__Actinobacteria;o__Actinomycetales;f__Actinosynnemataceae;g__Lentzea;Other	0	0,008447	0	0,004154	0	0	0	0	0	0	0	0,004225	0,004217	0	0	0	0	0,008283	0	0
k__Bacteria;p__Actinobacteria;c__Actinobacteria;o__Actinomycetales;f__Bogoriellaceae;g__Georgenia;s__	0	0,008447	0,008321	0	0	0,024671	0,017174	0,004132	0	0	0	0	0	0	0	0	0	0	0	0
k__Bacteria;p__Actinobacteria;c__Actinobacteria;o__Actinomycetales;f__Brevibacteriaceae;g__Brevibacterium;s__	0	0	0	0,020768	0,008201	0	0	0	0	0	0	0,004225	0	0	0	0	0	0	0	0
k__Bacteria;p__Actinobacteria;c__Actinobacteria;o__Actinomycetales;f__Cellulomonadaceae;Other;Other	0	0	0	0	0	0	0,004294	0	0	0	0	0,004225	0	0	0	0	0	0	0	0
k__Bacteria;p__Actinobacteria;c__Actinobacteria;o__Actinomycetales;f__Cellulomonadaceae;g__Actinotalea;s__	0,004158	0	0,008321	0,008307	0	0,004112	0,012881	0,004132	0,004332	0,008456	0,004306	0,004225	0,012652	0	0,004523	0,016761	0,004402	0,004142	0	0,016496
k__Bacteria;p__Actinobacteria;c__Actinobacteria;o__Actinomycetales;f__Cellulomonadaceae;g__Cellulomonas;s__	0	0	0,00416	0	0	0	0	0	0	0,00845	0	0	0,00421	0,00430	0	0	0	0	0	0

										6			7	8						
k__Bacteria;p__Actinobacteria;c__Actinobacteria;o__Actinomycetales;f__Corynebacteriaceae;g__Corynebacterium;s__	0,004158	0,004224	0	0,087224	0,024602	0,061678	0,017174	0,004132	0,02166	0,016912	0,004306	0	0	0	0	0,016761	0	0,045558	0,100179	0,20207
k__Bacteria;p__Actinobacteria;c__Actinobacteria;o__Actinomycetales;f__Cryptosporangiaceae;g__;s__	0	0	0,00416	0	0	0	0	0	0	0	0	0	0	0	0	0	0	0	0	0,004124
k__Bacteria;p__Actinobacteria;c__Actinobacteria;o__Actinomycetales;f__Dermabacteraceae;g__Brachybacterium;Other	0,004158	0,004224	0	0	0	0	0	0	0	0	0	0	0,008435	0,004308	0	0	0	0	0	0
k__Bacteria;p__Actinobacteria;c__Actinobacteria;o__Actinomycetales;f__Dermacoccaceae;g__Dermacoccus;s__	0	0,004224	0	0	0	0	0,004294	0	0	0	0	0	0	0	0	0	0	0	0	0
k__Bacteria;p__Actinobacteria;c__Actinobacteria;o__Actinomycetales;f__Dietziaceae;Other;Other	0	0	0	0	0	0	0	0	0	0	0	0	0	0	0	0,00838	0	0,004142	0	0,008248
k__Bacteria;p__Actinobacteria;c__Actinobacteria;o__Actinomycetales;f__Dietziaceae;g__Dietzia;Other	0	0	0	0	0	0	0	0	0	0	0	0,004225	0	0,004308	0	0	0	0	0	0
k__Bacteria;p__Actinobacteria;c__Actinobacteria;o__Actinomycetales;f__Dietziaceae;g__Dietzia;s__	0,444943	0,215408	0,124808	0,033228	0,057405	0,851151	0,094457	0,119835	0,025992	0,033824	0,103346	0,101394	0,143387	0,103399	0,094988	0,276556	0,039616	0,008283	0,037567	0,086602
k__Bacteria;p__Actinobacteria;c__Actinobacteria;o__Actinomycetales;f__Frankiaceae;Other;Other	0	0	0	0	0	0	0	0	0	0,004228	0,012918	0	0	0	0	0	0	0	0	0
k__Bacteria;p__Actinobacteria;c__Actinobacteria;o__Actinomycetales;f__Frankiaceae;g__;s__	0,029108	0,021118	0	0	0	0	0,008587	0,008264	0,02166	0,012684	0,017224	0,012674	0	0,008617	0,01357	0,00419	0	0	0	0,004124
k__Bacteria;p__Actinobacteria;c__Actinobacteria;o__Actinomycetales;f__Frankiaceae;g__Frankia;s__	0	0	0	0	0	0	0	0	0	0	0	0	0	0	0	0	0,008804	0	0	0
k__Bacteria;p__Actinobacteria;c__Actinobacteria;o__Actinomycetales;f__Geodermatophilaceae;Other;Other	0,153859	0,152053	0,049923	0,103838	0,065606	0,139803	0,098751	0,152893	0,06498	0,173347	0,142101	0,240811	0,042173	0,146482	0,090465	0,217892	0,114447	0,07455	0,07096	0,272176
k__Bacteria;p__Actinobacteria;c__Actinobacteria;o__Actinomycetales;f__Geodermatophilaceae;g__;s__	0,174651	0,1605	0,099846	0,265825	0,168115	0,250822	0,201795	0,318182	0,186276	0,156435	0,176549	0,270384	0,13917	0,198182	0,203546	0,486067	0,189277	0,22779	0,158618	0,094849
k__Bacteria;p__Actinobacteria;c__Actinobacteria;o__Actinomycetales;f__Geodermatophilaceae;g__Geodermatophilus;Other	0	0,016895	0,016641	0,008307	0,008201	0	0	0	0,004332	0,012684	0,004306	0,00845	0	0,004308	0,009046	0,00419	0,008804	0,008283	0,004174	0,004124
k__Bacteria;p__Actinobacteria;c__Actinobacteria;o__Actinomycetales;f__Geodermatophilaceae;g__Geodermatophilus;s__	0	0	0	0,004154	0	0	0	0	0	0,004228	0	0	0	0,004308	0	0,00419	0	0	0	0
k__Bacteria;p__Actinobacteria;c__Actinobacteria;o__Actinomycetales;f__Geodermatophilaceae;g__Geodermatophilus;s__obscurus	0	0	0	0	0	0,008224	0,004294	0	0	0	0	0	0,004217	0	0	0	0	0	0	0
k__Bacteria;p__Actinobacteria;c__Actinobacteria;o__Actinomycetales;f__Geodermatophilaceae;g__Modestobacter;s__	0,008317	0,012671	0,012481	0,033228	0,016402	0,020559	0,008587	0,016529	0,017328	0,012684	0,034449	0,012674	0,008435	0,004308	0,040709	0,054473	0,008804	0,012425	0,020871	0,028867
k__Bacteria;p__Actinobacteria;c__Actinobacteria;o__Actinomycetales;f__Gordoniaceae;g__Gordonia;s__	0	0	0	0	0,0041	0,008224	0	0	0	0	0,008612	0,004225	0	0	0,004523	0,00419	0	0	0	0

k__Bacteria;p__Actinobacteria;c__Actinobacteria;o__Actinomycetales;f__Intrasporangiaceae;Other;Other	0,054059	0,021118	0	0,033228	0,057405	0,205592	0,038642	0,049587	0,008664	0,02114	0,012918	0,029573	0,084345	0,103399	0,004523	0,041902	0,013205	0,0497	0,033393	0,144336
k__Bacteria;p__Actinobacteria;c__Actinobacteria;o__Actinomycetales;f__Intrasporangiaceae;g__;s__	0,004158	0,004224	0	0	0,020502	0,004112	0,004294	0	0,02166	0	0	0	0	0,004308	0	0,00838	0	0	0	0
k__Bacteria;p__Actinobacteria;c__Actinobacteria;o__Actinomycetales;f__Intrasporangiaceae;g__Janibacter;s__	0	0	0	0	0	0	0,004294	0	0	0	0	0	0	0	0	0	0	0	0	0,004124
k__Bacteria;p__Actinobacteria;c__Actinobacteria;o__Actinomycetales;f__Intrasporangiaceae;g__Knoellia;Other	0	0	0	0	0	0	0	0	0	0	0,004306	0	0,021086	0	0	0	0,004402	0	0	0
k__Bacteria;p__Actinobacteria;c__Actinobacteria;o__Actinomycetales;f__Intrasporangiaceae;g__Knoellia;s__subterranea	0,008317	0,004224	0	0,004154	0	0,016447	0	0,004132	0	0	0	0,004225	0,012652	0	0	0,00838	0	0	0	0,004124
k__Bacteria;p__Actinobacteria;c__Actinobacteria;o__Actinomycetales;f__Kineosporiaceae;Other;Other	0,004158	0,021118	0,008321	0,004154	0	0	0,008587	0,008264	0	0	0	0,00845	0	0,004308	0,009046	0,00419	0	0	0	0,008248
k__Bacteria;p__Actinobacteria;c__Actinobacteria;o__Actinomycetales;f__Kineosporiaceae;g__;s__	0,004158	0	0,008321	0,008307	0,020502	0,004112	0	0	0,008664	0	0,004306	0,004225	0,004217	0,004308	0,004523	0,00838	0,004402	0,012425	0	0,008248
k__Bacteria;p__Actinobacteria;c__Actinobacteria;o__Actinomycetales;f__Kineosporiaceae;g__Kineococcus;Other	0,008317	0	0	0,012461	0,0041	0	0,004294	0,008264	0,008664	0	0,004306	0,00845	0	0,004308	0,009046	0,016761	0	0,008283	0,004174	0
k__Bacteria;p__Actinobacteria;c__Actinobacteria;o__Actinomycetales;f__Microbacteriaceae;Other;Other	0	0	0	0	0	0	0	0,004132	0	0	0	0,004225	0	0,004308	0	0	0	0	0,004174	0
k__Bacteria;p__Actinobacteria;c__Actinobacteria;o__Actinomycetales;f__Microbacteriaceae;g__;s__	0,112275	0,118263	0,008321	0,004154	0,127112	0,180921	0,313426	0,136364	0,06498	0,177575	0,068897	0,101394	0,189777	0,137866	0,022616	0,134088	0,013205	0,028992	0,697082	0,317539
k__Bacteria;p__Actinobacteria;c__Actinobacteria;o__Actinomycetales;f__Microbacteriaceae;g__Agrococcus;s__jenensis	0	0,012671	0	0	0	0,004112	0,017174	0	0,012996	0,004228	0	0,004225	0,008435	0,008617	0	0	0	0	0,008348	0,004124
k__Bacteria;p__Actinobacteria;c__Actinobacteria;o__Actinomycetales;f__Microbacteriaceae;g__Candidatus Aquiluna;s__rubra	0,004158	0	0	0,008307	0	0	0,004294	0	0	0	0	0	0,012652	0	0,004523	0	0	0	0,037567	0,115469
k__Bacteria;p__Actinobacteria;c__Actinobacteria;o__Actinomycetales;f__Microbacteriaceae;g__Candidatus Rhodoluna;s__	0	0	0	0	0	0	0,008587	0	0	0	0	0	0	0,004308	0	0	0	0	0	0,012372
k__Bacteria;p__Actinobacteria;c__Actinobacteria;o__Actinomycetales;f__Microbacteriaceae;g__Cryocola;s__	0	0	0	0	0	0,008224	0	0,008264	0	0	0	0	0	0	0	0	0	0	0	0
k__Bacteria;p__Actinobacteria;c__Actinobacteria;o__Actinomycetales;f__Microbacteriaceae;g__Microbacterium;s__	0	0	0	0	0	0	0,025761	0,016529	0	0,012684	0,02153	0,00845	0,004217	0,021542	0	0,00838	0,004402	0	0,033393	0,016496
k__Bacteria;p__Actinobacteria;c__Actinobacteria;o__Actinomycetales;f__Microbacteriaceae;g__Mycetocola;s__	0	0	0	0	0	0	0	0	0,004332	0	0	0,004225	0	0	0	0	0	0	0	0
k__Bacteria;p__Actinobacteria;c__Actinobacteria;o__Actinomycetales;f__Microbacteriaceae;g__Salinibacterium;s__	0	0	0	0	0	0,024671	0,004294	0	0	0,012684	0,008612	0,00845	0	0,008617	0	0,00419	0	0,004142	0,029219	0,004124
k__Bacteria;p__Actinobacteria;c__Actinobacteria;o__Actinomycetales;f__Micrococcaceae;Other;Other	0,00415	0	0	0	0	0	0,00429	0,01239	0,00433	0,00422	0,01291	0,02957	0,00421	0,01723	0,00904	0,02095	0,02200	0	0	0

	8						4	7	2	8	8	3	7	3	6	1	9			
k__Bacteria;p__Actinobacteria;c__Actinobacteria;o__Actinomycetales;f__Micrococcaceae;g__;s__	1,559381	0,819395	0,253775	0,593952	0,364934	1,151316	1,67876	1,35124	0,168948	1,627769	1,283211	1,774398	1,218792	1,051226	0,357337	1,40792	1,685888	0,269207	2,178904	1,195926
k__Bacteria;p__Actinobacteria;c__Actinobacteria;o__Actinomycetales;f__Micrococcaceae;g__Arthrobacter;Other	0,004158	0,004224	0,008321	0	0	0,004112	0,004294	0	0	0,025368	0,012918	0,021124	0,008435	0,008617	0,009046	0	0,008804	0	0,004174	0
k__Bacteria;p__Actinobacteria;c__Actinobacteria;o__Actinomycetales;f__Micrococcaceae;g__Arthrobacter;s__	0,103959	0,038013	0,016641	0,020768	0,0041	0,024671	0,042935	0,045455	0,004332	0,287502	0,030143	0,139417	0,033738	0,060316	0,01357	0,108946	0,026411	0,020708	0,045916	0,185575
k__Bacteria;p__Actinobacteria;c__Actinobacteria;o__Actinomycetales;f__Micrococcaceae;g__Kocuria;s__palustris	0	0	0	0,008307	0	0	0	0,008264	0	0,012684	0,004306	0	0,004217	0,004308	0	0	0	0	0,008348	0,028867
k__Bacteria;p__Actinobacteria;c__Actinobacteria;o__Actinomycetales;f__Micrococcaceae;g__Kocuria;s__rhizophila	0	0	0	0	0	0	0,004294	0	0	0	0	0	0	0	0	0	0	0	0	0,004124
k__Bacteria;p__Actinobacteria;c__Actinobacteria;o__Actinomycetales;f__Micrococcaceae;g__Microbispora;s__rosea	0,004158	0	0	0	0	0	0	0	0	0	0	0	0,008435	0	0	0	0	0	0,016697	0
k__Bacteria;p__Actinobacteria;c__Actinobacteria;o__Actinomycetales;f__Micrococcaceae;g__Micrococcus;Other	0,004158	0	0,00416	0,020768	0,024602	0,016447	0,004294	0,008264	0,004332	0,004228	0,017224	0,004225	0	0	0,004523	0,020951	0,008804	0,02485	0,029219	0,115469
k__Bacteria;p__Actinobacteria;c__Actinobacteria;o__Actinomycetales;f__Micrococcaceae;g__Nesterenkonia;s__	0,004158	0	0	0,020768	0	0	0	0,016529	0	0	0,004306	0,004225	0,004217	0	0	0,00838	0	0	0,012522	0,012372
k__Bacteria;p__Actinobacteria;c__Actinobacteria;o__Actinomycetales;f__Micrococcaceae;g__Rothia;s__dentocariosa	0	0	0	0,004154	0	0	0	0	0	0	0,004306	0	0	0	0	0	0	0	0,008348	0,008248
k__Bacteria;p__Actinobacteria;c__Actinobacteria;o__Actinomycetales;f__Micrococcaceae;g__Rothia;s__mucilaginosa	0	0	0	0,008307	0,0041	0,016447	0	0	0	0	0	0	0	0	0	0	0	0,016567	0	0,004124
k__Bacteria;p__Actinobacteria;c__Actinobacteria;o__Actinomycetales;f__Micromonosporaceae;Other;Other	0,004158	0	0,00416	0,008307	0,0041	0	0,012881	0	0,012996	0	0,004306	0,012674	0,004217	0	0,004523	0,00419	0,008804	0,016567	0	0
k__Bacteria;p__Actinobacteria;c__Actinobacteria;o__Actinomycetales;f__Micromonosporaceae;g__;s__	0,004158	0,008447	0,012481	0,058149	0,135312	0,061678	0,012881	0	0,004332	0	0,004306	0	0	0,004308	0	0,00419	0,013205	0,004142	0	0
k__Bacteria;p__Actinobacteria;c__Actinobacteria;o__Actinomycetales;f__Micromonosporaceae;g__Actinoplanes;s__	0	0	0	0	0	0	0	0	0,004332	0	0	0	0,004217	0	0	0	0	0	0	0
k__Bacteria;p__Actinobacteria;c__Actinobacteria;o__Actinomycetales;f__Micromonosporaceae;g__Catellatospora;s__	0,170492	0,08025	0	0,020768	0,057405	0,041118	0,708428	0,004132	0,15162	0,029596	0,034449	0,016899	0,084345	0,056008	0,022616	0,037712	0,079232	0,078691	0,091831	0,016496
k__Bacteria;p__Actinobacteria;c__Actinobacteria;o__Actinomycetales;f__Micromonosporaceae;g__Dactylosporangium;s__	0,008317	0	0	0	0	0	0	0	0	0	0	0	0	0	0	0	0	0	0	0
k__Bacteria;p__Actinobacteria;c__Actinobacteria;o__Actinomycetales;f__Mycobacteriaceae;g__Mycobacterium;s__	0,016633	0,004224	0,083205	0,012461	0,016402	0,020559	0,012881	0	0	0,008456	0,008612	0,004225	0,004217	0,004308	0	0	0	0	0	0
k__Bacteria;p__Actinobacteria;c__Actinobacteria;o__Actinomycetales;f__Nakamurellaceae;g__;s__	0,004158	0,004224	0	0	0	0	0	0,008264	0	0	0	0	0	0	0	0	0	0	0	0

k__Bacteria;p__Actinobacteria;c__Actinobacteria;o__Actinomycetales;f__Nocardiaceae;g__Nocardia;s__	0,008317	0,008447	0	0,008307	0,0041	0,008224	0	0	0	0	0,004306	0,004225	0,008435	0,021542	0	0	0,004402	0	0	0,008248
k__Bacteria;p__Actinobacteria;c__Actinobacteria;o__Actinomycetales;f__Nocardiaceae;g__Rhodococcus;s__	0,008317	0,021118	0,008321	0,045689	0,036903	0,061678	0,021468	1,46281	0,017328	0,033824	0,012918	0,004225	0,021086	0,116324	0,004523	0,012571	0,026411	0,02485	0,016697	0,070106
k__Bacteria;p__Actinobacteria;c__Actinobacteria;o__Actinomycetales;f__Nocardiaceae;g__Rhodococcus;s__fascians	0,120592	0,050684	0,016641	0,024921	0,016402	0,078125	0,055816	0,028926	0,038988	0,038052	0,064591	0,076046	0,067476	0,094783	0,018093	0,050283	0,026411	0,095258	0,091831	0,255681
k__Bacteria;p__Actinobacteria;c__Actinobacteria;o__Actinomycetales;f__Nocardiaceae;g__Rhodococcus;s__ruber	0,004158	0,008447	0,016641	0	0,0041	0	0,004294	0	0,004332	0,004228	0,008612	0,016899	0,067476	0,017233	0,027139	0,00419	0,017607	0	0	0,004124
k__Bacteria;p__Actinobacteria;c__Actinobacteria;o__Actinomycetales;f__Nocardioidaceae;Other;Other	0,004158	0,029566	0,00416	0,004154	0,0041	0	0	0,004132	0,012996	0	0,030143	0,004225	0,025304	0,004308	0,01357	0	0,004402	0	0	0,004124
k__Bacteria;p__Actinobacteria;c__Actinobacteria;o__Actinomycetales;f__Nocardioidaceae;g__;s__	6,146041	9,972124	18,00973	7,688154	10,8824	3,782895	4,731441	3,871901	11,81771	7,749873	7,531327	7,296155	17,98667	7,268106	12,37109	9,231092	7,350999	3,843446	7,830697	6,041486
k__Bacteria;p__Actinobacteria;c__Actinobacteria;o__Actinomycetales;f__Nocardioidaceae;g__Actinopolymorpha;s__	0	0	0,00416	0	0	0	0	0	0	0	0	0	0	0	0	0,00419	0	0	0	0
k__Bacteria;p__Actinobacteria;c__Actinobacteria;o__Actinomycetales;f__Nocardioidaceae;g__Aeromicrobium;s__	0,020792	0,029566	0,029122	0,07061	0,118911	0,032895	0,081577	0,028926	0,034656	0,084559	0,043061	0,012674	0,033738	0,008617	0,018093	0,054473	0,013205	0,020708	0,004174	0,032991
k__Bacteria;p__Actinobacteria;c__Actinobacteria;o__Actinomycetales;f__Nocardioidaceae;g__Friedmanniella;s__	0	0,004224	0	0	0	0	0,004294	0	0,004332	0	0	0,00845	0	0,004308	0,004523	0	0	0,004142	0	0
k__Bacteria;p__Actinobacteria;c__Actinobacteria;o__Actinomycetales;f__Nocardioidaceae;g__Kribbella;s__	0	0	0	0,004154	0	0	0,004294	0	0	0	0	0	0	0	0	0	0	0	0	0,008248
k__Bacteria;p__Actinobacteria;c__Actinobacteria;o__Actinomycetales;f__Nocardioidaceae;g__Nocardioides;s__	0	0,004224	0,008321	0	0,008201	0	0,017174	0	0,017328	0,029596	0,012918	0,016899	0,021086	0,012925	0,027139	0,00419	0,004402	0	0,012522	0,008248
k__Bacteria;p__Actinobacteria;c__Actinobacteria;o__Actinomycetales;f__Nocardioidaceae;g__Nocardioides;s__plantarum	0,004158	0,004224	0	0,004154	0,0041	0	0	0	0,004332	0,004228	0	0	0,037955	0,004308	0,009046	0,00838	0	0	0	0
k__Bacteria;p__Actinobacteria;c__Actinobacteria;o__Actinomycetales;f__Nocardioidaceae;g__Pimelobacter;s__	0	0,021118	0,008321	0,016614	0,012301	0,012336	0,021468	0,049587	0,017328	0,004228	0,004306	0,021124	0,04639	0,02585	0,004523	0,020951	0,017607	0,008283	0,008348	0,016496
k__Bacteria;p__Actinobacteria;c__Actinobacteria;o__Actinomycetales;f__Nocardiopsaceae;g__;s__	0	0	0	0	0	0,004112	0,030055	0	0	0	0,017224	0	0,016869	0	0	0	0	0	0	0
k__Bacteria;p__Actinobacteria;c__Actinobacteria;o__Actinomycetales;f__Promicromonosporaceae;g__Xylanimicrobium;Other	0	0	0	0	0	0	0	0	0	0	0	0,004225	0	0	0,004523	0	0	0	0,045916	0,024743
k__Bacteria;p__Actinobacteria;c__Actinobacteria;o__Actinomycetales;f__Propionibacteriaceae;g__;s__	0,004158	0	0	0	0	0	0	0	0	0	0,012918	0,004225	0,008435	0,004308	0,004523	0,00419	0,004402	0	0	0,008248
k__Bacteria;p__Actinobacteria;c__Actinobacteria;o__Actinomycetales;f__Propionibacteriaceae;g__Propionibacterium;s__acnes	0	0	0	0,012461	0,0041	0,004112	0,012881	0,008264	0,073644	0,008456	0,017224	0,00845	0,029521	0,004308	0,01357	0,041902	0	0,045558	0,112702	0,107221
k__Bacteria;p__Actinobacteria;c__Actinobacteria;o__Actinomycetales;f__Pseudonocardiaceae;Other;Other	0,004158	0,008447	0,008321	0	0,0041	0	0,004294	0	0,012996	0	0,004306	0,029573	0,063259	0,008617	0,054279	0	0,008804	0,004142	0	0

k__Bacteria;p__Actinobacteria;c__Actinobacteria;o__Actinomycetales;f__Pseudonocardiaceae;g__;s__	0,42831	0,143605	0,474269	0,693637	0,332131	0,333059	0,214675	0,041322	0,207936	0,071876	0,155019	0,118293	0,109649	0,163715	0,271395	0,255604	0,299322	0,207082	0,705431	0,136088
k__Bacteria;p__Actinobacteria;c__Actinobacteria;o__Actinomycetales;f__Pseudonocardiaceae;g__Actinomycetospora;s__	0	0	0	0	0	0	0,004294	0	0	0	0	0,00845	0	0	0	0	0,008804	0	0	0
k__Bacteria;p__Actinobacteria;c__Actinobacteria;o__Actinomycetales;f__Pseudonocardiaceae;g__Amycolatopsis;Other	0	0	0	0	0	0	0	0	0	0	0	0	0	0	0,009046	0	0	0,004142	0	0
k__Bacteria;p__Actinobacteria;c__Actinobacteria;o__Actinomycetales;f__Pseudonocardiaceae;g__Amycolatopsis;s__	0,070692	0,815171	0,037442	0,103838	0,164015	0,160362	0,188914	0,086777	0,844741	0,122611	0,137794	0,274609	1,033232	0,172332	1,230324	0,243034	0,101241	0,25264	0,125224	0,136088
k__Bacteria;p__Actinobacteria;c__Actinobacteria;o__Actinomycetales;f__Pseudonocardiaceae;g__Pseudonocardia;s__	0,016633	0,4815	0,083205	0,22429	0,098409	0,057566	0,150273	0,367769	0,160284	0,084559	0,077509	0,126743	0,088563	0,017233	0,16736	0,243034	0,013205	0,0497	0,05009	0,280424
k__Bacteria;p__Actinobacteria;c__Actinobacteria;o__Actinomycetales;f__Pseudonocardiaceae;g__Pseudonocardia;s__halophobica	0	0	0	0	0	0,004112	0,008587	0	0	0	0	0,004225	0	0	0	0	0	0	0	0
k__Bacteria;p__Actinobacteria;c__Actinobacteria;o__Actinomycetales;f__Pseudonocardiaceae;g__Saccharopolyspora;Other	0	0,004224	0	0	0	0	0	0	0,008664	0	0	0,004225	0	0	0	0,00419	0	0	0	0
k__Bacteria;p__Actinobacteria;c__Actinobacteria;o__Actinomycetales;f__Pseudonocardiaceae;g__Saccharopolyspora;s__	0,004158	0,012671	0	0	0	0,004112	0,008587	0,004132	0,012996	0	0,004306	0,004225	0,033738	0	0,113081	0,025141	0,008804	0,016567	0,004174	0,004124
k__Bacteria;p__Actinobacteria;c__Actinobacteria;o__Actinomycetales;f__Sporichthyaceae;g__;s__	0,548902	1,127724	0,528352	0,739325	0,602755	0,46875	0,712722	0,570248	0,684457	0,397429	0,55979	0,566117	0,708502	0,564388	1,05844	1,500105	0,413769	1,014703	0,567684	0,841272
k__Bacteria;p__Actinobacteria;c__Actinobacteria;o__Actinomycetales;f__Sporichthyaceae;g__Sporichthya;s__	0	0	0	0	0	0	0	0	0	0	0,004306	0	0	0	0	0,00419	0,004402	0	0	0
k__Bacteria;p__Actinobacteria;c__Actinobacteria;o__Actinomycetales;f__Streptomycetaceae;Other;Other	0,004158	0,025342	0,024962	0	0,0041	0	0,017174	0	0,017328	0,012684	0,012918	0,029573	0,168691	0,047391	0,01357	0,016761	0,030813	0,008283	0,012522	0
k__Bacteria;p__Actinobacteria;c__Actinobacteria;o__Actinomycetales;f__Streptomycetaceae;g__;s__	0,02495	0,088697	0,00416	0,016614	0,012301	0,008224	0,017174	0,004132	0,012996	0,016912	0,068897	0,050697	0,23195	0,129249	0,022616	0,050283	0,030813	0,033133	0,096005	0,07423
k__Bacteria;p__Actinobacteria;c__Actinobacteria;o__Actinomycetales;f__Streptomycetaceae;g__Streptacidiphilus;s__	0	0	0	0	0	0	0	0	0	0	0	0	0,004217	0	0	0	0	0	0,004174	0
k__Bacteria;p__Actinobacteria;c__Actinobacteria;o__Actinomycetales;f__Streptomycetaceae;g__Streptomyces;Other	0,008317	0,033789	0,00416	0	0,0041	0,008224	0,012881	0	0,008664	0,029596	0,038755	0,012674	0,080128	0,056008	0,009046	0,020951	0,017607	0	0,016697	0,012372
k__Bacteria;p__Actinobacteria;c__Actinobacteria;o__Actinomycetales;f__Streptomycetaceae;g__Streptomyces;s__	0,087325	0,249197	0,062404	0,103838	0,057405	0,287829	0,133099	0,070248	0,147288	0,27059	0,602851	0,544994	2,163462	1,163242	0,221639	0,427404	0,396162	0,318907	1,786534	1,142315
k__Bacteria;p__Actinobacteria;c__Actinobacteria;o__Actinomycetales;f__Streptosporangiaceae;Other;Other	0	0	0	0	0	0	0	0	0,004332	0	0	0	0	0,004308	0	0	0,004402	0	0	0
k__Bacteria;p__Actinobacteria;c__Actinobacteria;o__Actinomycetales;f__Thermomonosporaceae;Other;Other	0,02495	0,033789	0,008321	0,004154	0,008201	0,020559	0,042935	0,004132	0,02166	0,004228	0,017224	0,033798	0,113866	0,008617	0,027139	0,00419	0,074831	0,012425	0,07096	0
k__Bacteria;p__Actinobacteria;c__Actinobacteria;o__Actinomycetales;f__Thermomonosporaceae;g__;s__	0,00415	0	0	0	0	0	0	0	0	0	0	0,00422	0	0	0	0	0,01320	0	0	0

	8											5					5			
k__Bacteria;p__Actinobacteria;c__Actinobacteria;o__Actinomycetales;f__Thermomonosporaceae;g__Actinomadura;Other	0	0	0,020801	0	0	0,004112	0	0	0	0	0	0	0,008435	0	0	0,00419	0	0	0	0
k__Bacteria;p__Actinobacteria;c__Actinobacteria;o__Actinomycetales;f__Thermomonosporaceae;g__Actinomadura;s__vinacea	0,008317	0	0	0	0	0,004112	0,004294	0	0,008664	0,004228	0,008612	0,012674	0,025304	0,004308	0,004523	0	0,030813	0,004142	0,020871	0
k__Bacteria;p__Actinobacteria;c__Actinobacteria;o__Actinomycetales;f__Williamsiaceae;g__Williamsia;s__	0,008317	0,008447	0	0	0,016402	0,04523	0,223262	0,004132	0,004332	0,004228	0	0,012674	0,008435	0,004308	0,009046	0,00838	0	0,033133	0,008348	0,012372
k__Bacteria;p__Actinobacteria;c__Actinobacteria;o__Bifidobacteriales;f__Bifidobacteriaceae;Other;Other	0,008317	0	0	0,004154	0	0,004112	0	0	0	0	0	0	0	0	0	0	0	0	0	0,012372
k__Bacteria;p__Actinobacteria;c__Actinobacteria;o__Bifidobacteriales;f__Bifidobacteriaceae;g__Bifidobacterium;s__longum	0	0	0	0	0	0	0	0,004132	0	0	0	0	0	0	0	0,046093	0	0	0	0
k__Bacteria;p__Actinobacteria;c__Actinobacteria;o__Micrococcales;f__;g__;s__	0	0	0	0	0	0	0	0	0	0	0	0	0	0	0	0	0	0,008283	0	0
k__Bacteria;p__Actinobacteria;c__Coriobacteriia;o__Coriobacteriales;f__Coriobacteriaceae;g__Atopobium;s__	0	0	0	0	0	0	0	0	0	0	0	0	0	0	0	0	0	0	0	0,012372
k__Bacteria;p__Actinobacteria;c__Coriobacteriia;o__Coriobacteriales;f__Coriobacteriaceae;g__Collinsella;s__aerofaciens	0	0	0	0	0	0	0	0,004132	0	0	0	0	0	0	0	0,00838	0	0	0,004174	0
k__Bacteria;p__Actinobacteria;c__MB-A2-108;o__;f__;g__;s__	0,166334	0,084474	0	0,004154	0,016402	0,016447	0,047229	0,024793	0,038988	0,046508	0,081815	0,046472	0,037955	0,056008	0,031663	0,037712	0,044018	0,082833	0,029219	0,024743
k__Bacteria;p__Actinobacteria;c__MB-A2-108;o__0319-7L14;f__;g__;s__	2,09165	1,875317	1,585056	3,621864	3,620633	1,957237	1,713108	0,896694	1,57685	1,014713	1,614778	1,440642	0,839238	1,171858	2,012846	1,797612	1,575843	1,967281	0,642818	0,597963
k__Bacteria;p__Actinobacteria;c__Nitriliruptoria;o__Euzebyales;f__Euzebyaceae;g__Euzebya;s__	0,145542	0,11404	0,141449	0,191062	0,164015	0,086349	0,163153	0,194215	0,181944	0,080331	0,193773	0,215463	0,067476	0,172332	0,262348	0,217892	0,101241	0,219507	0,116876	0,078354
k__Bacteria;p__Actinobacteria;c__Rubrobacteria;o__Rubrobacterales;f__Rubrobacteraceae;g__;s__	0,012475	0,008447	0,00416	0	0	0,004112	0,012881	0	0,030324	0,004228	0,02153	0,00845	0,008435	0	0,004523	0,00838	0,004402	0,012425	0,020871	0,008248
k__Bacteria;p__Actinobacteria;c__Rubrobacteria;o__Rubrobacterales;f__Rubrobacteraceae;g__Rubrobacter;s__	1,014637	0,633553	0,49923	2,940688	1,877973	0,884046	0,820059	0,818182	0,857737	0,849822	0,968867	0,785805	0,299426	0,745336	0,805138	1,23612	0,523814	1,528267	0,538465	0,560848
k__Bacteria;p__Actinobacteria;c__Thermoleophilia;o__Gaiellales;Other;Other;Other	0,004158	0	0,00416	0,004154	0,0041	0	0	0	0	0,004228	0	0	0	0,004308	0	0,00419	0,004402	0,004142	0	0
k__Bacteria;p__Actinobacteria;c__Thermoleophilia;o__Gaiellales;f__;g__;s__	0,0499	0,101368	0,066564	0,157834	0,168115	0,061678	0,055816	0,066116	0,047652	0,06342	0,094734	0,042248	0,016869	0,02585	0,076895	0,071234	0,04842	0,053841	0,041741	0,041239
k__Bacteria;p__Actinobacteria;c__Thermoleophilia;o__Gaiellales;f__AK1AB1_02E;g__;s__	0,087325	0,084474	0,041603	0,15368	0,135312	0,131579	0,111631	0,165289	0,121296	0,088787	0,107652	0,156316	0,059042	0,099091	0,162837	0,155039	0,105643	0,231932	0,05009	0,05361
k__Bacteria;p__Actinobacteria;c__Thermoleophilia;o__Gaiellales;f__Gaiellaceae;g__;s__	10,70775	7,750465	9,356409	6,313341	7,347876	6,949013	7,779829	5,450413	8,339109	4,177237	6,919864	6,370934	3,778677	4,963164	10,55726	7,006076	8,662734	8,556637	3,510456	2,730009

k__Bacteria;p__Actinobacteria;c__Thermoleophilia;o__Solirubrobacterales;Other;Other;Other	0,083167	0,046461	0,054083	0,08307	0,086108	0,041118	0,07299	0,041322	0,155952	0,038052	0,103346	0,08872	0,050607	0,077549	0,189976	0,163419	0,136456	0,165666	0,041741	0,037115
k__Bacteria;p__Actinobacteria;c__Thermoleophilia;o__Solirubrobacterales;f__;g__;s__	2,715403	1,410711	1,14407	1,686327	2,046088	1,418586	2,786484	1,483471	3,30532	1,492474	2,807561	2,670046	1,648954	2,51174	3,383391	3,339619	2,856766	4,899565	1,88254	1,666048
k__Bacteria;p__Actinobacteria;c__Thermoleophilia;o__Solirubrobacterales;f__Conexibacteraceae;Other;Other	0,02495	0,021118	0,00416	0,008307	0,008201	0,016447	0,012881	0,004132	0,02166	0	0,008612	0,016899	0,012652	0,030158	0,004523	0,00838	0,008804	0,020708	0,012522	0,032991
k__Bacteria;p__Actinobacteria;c__Thermoleophilia;o__Solirubrobacterales;f__Conexibacteraceae;g__;s__	0,382568	0,118263	0,128968	0,282439	0,225521	0,123355	0,437937	0,132231	0,454861	0,169119	0,297119	0,240811	0,206646	0,357589	0,398046	0,486067	0,347742	0,592255	0,283842	0,354654
k__Bacteria;p__Actinobacteria;c__Thermoleophilia;o__Solirubrobacterales;f__Conexibacteraceae;g__Conexibacter;s__	0	0	0	0	0	0	0,004294	0	0	0	0,004306	0,004225	0	0,008617	0,004523	0	0	0	0	0
k__Bacteria;p__Actinobacteria;c__Thermoleophilia;o__Solirubrobacterales;f__Patulibacteraceae;g__;s__	0,083167	0,11404	0,058244	0,203522	0,176316	0,123355	0,163153	0,103306	0,077976	0,093015	0,111958	0,109844	0,101215	0,077549	0,15379	0,146658	0,070429	0,178091	0,037567	0,078354
k__Bacteria;p__Actinobacteria;c__Thermoleophilia;o__Solirubrobacterales;f__Patulibacteraceae;g__Patulibacter;s__	0,008317	0	0	0	0,024602	0,008224	0,004294	0	0,004332	0,016912	0	0	0,004217	0	0	0,00419	0	0	0	0
k__Bacteria;p__Actinobacteria;c__Thermoleophilia;o__Solirubrobacterales;f__Solirubrobacteraceae;Other;Other	0,004158	0,004224	0	0	0,0041	0	0	0	0,004332	0	0	0,004225	0	0,004308	0,009046	0	0	0,004142	0	0
k__Bacteria;p__Actinobacteria;c__Thermoleophilia;o__Solirubrobacterales;f__Solirubrobacteraceae;g__;s__	0,607119	0,35479	0,449307	0,893005	0,75857	0,287829	0,613971	0,342975	0,701785	0,300186	0,551178	0,447824	0,269906	0,469605	0,655871	0,729101	0,404965	0,853179	0,313061	0,391769
k__Bacteria;p__Actinobacteria;c__Thermoleophilia;o__Solirubrobacterales;f__Solirubrobacteraceae;g__Solirubrobacter;s__	0	0	0	0	0	0	0	0	0	0	0	0	0,004217	0	0,004523	0	0	0	0	0
k__Bacteria;p__Armatimonadetes;c__0319-6E2;o__;f__;g__;s__	0,020792	0,004224	0,00416	0	0,008201	0	0,021468	0,041322	0,02166	0,033824	0,02153	0,00845	0,008435	0,008617	0,01357	0,041902	0,044018	0,041416	0,008348	0
k__Bacteria;p__Armatimonadetes;c__Armatimonadia;o__Armatimonadales;f__Armatimonadaceae;g__;s__	0,848303	0,223855	0,98598	0,398737	0,22142	0,242599	0,455111	0,280992	0,515509	0,596144	0,137794	0,185889	0,215081	0,172332	0,235209	0,155039	0,448983	0,356181	0,321409	0,131964
k__Bacteria;p__Armatimonadetes;c__Chthonomonadetes;o__SJA-22;f__;g__;s__	0,012475	0,004224	0	0	0	0	0,017174	0,008264	0	0,004228	0	0,00845	0	0	0	0	0	0,004142	0,004174	0
k__Bacteria;p__Armatimonadetes;c__OPB50;o__;f__;g__;s__	0,008317	0,008447	0,024962	0,008307	0	0,008224	0,012881	0,004132	0,012996	0,012684	0,02153	0,021124	0,012652	0,017233	0,01357	0,016761	0,004402	0,004142	0,033393	0
k__Bacteria;p__Armatimonadetes;c__[Fimbriimonadia];o__[Fimbriimonadales];f__[Fimbriimonadaceae];g__Fimbriimonas;s__	0	0,004224	0	0	0	0	0	0	0	0	0	0	0	0	0	0	0,004402	0	0	0
k__Bacteria;p__BRC1;c__PRR-11;o__;f__;g__;s__	0,033267	0,016895	0,33282	0,029075	0,016402	0,008224	0,012881	0,012397	0,008664	0,025368	0,034449	0,038023	0,012652	0	0,022616	0,041902	0,004402	0,016567	0	0
k__Bacteria;p__Bacteroidetes;c__Bacteroidia;o__Bacteroidales;f__Bacteroidaceae;g__Bacteroides;s__	0	0	0	0	0	0	0	0	0	0	0	0	0	0,004308	0,004523	0,00419	0	0	0,045916	0
k__Bacteria;p__Bacteroidetes;c__Bacteroidia;o__Bacteroidales;f__Prevotellaceae;g__Prevotella;s__	0	0	0	0	0	0	0	0	0	0	0	0	0	0,00430	0	0	0	0	0,00834	0,00412

														8					8	4
k__Bacteria;p__Bacteroidetes;c__Bacteroidia;o__Bacteroidales;f__Prevotellaceae;g__Prevotella;s__copri	0	0	0	0	0	0	0	0	0	0	0	0	0	0	0	0,033522	0	0	0	0
k__Bacteria;p__Bacteroidetes;c__Bacteroidia;o__Bacteroidales;f__Prevotellaceae;g__Prevotella;s__nanceiensis	0	0	0	0	0	0	0	0	0	0	0	0	0	0	0	0	0	0	0,012522	0
k__Bacteria;p__Bacteroidetes;c__Bacteroidia;o__Bacteroidales;f__[Paraprevotellaceae];g__[Prevotella];s__	0	0	0	0,004154	0	0	0	0	0	0	0	0	0	0	0	0	0	0	0	0,012372
k__Bacteria;p__Bacteroidetes;c__Bacteroidia;o__Bacteroidales;f__[Paraprevotellaceae];g__[Prevotella];s__tannerae	0	0,004224	0	0,004154	0	0	0	0	0	0	0	0	0	0	0	0	0	0	0,012522	0
k__Bacteria;p__Bacteroidetes;c__Cytophagia;o__Cytophagales;f__;g__;s__	0,0499	0,021118	0,024962	0	0,008201	0	0,051522	0,016529	0,012996	0,27059	0,030143	0,012674	0,016869	0,012925	0,027139	0,025141	0,211286	0,028992	0,509246	0,065982
k__Bacteria;p__Bacteroidetes;c__Cytophagia;o__Cytophagales;f__Cytophagaceae;g__;s__	0,016633	0,012671	0,041603	0,012461	0,0041	0,004112	0,025761	0,012397	0,02166	0	0,004306	0,025349	0,004217	0,017233	0,018093	0,016761	0,013205	0,016567	0,020871	0,012372
k__Bacteria;p__Bacteroidetes;c__Cytophagia;o__Cytophagales;f__Cytophagaceae;g__Adhaeribacter;s__	0	0,004224	0	0	0,0041	0	0,008587	0	0	0,008456	0	0,004225	0	0	0	0	0,004402	0,008283	0,029219	0,020619
k__Bacteria;p__Bacteroidetes;c__Cytophagia;o__Cytophagales;f__Cytophagaceae;g__Dyadobacter;s__	0	0	0	0	0	0	0	0	0,004332	0	0	0	0,004217	0	0	0	0	0	0	0
k__Bacteria;p__Bacteroidetes;c__Cytophagia;o__Cytophagales;f__Cytophagaceae;g__Hymenobacter;s__	0,041583	0,025342	0,008321	0,020768	0,008201	0,012336	0,030055	0	0,056316	0,04228	0,017224	0,016899	0,025304	0,012925	0,018093	0,020951	0,026411	0,053841	0,079309	0,049487
k__Bacteria;p__Bacteroidetes;c__Cytophagia;o__Cytophagales;f__Cytophagaceae;g__Rhodocytophaga;s__	0,0499	0,025342	0,058244	0,087224	0,008201	0,004112	0,090164	0,016529	0,077976	0,109927	0,02153	0,054922	0,088563	0,038775	0,036186	0,075424	0,136456	0,078691	0,146095	0,090725
k__Bacteria;p__Bacteroidetes;c__Cytophagia;o__Cytophagales;f__Cytophagaceae;g__Rudanella;s__	0,016633	0	0	0	0	0	0,021468	0,012397	0,008664	0,025368	0,025836	0,016899	0,016869	0,064625	0,009046	0,029332	0,013205	0,07455	0,012522	0,061858
k__Bacteria;p__Bacteroidetes;c__Cytophagia;o__Cytophagales;f__Cytophagaceae;g__Spirosoma;s__	0	0	0,00416	0	0	0	0	0	0	0,004228	0	0	0,004217	0	0,004523	0	0	0	0	0,012372
k__Bacteria;p__Bacteroidetes;c__Cytophagia;o__Cytophagales;f__Cytophagaceae;g__Sporocytophaga;s__	0,091484	0,012671	0,016641	0,029075	0,077907	0,008224	0,103044	0,03719	0,04332	0,084559	0,012918	0,038023	0,008435	0	0,040709	0,016761	0,026411	0,057983	0,020871	0,020619
k__Bacteria;p__Bacteroidetes;c__Cytophagia;o__Cytophagales;f__Flammeovirgaceae;g__;s__	0	0	0	0	0	0,008224	0	0	0	0	0	0	0	0	0	0	0	0	0	0
k__Bacteria;p__Bacteroidetes;c__Flavobacteriia;o__Flavobacteriales;f__Flavobacteriaceae;g__Flavobacterium;s__	0,004158	0	0	0	0	0,004112	0,008587	0,004132	0,008664	0,004228	0	0	0	0	0	0,020951	0	0,004142	0,045916	0,086602
k__Bacteria;p__Bacteroidetes;c__Flavobacteriia;o__Flavobacteriales;f__[Weeksellaceae];g__Chryseobacterium;s__	0	0	0	0	0,0041	0	0	0,008264	0	0,008456	0	0	0	0	0	0,00419	0	0,016567	0	0,008248
k__Bacteria;p__Bacteroidetes;c__Flavobacteriia;o__Flavobacteriales;f__[Weeksellaceae];g__Cloacibacterium;s__	0	0	0	0	0	0	0,012881	0	0,004332	0	0	0	0	0,004308	0	0,00419	0	0	0	0

k__Bacteria;p__Bacteroidetes;c__Flavobacteriia;o__Flavobacteriales;f__[Weeksellaceae];g__Wautersiella;s__	0	0	0	0	0	0	0	0	0	0	0	0	0	0	0	0	0	0	0,012522	0
k__Bacteria;p__Bacteroidetes;c__Sphingobacteriia;o__Sphingobacteriales;f__;g__;s__	0	0	2,845613	0,710251	1,098901	0,008224	0,124512	0,438017	0	1,391003	0	0,312632	0	0	0	0	0,026411	0,198799	0	0,008248
k__Bacteria;p__Bacteroidetes;c__Sphingobacteriia;o__Sphingobacteriales;f__Sphingobacteriaceae;g__;s__	0	0	0	0	0	0	0	0	0	0	0	0	0	0	0	0	0	0	0	0,008248
k__Bacteria;p__Bacteroidetes;c__Sphingobacteriia;o__Sphingobacteriales;f__Sphingobacteriaceae;g__Pedobacter;s__	0	0	0	0	0	0	0	0	0	0	0	0	0	0,008617	0	0	0	0	0,016697	0,008248
k__Bacteria;p__Bacteroidetes;c__[Saprospirae];o__[Saprospirales];f__Chitinophagaceae;g__;s__	0,02495	0,021118	0,033282	0,029075	0,0041	0,049342	0,068696	0,028926	0,056316	0,050736	0,008612	0,046472	0,033738	0,034466	0,031663	0,058663	0,061625	0,02485	0,05009	0,008248
k__Bacteria;p__Bacteroidetes;c__[Saprospirae];o__[Saprospirales];f__Chitinophagaceae;g__Flavisolibacter;s__	0,079009	0,063355	0,049923	0,058149	0,041004	0,016447	0,120218	0,082645	0,056316	0,097243	0,038755	0,054922	0,134953	0,068933	0,081418	0,113136	0,167268	0,070408	0,329757	0,14846
k__Bacteria;p__Bacteroidetes;c__[Saprospirae];o__[Saprospirales];f__Chitinophagaceae;g__Sediminibacterium;s__	0	0	0	0	0	0	0	0,008264	0	0	0	0	0	0	0	0	0	0	0	0,004124
k__Bacteria;p__Bacteroidetes;c__[Saprospirae];o__[Saprospirales];f__Chitinophagaceae;g__Segetibacter;s__	0	0	0,00416	0	0	0,004112	0,004294	0	0	0,004228	0	0	0,004217	0,004308	0,004523	0,00419	0	0,004142	0,041741	0
k__Bacteria;p__Chloroflexi;c__;o__;f__;g__;s__	0,32851	0,190066	0,137288	0,311514	0,196818	0,259046	0,141686	0,123967	0,12996	0,29173	0,124876	0,109844	0,156039	0,120632	0,140221	0,125707	0,17167	0,323048	0,07096	0,090725
k__Bacteria;p__Chloroflexi;c__Anaerolineae;o__Ardenscatenales;f__Ardenscatenaceae;g__Ardenscatena;s__	0,041583	0,038013	0,054083	0,062303	0,069706	0,041118	0,017174	0,024793	0,06498	0,012684	0,047367	0,08027	0,021086	0,017233	0,031663	0,033522	0,04842	0,02485	0,004174	0,020619
k__Bacteria;p__Chloroflexi;c__Anaerolineae;o__Caldilineales;f__Caldilineaceae;g__;s__	0,016633	0,033789	0,041603	0,08307	0,028703	0,020559	0,017174	0,004132	0,034656	0,025368	0,025836	0,021124	0,008435	0,021542	0,018093	0,012571	0,039616	0,020708	0,012522	0,012372
k__Bacteria;p__Chloroflexi;c__Anaerolineae;o__H39;f__;g__;s__	0,004158	0	0	0	0	0	0	0	0	0	0	0	0	0,004308	0,004523	0	0	0	0	0
k__Bacteria;p__Chloroflexi;c__C0119;o__;f__;g__;s__	0	0,004224	0	0	0,0041	0	0	0	0	0	0	0,004225	0	0	0	0	0	0	0	0,008248
k__Bacteria;p__Chloroflexi;c__Chloroflexi;o__AKIW781;f__;g__;s__	0,711078	0,553303	0,403545	2,367503	1,484337	0,953947	0,682667	0,68595	0,593485	0,613056	0,577014	0,498521	0,193995	0,460988	0,651348	0,745862	0,413769	0,646097	0,192011	0,309291
k__Bacteria;p__Chloroflexi;c__Chloroflexi;o__Chloroflexales;f__Chloroflexaceae;Other;Other	0	0	0	0	0,008201	0	0	0	0	0	0	0	0	0	0	0	0	0	0	0
k__Bacteria;p__Chloroflexi;c__Chloroflexi;o__Herpetosiphonales;f__;g__;s__	0,187126	0,046461	0,116487	0,107991	0,073807	0,185033	0,669787	0,03719	1,022353	0,04228	0,030143	0,08027	0,856107	0,012925	0,208069	0,083805	0,017607	0,02485	0,004174	0,024743
k__Bacteria;p__Chloroflexi;c__Chloroflexi;o__[Roseiflexales];f__;g__;s__	0	0	0,00416	0	0	0	0	0	0	0	0	0	0	0	0	0,00419	0	0	0	0
k__Bacteria;p__Chloroflexi;c__Ellin6529;o__;f__;g__;s__	1,035429	0,527961	1,035903	1,00515	1,508939	1,04852	0,777124	0,636364	0,753769	0,532725	0,477974	0,4267	0,328947	0,327431	0,818708	0,53216	0,572233	0,691655	0,204533	0,193822

k__Bacteria;p__Chloroflexi;c__Gitt-GS-136;o__;f__;g__;s__	1,472056	0,544856	0,877813	0,805782	1,045596	0,875822	0,532394	0,719008	0,459193	0,870962	0,831073	0,747782	0,181343	0,448063	0,610639	0,662057	0,277313	0,608822	0,17114	0,218566
k__Bacteria;p__Chloroflexi;c__Ktedonobacteria;o__;f__;g__;s__	0,016633	0,004224	0,012481	0,008307	0,008201	0,008224	0	0	0,008664	0,004228	0,012918	0,00845	0,008435	0,012925	0,004523	0,00838	0,013205	0,02485	0,008348	0,012372
k__Bacteria;p__Chloroflexi;c__P2-11E;o__;f__;g__;s__	1,771457	1,440277	2,063485	0,539957	0,610956	1,287007	11,33056	2,975207	10,0979	3,454253	3,617104	2,982678	5,524629	3,804231	6,12448	1,386968	4,529448	3,35059	2,671453	1,183554
k__Bacteria;p__Chloroflexi;c__S085;o__;f__;g__;s__	0,128909	0,130934	0,095686	0,182755	0,143513	0,15625	0,081577	0,070248	0,060648	0,114155	0,142101	0,08872	0,021086	0,068933	0,081418	0,100566	0,12325	0,169807	0,033393	0,020619
k__Bacteria;p__Chloroflexi;c__SHA-26;o__;f__;g__;s__	0,141384	0,071803	0,120647	0,186908	0,291127	0,127467	0,060109	0,045455	0,155952	0,04228	0,051673	0,071821	0,025304	0,086166	0,081418	0,205322	0,101241	0,111824	0,033393	0,012372
k__Bacteria;p__Chloroflexi;c__TK10;Other;Other;Other;Other	0	0	0	0	0,008201	0	0	0	0,004332	0	0,004306	0	0	0	0	0	0	0,004142	0	0
k__Bacteria;p__Chloroflexi;c__TK10;o__;f__;g__;s__	0,012475	0,004224	0,008321	0,008307	0,0041	0,004112	0	0,004132	0,012996	0	0	0,004225	0	0	0,009046	0,00419	0	0,004142	0,004174	0
k__Bacteria;p__Chloroflexi;c__TK10;o__AKYG885;f__;g__;s__	0,033267	0,016895	0,008321	0,004154	0,012301	0,024671	0,025761	0,008264	0,025992	0,004228	0,02153	0,012674	0,012652	0,034466	0,009046	0,00838	0,017607	0,033133	0,029219	0,037115
k__Bacteria;p__Chloroflexi;c__TK10;o__AKYG885;f__5B-12;g__;s__	0	0	0	0	0	0	0	0	0	0	0,004306	0	0	0	0,004523	0	0	0	0	0
k__Bacteria;p__Chloroflexi;c__TK10;o__B07_WMSP1;Other;Other;Other	0,033267	0,016895	0,008321	0,045689	0,045104	0,012336	0,021468	0	0,06498	0,016912	0,017224	0,004225	0,033738	0,012925	0,01357	0,037712	0,008804	0,082833	0,004174	0
k__Bacteria;p__Chloroflexi;c__TK10;o__B07_WMSP1;f__;g__;s__	0,286926	0,122487	0,149769	0,299053	0,241922	0,226151	0,253317	0,066116	0,246924	0,194487	0,142101	0,063371	0,113866	0,090474	0,16736	0,1718	0,09684	0,140816	0,087657	0,057734
k__Bacteria;p__Chloroflexi;c__TK10;o__B07_WMSP1;f__FFCH4570;g__;s__	0,07485	0,033789	0,041603	0,053996	0,032803	0,069901	0,025761	0,033058	0,030324	0,008456	0,012918	0,004225	0,021086	0,030158	0,004523	0,020951	0,013205	0,041416	0,020871	0,004124
k__Bacteria;p__Chloroflexi;c__TK17;o__;f__;g__;s__	0,033267	0,038013	0,033282	0,020768	0,028703	0,053454	0,034348	0,028926	0,012996	0,04228	0,017224	0,016899	0,012652	0,008617	0,031663	0,041902	0,052822	0,028992	0,029219	0,032991
k__Bacteria;p__Chloroflexi;c__TK17;o__mle1-48;f__;g__;s__	0,02495	0,004224	0	0	0	0,008224	0	0	0	0	0	0,012674	0,016869	0,008617	0	0,00419	0,008804	0,008283	0,016697	0,008248
k__Bacteria;p__Chloroflexi;c__Thermomicrobia;o__;f__;g__;s__	0,0998	0,050684	0,099846	0,161987	0,155814	0,127467	0,07299	0,049587	0,056316	0,097243	0,017224	0,021124	0,025304	0,034466	0,049756	0,058663	0,044018	0,111824	0,054264	0,07423
k__Bacteria;p__Chloroflexi;c__Thermomicrobia;o__AKYG1722;f__;g__;s__	0,578011	0,181619	0,915256	1,711248	1,931278	1,081414	0,58821	0,198347	0,467857	0,114155	0,249752	0,152091	0,383772	0,090474	0,782522	0,188561	0,761511	0,360323	0,350628	0,152584
k__Bacteria;p__Chloroflexi;c__Thermomicrobia;o__JG30-KF-CM45;f__;g__;s__	1,900361	0,751816	1,065025	4,622861	3,062982	2,471217	0,815766	0,599174	0,992029	1,141553	1,197089	1,313899	0,413293	1,012451	1,216754	0,938613	0,801127	1,312901	0,371499	0,5526
k__Bacteria;p__Chloroflexi;c__Thermomicrobia;o__Sphaerobacterales;f__;g__;s__	0	0	0	0	0	0,01233	0	0	0	0,00845	0	0	0	0	0,00904	0	0	0	0	0

						6				6					6					
k__Bacteria;p__Chloroflexi;c__[Thermobacula];o__[Thermobaculales];f__[Thermobaculaceae];g__Thermobaculum;s__	0	0,004224	0,008321	0,012461	0,0041	0,008224	0,008587	0,004132	0,008664	0,012684	0,004306	0,012674	0,004217	0	0	0,020951	0,004402	0,02485	0,004174	0,008248
k__Bacteria;p__Cyanobacteria;c__4C0d-2;o__MLE1-12;f__;g__;s__	0	0	0	0	0	0,004112	0	0	0	0	0,008612	0	0	0	0	0	0	0	0	0
k__Bacteria;p__Cyanobacteria;c__Chloroplast;o__Streptophyta;f__;g__;s__	0	0	0	0	0	0	0	0,020661	0	0,016912	0	0,004225	0	0	0	0,00419	0	0,020708	0,025045	0,012372
k__Bacteria;p__Cyanobacteria;c__ML635J-21;o__;f__;g__;s__	0,162176	0,109816	0,291218	0,008307	0	0,008224	0,390709	0,177686	0,524173	0,325554	2,157344	1,65188	0,168691	1,856878	0,113081	0,255604	0,145259	0,207082	0,029219	0,119593
k__Bacteria;p__Cyanobacteria;c__Synechococcophycideae;o__Pseudanabaenales;f__Pseudanabaenaceae;g__;s__	0,008317	0	0	0	0	0	0	0	0	0	0	0	0	0	0	0	0	0	0	0
k__Bacteria;p__Cyanobacteria;c__Synechococcophycideae;o__Pseudanabaenales;f__Pseudanabaenaceae;g__Leptolyngbya;s__	0,008317	0	0	0	0	0	0	0	0	0	0	0	0	0	0	0	0	0	0	0
k__Bacteria;p__Elusimicrobia;c__Elusimicrobia;o__Elusimicrobiales;f__;g__;s__	0	0	0	0	0	0	0,004294	0	0,004332	0	0	0	0	0	0	0,00419	0	0	0	0
k__Bacteria;p__Elusimicrobia;c__Elusimicrobia;o__IIb;f__;g__;s__	0	0	0,008321	0	0,0041	0,008224	0	0,012397	0	0	0	0	0	0	0,01357	0,00838	0	0	0	0
k__Bacteria;p__FBP;c__;o__;f__;g__;s__	0,062375	0,139382	0,066564	0,157834	0,057405	0,094572	0,051522	0,082645	0,112632	0,088787	0,025836	0,059147	0,050607	0,038775	0,036186	0,050283	0,154063	0,053841	0,029219	0,049487
k__Bacteria;p__Fibrobacteres;c__Fibrobacteria;o__258ds10;f__;g__;s__	0	0,084474	0,024962	0	0,016402	0,028783	0,437937	0,07438	0,480853	1,150008	0	0,228137	0	0	0	0,00838	0,004402	0,012425	0	0
k__Bacteria;p__Firmicutes;c__Bacilli;o__Bacillales;Other;Other;Other	0	0,004224	0	0	0,0041	0	0	0	0	0	0,004306	0,021124	0	0,004308	0,004523	0	0,004402	0	0,012522	0,004124
k__Bacteria;p__Firmicutes;c__Bacilli;o__Bacillales;f__;g__;s__	0,004158	0,008447	0	0,012461	0,0041	0,160362	0	0,016529	0	0	0	0,004225	0,004217	0,008617	0	0,00419	0	0	0,037567	0
k__Bacteria;p__Firmicutes;c__Bacilli;o__Bacillales;f__Alicyclobacillaceae;g__Alicyclobacillus;s__	0,029108	0	0,012481	0,004154	0,012301	0,008224	0,055816	0	0,012996	0	0	0	0	0	0	0,016761	0,105643	0,020708	0	0
k__Bacteria;p__Firmicutes;c__Bacilli;o__Bacillales;f__Bacillaceae;Other;Other	0,07485	0,084474	0,00416	0,012461	0,024602	0,020559	0,038642	0,024793	0,056316	0,122611	0,12057	0,207013	0,054825	0,137866	0,027139	0,054473	0,022009	0,078691	0,091831	0,032991
k__Bacteria;p__Firmicutes;c__Bacilli;o__Bacillales;f__Bacillaceae;g__;s__	0	0,008447	0	0	0	0	0	0	0	0	0	0	0	0,004308	0,004523	0	0	0,004142	0	0
k__Bacteria;p__Firmicutes;c__Bacilli;o__Bacillales;f__Bacillaceae;g__Bacillus;Other	0	0,012671	0,00416	0,004154	0,0041	0	0,008587	0	0	0,008456	0,004306	0	0	0	0,004523	0,00838	0	0,008283	0,108528	0
k__Bacteria;p__Firmicutes;c__Bacilli;o__Bacillales;f__Bacillaceae;g__Bacillus;s__	0,582169	1,643014	0,016641	0,066456	0,024602	0,185033	0,077283	0,057851	0,056316	0,803315	0,744951	1,981411	0,156039	1,046917	0,076895	0,284936	0,510608	0,132533	6,49497	0,185575

k__Bacteria;p__Firmicutes;c__Bacilli;o__Bacillales;f__Bacillaceae;g__Bacillus;s__firmus	0,004158	0	0	0	0,0041	0,172697	0	0	0	0	0	0	0	0,004308	0	0	0	0	0	0
k__Bacteria;p__Firmicutes;c__Bacilli;o__Bacillales;f__Bacillaceae;g__Geobacillus;s__	0	0	0	0	0	0	0	0	0	0	0	0	0	0	0	0	0,004402	0	0,004174	0
k__Bacteria;p__Firmicutes;c__Bacilli;o__Bacillales;f__Paenibacillaceae;g__;s__	0	0	0	0,008307	0	0	0,090164	0	0,060648	0,025368	0,172243	0,016899	0,198212	0,021542	0	0,108946	0,013205	0,041416	0,125224	0,012372
k__Bacteria;p__Firmicutes;c__Bacilli;o__Bacillales;f__Paenibacillaceae;g__Brevibacillus;Other	0	0	0	0	0	0,004112	0	0	0	0	0	0	0	0	0	0	0	0	0,025045	0
k__Bacteria;p__Firmicutes;c__Bacilli;o__Bacillales;f__Paenibacillaceae;g__Brevibacillus;s__	0	0,016895	0,024962	0,004154	0	0,037007	0	0	0	0	0,008612	0	0	0	0	0,00419	0,013205	0,057983	0,271319	0
k__Bacteria;p__Firmicutes;c__Bacilli;o__Bacillales;f__Paenibacillaceae;g__Cohnella;s__	0,016633	0,046461	0	0	0,0041	0,049342	0,004294	0	0,004332	0,008456	0	0	0	0	0,009046	0,00838	0	0,004142	0,062612	0,016496
k__Bacteria;p__Firmicutes;c__Bacilli;o__Bacillales;f__Paenibacillaceae;g__Paenibacillus;s__	0,141384	0,19429	0,033282	0,037382	0,028703	0,106908	0,034348	0,173554	0,02166	0,676476	0,150713	0,642163	0,037955	0,28004	0,040709	0,343599	0,118848	0,165666	2,429353	0,169079
k__Bacteria;p__Firmicutes;c__Bacilli;o__Bacillales;f__Paenibacillaceae;g__Paenibacillus;s__lautus	0	0	0	0	0	0,012336	0	0	0	0	0	0	0	0	0	0	0,008804	0	0	0
k__Bacteria;p__Firmicutes;c__Bacilli;o__Bacillales;f__Paenibacillaceae;g__Paenibacillus;s__macerans	0	0	0,00416	0	0	0,004112	0	0	0	0	0	0	0	0	0	0	0	0	0,016697	0
k__Bacteria;p__Firmicutes;c__Bacilli;o__Bacillales;f__Planococcaceae;g__Planomicrobium;s__	0	0	0,00416	0	0	0	0	0	0	0	0,004306	0	0	0	0	0	0	0	0	0
k__Bacteria;p__Firmicutes;c__Bacilli;o__Bacillales;f__Planococcaceae;g__Sporosarcina;s__	0	0,004224	0	0	0	0	0	0	0	0	0	0	0	0	0	0,00419	0	0	0	0
k__Bacteria;p__Firmicutes;c__Bacilli;o__Bacillales;f__Staphylococcaceae;g__Macrococcus;s__caseolyticus	0	0	0	0	0	0	0	0	0	0	0	0	0	0	0	0	0	0	0	0,012372
k__Bacteria;p__Firmicutes;c__Bacilli;o__Bacillales;f__Staphylococcaceae;g__Staphylococcus;Other	0	0	0	0	0	0	0	0,008264	0	0	0	0	0	0,004308	0	0	0	0	0,004174	0,004124
k__Bacteria;p__Firmicutes;c__Bacilli;o__Bacillales;f__Staphylococcaceae;g__Staphylococcus;s__	0	0,004224	0,00416	0,016614	0,008201	0,012336	0,008587	0,309917	0,051984	0,033824	0,004306	0,016899	0,008435	0,017233	0	0,037712	0,004402	0,095258	0,146095	0,230937
k__Bacteria;p__Firmicutes;c__Bacilli;o__Bacillales;f__Staphylococcaceae;g__Staphylococcus;s__aureus	0	0	0	0	0	0	0	0	0	0,004228	0	0	0	0	0	0,00419	0	0	0	0
k__Bacteria;p__Firmicutes;c__Bacilli;o__Bacillales;f__[Exiguobacteraceae];g__Exiguobacterium;s__	0	0	0	0	0,0041	0	0	0	0	0	0	0	0	0	0	0	0	0	0,012522	0
k__Bacteria;p__Firmicutes;c__Bacilli;o__Gemellales;f__Gemellaceae;g__;s__	0	0	0	0	0	0	0	0	0	0	0	0	0	0	0	0	0	0	0,020871	0,004124
k__Bacteria;p__Firmicutes;c__Bacilli;o__Lactobacillales;f__Aerococcaceae;g__Aerococcus;s__	0	0	0	0	0,0041	0	0	0	0	0	0	0	0	0	0	0	0	0	0	0,02061

																				9
k__Bacteria;p__Firmicutes;c__Bacilli;o__Lactobacillales;f__Carnobacteriaceae;g__Granulicatella;s__	0	0	0	0	0	0	0	0	0,004332	0,004228	0	0	0	0	0	0,00419	0	0,004142	0,016697	0,004124
k__Bacteria;p__Firmicutes;c__Bacilli;o__Lactobacillales;f__Enterococcaceae;g__Enterococcus;Other	0,004158	0	0	0	0	0	0,004294	0	0	0	0	0	0	0	0	0	0	0,012425	0	0
k__Bacteria;p__Firmicutes;c__Bacilli;o__Lactobacillales;f__Lactobacillaceae;g__Lactobacillus;s__	0,004158	0	0	0,008307	0	0	0	0,004132	0,004332	0,008456	0	0,00845	0	0	0	0,00838	0,004402	0	0,008348	0,008248
k__Bacteria;p__Firmicutes;c__Bacilli;o__Lactobacillales;f__Streptococcaceae;g__Lactococcus;s__	0	0	0	0	0	0	0	0,004132	0	0	0	0	0,004217	0	0	0,00419	0	0,008283	0,004174	0
k__Bacteria;p__Firmicutes;c__Bacilli;o__Lactobacillales;f__Streptococcaceae;g__Streptococcus;s__	0,004158	0	0	0,016614	0,0041	0,008224	0	0,004132	0,008664	0,029596	0,004306	0	0,008435	0,004308	0,004523	0,00419	0,013205	0,028992	0,041741	0,045363
k__Bacteria;p__Firmicutes;c__Clostridia;o__Clostridiales;f__;g__;s__	0	0	0	0	0	0	0	0,004132	0	0,004228	0	0	0	0	0	0,00838	0	0	0,016697	0
k__Bacteria;p__Firmicutes;c__Clostridia;o__Clostridiales;f__Christensenellaceae;g__;s__	0	0	0	0	0	0	0	0	0	0	0	0	0	0,004308	0	0	0	0	0,05009	0
k__Bacteria;p__Firmicutes;c__Clostridia;o__Clostridiales;f__Lachnospiraceae;g__;s__	0	0	0	0	0	0	0	0,004132	0	0	0	0	0	0	0	0,00838	0	0	0,004174	0
k__Bacteria;p__Firmicutes;c__Clostridia;o__Clostridiales;f__Lachnospiraceae;g__Blautia;Other	0	0	0	0	0	0	0	0	0	0	0	0	0	0	0	0,012571	0	0	0	0
k__Bacteria;p__Firmicutes;c__Clostridia;o__Clostridiales;f__Lachnospiraceae;g__Blautia;s__	0	0	0	0	0	0	0	0	0	0	0	0	0	0	0	0,012571	0	0,004142	0	0
k__Bacteria;p__Firmicutes;c__Clostridia;o__Clostridiales;f__Lachnospiraceae;g__Coprococcus;s__	0	0	0	0	0	0	0	0	0	0	0	0	0	0	0	0	0	0	0,029219	0
k__Bacteria;p__Firmicutes;c__Clostridia;o__Clostridiales;f__Lachnospiraceae;g__Lachnospira;s__	0	0	0	0	0	0	0	0	0	0	0	0	0	0	0	0	0	0	0	0,008248
k__Bacteria;p__Firmicutes;c__Clostridia;o__Clostridiales;f__Lachnospiraceae;g__Roseburia;s__	0	0	0	0	0	0	0	0,008264	0	0	0	0	0	0	0	0	0	0	0	0
k__Bacteria;p__Firmicutes;c__Clostridia;o__Clostridiales;f__Lachnospiraceae;g__Roseburia;s__faecis	0	0	0	0	0	0,004112	0	0	0	0	0	0	0	0	0	0,00419	0	0	0,004174	0
k__Bacteria;p__Firmicutes;c__Clostridia;o__Clostridiales;f__Ruminococcaceae;Other;Other	0	0	0	0	0	0	0	0	0	0	0	0	0	0	0	0	0	0	0,008348	0
k__Bacteria;p__Firmicutes;c__Clostridia;o__Clostridiales;f__Ruminococcaceae;g__;s__	0	0	0	0	0,0041	0	0	0,024793	0	0	0	0	0,004217	0	0	0,025141	0	0	0,05009	0
k__Bacteria;p__Firmicutes;c__Clostridia;o__Clostridiales;f__Ruminococcaceae;g__Faecalibacterium;s__prausnitzii	0	0	0	0	0	0	0	0,004132	0	0	0	0	0	0	0	0	0,004402	0	0	0

k__Bacteria;p__Firmicutes;c__Clostridia;o__Clostridiales;f__Ruminococcaceae;g__Ruminococcus;s__	0	0	0	0	0	0	0	0	0	0	0	0	0	0	0	0,00838	0	0	0,025045	0
k__Bacteria;p__Firmicutes;c__Clostridia;o__Clostridiales;f__Ruminococcaceae;g__Ruminococcus;s__bromii	0	0	0	0	0	0	0	0,028926	0	0	0	0	0	0	0	0,016761	0	0	0	0
k__Bacteria;p__Firmicutes;c__Clostridia;o__Clostridiales;f__Symbiobacteriaceae;g__Symbiobacterium;s__	0	0,012671	0	0	0	0,008224	0,098751	0	0	0	0	0	0	0	0	0	0	0	0	0
k__Bacteria;p__Firmicutes;c__Clostridia;o__Clostridiales;f__Veillonellaceae;g__Dialister;s__	0	0	0	0	0	0	0	0,123967	0	0	0	0	0	0	0	0	0	0	0	0,024743
k__Bacteria;p__Firmicutes;c__Clostridia;o__Clostridiales;f__Veillonellaceae;g__Selenomonas;s__	0	0	0	0	0	0	0	0	0	0	0	0	0	0	0	0	0	0,004142	0	0,004124
k__Bacteria;p__Firmicutes;c__Clostridia;o__Clostridiales;f__Veillonellaceae;g__Veillonella;s__dispar	0	0	0	0	0	0,004112	0	0	0	0	0	0	0	0	0	0	0	0,004142	0	0
k__Bacteria;p__Firmicutes;c__Clostridia;o__Clostridiales;f__[Tissierellaceae];g__Anaerococcus;s__	0	0	0	0	0	0,004112	0	0,008264	0,004332	0,004228	0	0	0	0	0	0	0	0,004142	0,016697	0,008248
k__Bacteria;p__Firmicutes;c__Clostridia;o__Clostridiales;f__[Tissierellaceae];g__Finegoldia;s__	0	0	0	0	0,0041	0	0	0,008264	0,004332	0	0	0	0	0	0	0	0	0,008283	0,016697	0,020619
k__Bacteria;p__Firmicutes;c__Clostridia;o__Clostridiales;f__[Tissierellaceae];g__Peptoniphilus;s__	0	0	0	0	0	0	0	0	0	0	0	0	0	0	0	0	0	0	0	0,012372
k__Bacteria;p__Fusobacteria;c__Fusobacteriia;o__Fusobacteriales;f__Fusobacteriaceae;g__Fusobacterium;s__	0	0	0	0	0	0	0,012881	0	0,012996	0,004228	0	0	0	0	0,004523	0	0	0,008283	0	0
k__Bacteria;p__Fusobacteria;c__Fusobacteriia;o__Fusobacteriales;f__Leptotrichiaceae;g__Leptotrichia;s__	0	0	0	0	0	0,008224	0	0	0	0	0	0	0,004217	0	0	0	0	0	0,008348	0,012372
k__Bacteria;p__GAL15;c__;o__;f__;g__;s__	0,927312	0,549079	0,15809	0,137066	0,299328	0,246711	0,373535	0,227273	0,320568	0,194487	0,249752	0,232362	0,18556	0,249882	0,262348	0,238843	0,316929	0,393456	0,162792	0,12784
k__Bacteria;p__GN02;c__GKS2-174;o__;f__;g__;s__	0,020792	0,008447	0,020801	0,049842	0,041004	0	0,004294	0	0,004332	0,012684	0	0,004225	0	0	0	0	0,004402	0,004142	0	0
k__Bacteria;p__Gemmatimonadetes;c__;o__;f__;g__;s__	0,012475	0	0,00416	0,004154	0	0,008224	0,008587	0	0,008664	0	0,004306	0,00845	0	0,004308	0,004523	0	0,008804	0,004142	0	0
k__Bacteria;p__Gemmatimonadetes;c__Gemm-1;o__;f__;g__;s__	0	0	0	0,008307	0,008201	0,012336	0	0	0	0,004228	0	0,004225	0	0	0	0	0	0	0	0,012372
k__Bacteria;p__Gemmatimonadetes;c__Gemm-3;o__;f__;g__;s__	0,989687	0,625106	0,262096	0,265825	0,360833	0,678454	1,59289	0,483471	0,454861	1,784204	0,525341	1,001267	2,239372	0,624704	0,294011	2,271108	1,430584	0,468006	2,391785	0,911378
k__Bacteria;p__Gemmatimonadetes;c__Gemm-5;o__;f__;g__;s__	0,02495	0,008447	0,016641	0	0	0,008224	0,012881	0,008264	0,025992	0,008456	0,008612	0	0,012652	0	0,004523	0,012571	0,013205	0,037275	0	0
k__Bacteria;p__Gemmatimonadetes;c__Gemmatimonadetes;Other;Other;Other;Other	0,05821	0,00844	0,00832	0,00830	0,02050	0,02467	0,01717	0	0,06064	0,01691	0,01722	0,05492	0,03373	0,02585	0,01809	0,00419	0,03081	0,02070	0	0,00824

	7	7	1	7	2	1	4		8	2	4	2	8		3		3	8		8
k__Bacteria;p__Gemmatimonadetes;c__Gemmatimonadetes;o__;f__;g__;s__	3,82984	2,052712	3,839913	4,024755	5,966049	5,748355	3,194367	1,272727	2,18333	2,490276	1,92051	2,332066	1,045884	1,762096	0,89108	1,650953	1,553834	1,847173	0,788913	0,70106
k__Bacteria;p__Gemmatimonadetes;c__Gemmatimonadetes;o__C114;f__;g__;s__	0,045742	0,046461	0	0,004154	0,0041	0,004112	0,004294	0,004132	0	0,02114	0,004306	0	0	0	0	0,00838	0,013205	0,008283	0,008348	0
k__Bacteria;p__Gemmatimonadetes;c__Gemmatimonadetes;o__Ellin5290;f__;g__;s__	0,790086	0,371684	0,316179	0,681176	1,061998	2,039474	0,42935	0,318182	0,337896	0,553864	0,383241	0,806929	0,303644	0,262806	0,230686	0,222083	0,576635	0,62953	0,375673	0,136088
k__Bacteria;p__Gemmatimonadetes;c__Gemmatimonadetes;o__Gemmatimonadales;Other;Other;Other	0	0	0,016641	0,008307	0	0,024671	0,004294	0	0	0,008456	0,004306	0,004225	0,008435	0,012925	0,004523	0,00419	0,004402	0	0,016697	0
k__Bacteria;p__Gemmatimonadetes;c__Gemmatimonadetes;o__Gemmatimonadales;f__;g__;s__	0,90652	0,388579	0,753006	0,976076	1,053797	0,472862	0,618265	0,371901	0,701785	0,655336	0,327262	0,304183	0,387989	0,43083	0,379953	0,280746	0,422572	0,803479	0,488375	0,416512
k__Bacteria;p__Gemmatimonadetes;c__Gemmatimonadetes;o__Gemmatimonadales;f__A1-B1;g__;s__	0,012475	0,004224	0,00416	0,016614	0,012301	0,024671	0	0	0	0	0	0,00845	0	0	0	0,00838	0,013205	0,004142	0,004174	0,008248
k__Bacteria;p__Gemmatimonadetes;c__Gemmatimonadetes;o__Gemmatimonadales;f__Ellin5301;g__;s__	0,008317	0	0,041603	0,066456	0,114811	0,189145	0,030055	0,004132	0,02166	0,033824	0,012918	0,00845	0,004217	0	0,004523	0,020951	0,039616	0,016567	0,004174	0,004124
k__Bacteria;p__Gemmatimonadetes;c__Gemmatimonadetes;o__Gemmatimonadales;f__Gemmatimonadaceae;g__Gemmatimonas;s__	0	0	0,00416	0	0	0	0	0	0,004332	0	0,004306	0	0	0	0	0	0	0	0	0
k__Bacteria;p__Gemmatimonadetes;c__Gemmatimonadetes;o__N1423WL;f__;g__;s__	0	0	0	0	0	0	0	0	0,004332	0	0	0	0	0,004308	0	0	0	0,008283	0	0,008248
k__Bacteria;p__MVP-21;c__;o__;f__;g__;s__	0,212076	0,050684	0,16225	0,261671	0,332131	0,217928	0,107338	0,082645	0,08664	0,025368	0,068897	0,084495	0,059042	0,068933	0,126651	0,121517	0,114447	0,132533	0,033393	0,028867
k__Bacteria;p__NC10;c__12-24;Other;Other;Other;Other	0	0	0	0	0	0	0	0	0	0	0	0	0	0	0	0	0,004402	0,008283	0,025045	0
k__Bacteria;p__Nitrospirae;c__Nitrospira;o__Nitrospirales;f__;g__;s__	0,004158	0	0	0	0	0	0	0	0	0	0	0	0	0	0	0	0	0	0,016697	0
k__Bacteria;p__Nitrospirae;c__Nitrospira;o__Nitrospirales;f__0319-6A21;g__;s__	0,020792	0,008447	0	0,004154	0,0041	0,012336	0,021468	0,004132	0,025992	0,016912	0,02153	0	0,025304	0,038775	0,009046	0,020951	0,008804	0,02485	0,012522	0,004124
k__Bacteria;p__Nitrospirae;c__Nitrospira;o__Nitrospirales;f__Nitrospiraceae;g__;s__	0,203759	0,236526	0,091526	0,228443	0,172216	1,134868	0,231849	0,161157	0,116964	0,084559	0,086122	0,063371	0,04639	0,064625	0,054279	0,079614	0,114447	0,103541	0,025045	0,05361
k__Bacteria;p__Nitrospirae;c__Nitrospira;o__Nitrospirales;f__Nitrospiraceae;g__Nitrospira;s__	0,062375	0,025342	0,099846	0,137066	0,077907	0,349507	0,107338	0,041322	0,077976	0,046508	0,073203	0,059147	0,050607	0,038775	0,049756	0,121517	0,052822	0,070408	0	0,020619
k__Bacteria;p__OD1;c__;o__;f__;g__;s__	0,569694	0,228079	0,357782	0,731018	0,578153	0,941612	0,309132	0,053719	0,28158	0,274818	0,193773	0,202788	0,118084	0,103399	0,16736	0,159229	0,211286	0,219507	0,033393	0,041239
k__Bacteria;p__OD1;c__SM2F11;o__;f__;g__;s__	0,066534	0,025342	0,116487	0,020768	0,057405	0,032895	0,07299	0,03719	0,077976	0,046508	0,025836	0,050697	0,054825	0,038775	0,162837	0,075424	0,04842	0,053841	0,016697	0,024743

k__Bacteria;p__OD1;c__ZB2;o__;f__;g__;s__	0,091484	0,059132	0,045763	0,07061	0,082008	0,037007	0,07299	0,061983	0,06498	0,317098	0,02153	0,050697	0,134953	0,034466	0,027139	0,071234	0,088036	0,033133	0,033393	0,004124
k__Bacteria;p__OP11;c__OP11-4;o__;f__;g__;s__	0,004158	0	0	0	0	0,004112	0	0	0	0	0	0	0	0	0,01357	0,00419	0	0	0	0,004124
k__Bacteria;p__Planctomycetes;c__BD7-11;o__;f__;g__;s__	0	0	0,049923	0,004154	0,0041	0	0	0,024793	0,017328	0,016912	0,004306	0,00845	0	0	0	0,00419	0	0,016567	0,004174	0,008248
k__Bacteria;p__Planctomycetes;c__Phycisphaerae;o__Phycisphaerales;f__Phycisphaeraceae;g__;s__	0	0	0	0	0	0	0	0	0	0	0,004306	0,004225	0	0	0	0	0,004402	0	0	0
k__Bacteria;p__Planctomycetes;c__Phycisphaerae;o__WD2101;f__;g__;s__	1,156021	0,477277	1,851313	1,636484	1,176808	0,986842	0,858701	0,607438	0,836077	0,951294	0,409077	0,473173	0,290992	0,379131	0,393523	0,377121	0,576635	0,625388	0,212881	0,296919
k__Bacteria;p__Planctomycetes;c__Pla4;o__;f__;g__;s__	0,070692	0,016895	0,029122	0,029075	0,041004	0,024671	0,042935	0	0,060648	0,016912	0,017224	0,00845	0,012652	0,021542	0,018093	0,020951	0,022009	0,033133	0	0,012372
k__Bacteria;p__Planctomycetes;c__Planctomycetia;o__Gemmatales;f__Gemmataceae;g__;s__	0,108117	0,021118	0,016641	0,008307	0,028703	0,041118	0,012881	0,008264	0,06498	0,050736	0,025836	0,012674	0,016869	0,012925	0,018093	0,029332	0,070429	0,086975	0,012522	0,008248
k__Bacteria;p__Planctomycetes;c__Planctomycetia;o__Gemmatales;f__Gemmataceae;g__Gemmata;s__	0,062375	0,016895	0,083205	0,041535	0,045104	0,012336	0,030055	0,028926	0,012996	0,06342	0,012918	0,012674	0	0,004308	0,027139	0,016761	0,030813	0,041416	0,075135	0,032991
k__Bacteria;p__Planctomycetes;c__Planctomycetia;o__Gemmatales;f__Isosphaeraceae;g__;s__	0,286926	0,021118	0,054083	0,045689	0,061506	0,020559	0,060109	0,14876	0,099636	0,135295	0,030143	0,029573	0,042173	0,047391	0,031663	0,058663	0,035214	0,236074	0,033393	0,049487
k__Bacteria;p__Planctomycetes;c__Planctomycetia;o__Pirellulales;f__Pirellulaceae;g__;s__	1,480373	0,451934	0,761326	0,365509	0,401837	0,719572	0,927397	0,752066	0,554497	0,858278	0,34018	0,316857	0,253036	0,245573	0,37543	0,406453	0,933181	0,828329	0,204533	0,144336
k__Bacteria;p__Planctomycetes;c__Planctomycetia;o__Pirellulales;f__Pirellulaceae;g__A17;s__	0,0499	0,012671	0,008321	0,020768	0,041004	0,020559	0,021468	0	0,004332	0,004228	0,004306	0,025349	0,004217	0,017233	0,027139	0,00419	0,026411	0,078691	0,045916	0,012372
k__Bacteria;p__Planctomycetes;c__Planctomycetia;o__Planctomycetales;f__Planctomycetaceae;g__Planctomyces;s__	0	0,004224	0,012481	0	0	0,086349	0	0	0,004332	0	0	0,004225	0	0	0	0,00419	0	0,008283	0	0
k__Bacteria;p__Planctomycetes;c__vadinHA49;o__DH61;f__;g__;s__	0,087325	0	0,058244	0,128759	0,200918	0,197368	0,07299	0,045455	0,060648	0,143751	0,017224	0,046472	0	0	0,009046	0,00838	0	0,0497	0,020871	0,012372
k__Bacteria;p__Proteobacteria;c__;o__;f__;g__;s__	0,790086	0,485724	0,569955	0,643795	0,738068	0,530428	0,068696	0,020661	0,727777	1,027397	0,09904	0,054922	0,611505	0,366206	0,262348	0,155039	0,22009	0,248499	0,530116	0,288672
k__Bacteria;p__Proteobacteria;c__Alphaproteobacteria;o__Caulobacterales;f__Caulobacteraceae;g__;s__	0,162176	0,050684	0,220493	0,087224	0,094309	0,152138	0,103044	0,095041	0,125628	0,076104	0,086122	0,063371	0,063259	0,086166	0,072372	0,096375	0,101241	0,173949	0,058438	0,065982
k__Bacteria;p__Proteobacteria;c__Alphaproteobacteria;o__Caulobacterales;f__Caulobacteraceae;g__Asticcacaulis;Other	0	0	0	0	0	0	0	0	0	0	0	0	0	0	0	0	0	0	0	0,008248
k__Bacteria;p__Proteobacteria;c__Alphaproteobacteria;o__Caulobacterales;f__Caulobacteraceae;g__Brevundimonas;s__diminuta	0	0	0	0	0	0	0	0,004132	0	0	0	0	0	0	0	0,00419	0	0	0	0
k__Bacteria;p__Proteobacteria;c__Alphaproteobacteria;o__Caulobacterales;f__Caulobacteraceae;g__Mycoplana;s__	0,00831	0,00422	0,19553	0,01246	0,02460	0,25082	0,01288	0,01652	0	0,05073	0,03014	0,00845	0	0,04308	0	0	0	0	0,02504	0,05773

	7	4	2	1	2	2	1	9		6	3			3					5	4
k__Bacteria;p__Proteobacteria;c__Alphaproteobacteria;o__Caulobacterales;f__Caulobacteraceae;g__Phenylobacterium;s__	0,083167	0,025342	0,012481	0	0,0041	0,024671	0,115925	0,057851	0,350892	0,156435	0,064591	0,038023	0,012652	0,116324	0,230686	0,125707	0,34334	0,103541	0,083483	0,173203
k__Bacteria;p__Proteobacteria;c__Alphaproteobacteria;o__Rhizobiales;Other;Other;Other	0	0	0	0	0	0,004112	0,004294	0	0	0	0	0	0	0,004308	0	0	0	0	0	0
k__Bacteria;p__Proteobacteria;c__Alphaproteobacteria;o__Rhizobiales;f__;g__;s__	0,004158	0,004224	0	0	0	0,098684	0,197501	0,157025	0,2166	0,033824	0,008612	0,00845	0,016869	0,004308	0,045232	0,17599	0,004402	0,004142	0,058438	0,045363
k__Bacteria;p__Proteobacteria;c__Alphaproteobacteria;o__Rhizobiales;f__Aurantimonadaceae;g__;s__	0,004158	0	0	0	0	0	0,004294	0	0	0,004228	0	0	0	0	0	0	0	0	0,016697	0
k__Bacteria;p__Proteobacteria;c__Alphaproteobacteria;o__Rhizobiales;f__Beijerinckiaceae;g__;s__	0,016633	0,008447	0,024962	0,041535	0,016402	0,028783	0,141686	0,03719	0,060648	0,008456	0,030143	0,046472	0,059042	0,086166	0,045232	0,025141	0,052822	0,057983	0,016697	0,008248
k__Bacteria;p__Proteobacteria;c__Alphaproteobacteria;o__Rhizobiales;f__Beijerinckiaceae;g__Beijerinckia;s__	0	0	0	0	0	0	0,008587	0	0	0	0	0	0	0	0	0	0	0	0	0
k__Bacteria;p__Proteobacteria;c__Alphaproteobacteria;o__Rhizobiales;f__Beijerinckiaceae;g__Chelatococcus;Other	0,008317	0	0,008321	0	0	0	0	0,004132	0,004332	0	0	0,004225	0	0	0,004523	0,016761	0	0,008283	0,004174	0,012372
k__Bacteria;p__Proteobacteria;c__Alphaproteobacteria;o__Rhizobiales;f__Bradyrhizobiaceae;g__;s__	0,091484	0,033789	0,041603	0,037382	0,036903	0,037007	0,038642	0,057851	0,051984	0,033824	0,034449	0,029573	0,113866	0,081858	0,031663	0,037712	0,030813	0,178091	0,05009	0,346406
k__Bacteria;p__Proteobacteria;c__Alphaproteobacteria;o__Rhizobiales;f__Bradyrhizobiaceae;g__Balneimonas;s__	0,020792	0,012671	0,00416	0,037382	0	0,020559	0,047229	0,004132	0,017328	0,016912	0	0,004225	0,004217	0,034466	0,01357	0,029332	0,008804	0,02485	0,020871	0,045363
k__Bacteria;p__Proteobacteria;c__Alphaproteobacteria;o__Rhizobiales;f__Hyphomicrobiaceae;g__;s__	0,40336	0,380132	0,029122	0,062303	0,073807	0,082237	0,326306	0,049587	0,17328	0,139523	0,172243	0,130967	0,113866	0,172332	0,149267	0,205322	0,215688	0,364465	0,075135	0,061858
k__Bacteria;p__Proteobacteria;c__Alphaproteobacteria;o__Rhizobiales;f__Hyphomicrobiaceae;g__Devosia;s__	0,054059	0,181619	0,041603	0,004154	0,049205	0,20148	0,240436	0,115702	0,004332	0,04228	0,043061	0,16899	0,067476	0,021542	0	0,125707	0,026411	0	0,020871	0,090725
k__Bacteria;p__Proteobacteria;c__Alphaproteobacteria;o__Rhizobiales;f__Hyphomicrobiaceae;g__Hyphomicrobium;Other	0	0	0	0	0	0	0	0	0	0	0,004306	0	0	0	0	0	0	0	0,012522	0
k__Bacteria;p__Proteobacteria;c__Alphaproteobacteria;o__Rhizobiales;f__Hyphomicrobiaceae;g__Pedomicrobium;s__	0,012475	0,008447	0	0	0	0	0	0	0,008664	0	0	0	0	0	0	0,00419	0	0,004142	0	0
k__Bacteria;p__Proteobacteria;c__Alphaproteobacteria;o__Rhizobiales;f__Hyphomicrobiaceae;g__Rhodoplanes;s__	0,004158	0	0	0,004154	0	0,061678	0,064403	0	0,004332	0,135295	0	0,004225	0	0,008617	0	0	0	0,008283	0,004174	0
k__Bacteria;p__Proteobacteria;c__Alphaproteobacteria;o__Rhizobiales;f__Methylobacteriaceae;g__;s__	0,016633	0,012671	0,00416	0,020768	0	0,012336	0,034348	0,008264	0,008664	0,012684	0	0	0,04639	0,004308	0,004523	0,00419	0,013205	0,016567	0,037567	0,024743
k__Bacteria;p__Proteobacteria;c__Alphaproteobacteria;o__Rhizobiales;f__Methylobacteriaceae;g__Methylobacterium;s__	0,07485	0,050684	0,033282	0,012461	0,032803	0,020559	0,030055	0,107438	0,06498	0,054964	0,055979	0,071821	0,18556	0,155099	0,049756	0,079614	0,044018	0,194657	0,129399	0,40414
k__Bacteria;p__Proteobacteria;c__Alphaproteobacteria;o__Rhizobiales;f__Methylobacteriaceae;g__Methylobacterium;s__adhaesivum	0,016633	0,004224	0,029122	0	0,012301	0	0,051522	0,004132	0,004332	0,012684	0,004306	0,012674	0,004217	0,004308	0	0	0,004402	0,012425	0,033393	0,012372

k__Bacteria;p__Proteobacteria;c__Alphaproteobacteria;o__Rhizobiales;f__Methylocystaceae;g__;s__	0	0	0	0	0	0	0	0	0	0	0	0	0	0,008617	0	0	0	0	0	0,008248
k__Bacteria;p__Proteobacteria;c__Alphaproteobacteria;o__Rhizobiales;f__Phyllobacteriaceae;Other;Other	0	0,004224	0,008321	0	0	0,016447	0,004294	0	0	0	0	0	0	0	0	0	0	0	0	0,008248
k__Bacteria;p__Proteobacteria;c__Alphaproteobacteria;o__Rhizobiales;f__Phyllobacteriaceae;g__;s__	0	0	0	0	0	0,008224	0	0	0	0	0	0,004225	0	0	0	0	0	0	0,154443	0,049487
k__Bacteria;p__Proteobacteria;c__Alphaproteobacteria;o__Rhizobiales;f__Rhizobiaceae;Other;Other	0	0	0	0	0	0,004112	0	0,008264	0	0	0	0	0	0	0	0	0	0	0,025045	0
k__Bacteria;p__Proteobacteria;c__Alphaproteobacteria;o__Rhizobiales;f__Rhizobiaceae;g__;s__	0	0,004224	0	0	0	0	0,030055	0	0,004332	0,004228	0	0,004225	0,004217	0,004308	0,009046	0,00419	0,013205	0,004142	0	0,004124
k__Bacteria;p__Proteobacteria;c__Alphaproteobacteria;o__Rhizobiales;f__Rhizobiaceae;g__Agrobacterium;s__	0,166334	0	0,00416	2,699784	1,213712	6,846217	0,012881	1,219008	0	0,012684	0	0,00845	0,004217	0	0	0,00838	0	0	0,592729	0,045363
k__Bacteria;p__Proteobacteria;c__Alphaproteobacteria;o__Rhizobiales;f__Rhizobiaceae;g__Rhizobium;s__	0	0	0,00416	0	0	0	0	0	0	0	0	0	0	0	0,004523	0	0	0	0	0,004124
k__Bacteria;p__Proteobacteria;c__Alphaproteobacteria;o__Rhizobiales;f__Rhodobiaceae;g__Afifella;s__	0	0,004224	0	0	0	0,004112	0	0,008264	0	0	0	0,004225	0	0	0	0	0	0	0,016697	0
k__Bacteria;p__Proteobacteria;c__Alphaproteobacteria;o__Rhodobacterales;f__Rhodobacteraceae;g__;s__	0,004158	0	0	0,012461	0,0041	0,020559	0,120218	0,045455	0,012996	0	0	0	0,008435	0,004308	0,01357	0,00419	0,004402	0,016567	0,025045	0,016496
k__Bacteria;p__Proteobacteria;c__Alphaproteobacteria;o__Rhodobacterales;f__Rhodobacteraceae;g__Paracoccus;Other	0	0	0	0	0	0	0	0,016529	0	0	0	0	0	0	0	0	0	0	0	0,004124
k__Bacteria;p__Proteobacteria;c__Alphaproteobacteria;o__Rhodobacterales;f__Rhodobacteraceae;g__Paracoccus;s__	0	0,008447	0	0	0	0	0	0	0	0,004228	0	0	0	0,004308	0	0	0,004402	0	0	0
k__Bacteria;p__Proteobacteria;c__Alphaproteobacteria;o__Rhodobacterales;f__Rhodobacteraceae;g__Paracoccus;s__marcusii	0	0	0	0,004154	0,0041	0,008224	0,012881	0,028926	0	0,012684	0,004306	0,00845	0,012652	0,021542	0	0,037712	0,004402	0	0,058438	0,094849
k__Bacteria;p__Proteobacteria;c__Alphaproteobacteria;o__Rhodobacterales;f__Rhodobacteraceae;g__Rhodobacter;s__	0	0	0	0	0	0	0,004294	0	0	0,016912	0,004306	0,004225	0	0	0,004523	0	0	0,004142	0,029219	0,024743
k__Bacteria;p__Proteobacteria;c__Alphaproteobacteria;o__Rhodobacterales;f__Rhodobacteraceae;g__Rubellimicrobium;s__	0,020792	0,004224	0,008321	0,012461	0	0,004112	0,008587	0,012397	0,025992	0	0,004306	0,00845	0,012652	0,004308	0	0,00419	0,017607	0,004142	0,037567	0
k__Bacteria;p__Proteobacteria;c__Alphaproteobacteria;o__Rhodospirillales;f__;g__;s__	0	0	0	0,004154	0,0041	0	0	0	0,004332	0	0	0	0	0	0	0	0	0	0,012522	0
k__Bacteria;p__Proteobacteria;c__Alphaproteobacteria;o__Rhodospirillales;f__Acetobacteraceae;g__;s__	0,166334	0,071803	0,066564	0,195215	0,139413	0,205592	0,17174	0,136364	0,298908	0,122611	0,094734	0,101394	0,13917	0,090474	0,15379	0,125707	0,118848	0,161524	0,212881	0,086602
k__Bacteria;p__Proteobacteria;c__Alphaproteobacteria;o__Rhodospirillales;f__Acetobacteraceae;g__Roseococcus;s__	0,054059	0,08025	0,033282	0,020768	0,008201	0,069901	0,15886	0,070248	0,103968	0,101471	0,060285	0,050697	0,257254	0,068933	0,031663	0,096375	0,145259	0,0497	0,358976	0,098973
k__Bacteria;p__Proteobacteria;c__Alphaproteobacteria;o__Rhodospirillales;f__Acetobacteraceae;g__Roseomonas;Other	0	0	0	0	0	0	0	0	0	0,00845	0	0	0	0	0	0	0	0	0	0

										6										
k__Bacteria;p__Proteobacteria;c__Alphaproteobacteria;o__Rhodospirillales;f__Acetobacteraceae;g__Roseomonas;s__	0	0	0	0	0	0	0	0	0	0	0	0	0	0	0	0	0	0,004142	0,012522	0
k__Bacteria;p__Proteobacteria;c__Alphaproteobacteria;o__Rhodospirillales;f__Rhodospirillaceae;g__;s__	0	0,008447	0	0,012461	0,0041	0,012336	0,012881	0	0	0,016912	0	0,004225	0,004217	0	0	0	0	0	0	0
k__Bacteria;p__Proteobacteria;c__Alphaproteobacteria;o__Rhodospirillales;f__Rhodospirillaceae;g__Skermanella;s__	0,004158	0,008447	0	0	0	0	0,004294	0	0	0,012684	0	0	0,004217	0,004308	0,004523	0	0	0	0	0,024743
k__Bacteria;p__Proteobacteria;c__Alphaproteobacteria;o__Rickettsiales;f__;g__;s__	0,212076	0,1605	0,041603	0,053996	0,028703	0,016447	0,064403	0,070248	0,047652	0,109927	0,047367	0,164766	0,004217	0,012925	0,054279	0,167609	0,004402	0,016567	0	0,008248
k__Bacteria;p__Proteobacteria;c__Alphaproteobacteria;o__Rickettsiales;f__Rickettsiaceae;g__;s__	0	0	0,00416	0	0,0041	0	0	0	0,004332	0	0	0	0	0,004308	0	0	0	0	0	0
k__Bacteria;p__Proteobacteria;c__Alphaproteobacteria;o__Sphingomonadales;f__Erythrobacteraceae;g__;s__	0,033267	0,008447	0,016641	0,012461	0,024602	0,049342	0,042935	0,045455	0,004332	0,046508	0,017224	0,012674	0,016869	0	0,009046	0,025141	0,017607	0,008283	0,066786	0,037115
k__Bacteria;p__Proteobacteria;c__Alphaproteobacteria;o__Sphingomonadales;f__Sphingomonadaceae;Other;Other	0	0	0	0	0	0	0,004294	0	0	0,004228	0,004306	0,004225	0	0,017233	0,01357	0	0	0	0	0,004124
k__Bacteria;p__Proteobacteria;c__Alphaproteobacteria;o__Sphingomonadales;f__Sphingomonadaceae;g__;s__	0,079009	0,025342	0,029122	0,008307	0,0041	0,057566	1,030441	0,103306	0,1083	0,06342	0,02153	0,054922	0,037955	0,047391	0,036186	0,050283	0,149661	0,053841	0,146095	0,094849
k__Bacteria;p__Proteobacteria;c__Alphaproteobacteria;o__Sphingomonadales;f__Sphingomonadaceae;g__Kaistobacter;s__	0,340985	0,11404	0,81541	0,141219	0,22142	0,847039	0,678374	0,553719	0,38988	1,133097	0,31865	0,544994	0,269906	0,366206	0,262348	0,213702	0,792323	0,356181	0,784739	0,131964
k__Bacteria;p__Proteobacteria;c__Alphaproteobacteria;o__Sphingomonadales;f__Sphingomonadaceae;g__Novosphingobium;Other	0,037425	0,016895	0,00416	0	0	0,004112	0,004294	0,066116	0,030324	0,04228	0,043061	0,033798	0,063259	0,129249	0,01357	0,041902	0,022009	0,07455	0,066786	0,206194
k__Bacteria;p__Proteobacteria;c__Alphaproteobacteria;o__Sphingomonadales;f__Sphingomonadaceae;g__Novosphingobium;s__	0,037425	0,004224	0	0	0,0041	0,004112	0,038642	0,078512	0	0,071876	0,004306	0,016899	0,008435	0,292965	0	0,381312	0,127652	0,016567	0,129399	0,416512
k__Bacteria;p__Proteobacteria;c__Alphaproteobacteria;o__Sphingomonadales;f__Sphingomonadaceae;g__Sphingobium;s__	0,004158	0,008447	0	0	0	0	0,025761	0,07438	0,147288	0,088787	0,107652	0,054922	0,021086	0,921977	0	0,079614	0,052822	0,033133	0,363151	3,806343
k__Bacteria;p__Proteobacteria;c__Alphaproteobacteria;o__Sphingomonadales;f__Sphingomonadaceae;g__Sphingobium;s__xenophagum	0,008317	0	0	0	0,0041	0,004112	0,154566	0,016529	0,004332	0,054964	0,004306	0,00845	0,004217	0,056008	0	0	0,004402	0	0,016697	0,012372
k__Bacteria;p__Proteobacteria;c__Alphaproteobacteria;o__Sphingomonadales;f__Sphingomonadaceae;g__Sphingomonas;Other	0,02495	0,042237	0,008321	0,004154	0,016402	0,004112	0,115925	0,008264	0,004332	0,038052	0,008612	0,00845	0,021086	0,064625	0	0,012571	0,026411	0,012425	0,17114	0
k__Bacteria;p__Proteobacteria;c__Alphaproteobacteria;o__Sphingomonadales;f__Sphingomonadaceae;g__Sphingomonas;s__	0,141384	0,042237	0,112327	0,045689	0,049205	0,164474	0,322013	0,157025	0,212268	0,245222	0,111958	0,122518	0,253036	0,310198	0,547313	0,159229	0,158465	0,393456	0,41324	0,771166
k__Bacteria;p__Proteobacteria;c__Alphaproteobacteria;o__Sphingomonadales;f__Sphingomonadaceae;g__Sphingomonas;s__echinoides	0	0,004224	0	0,004154	0	0	0	0	0	0	0	0	0	0	0	0,00838	0,004402	0	0	0,004124
k__Bacteria;p__Proteobacteria;c__Alphaproteobacteria;o__Sphingomonadales;f__Sphingomonadaceae;g__Sphingomonas;s__wittichii	0,004158	0,025342	0,016641	0,008307	0	0,016447	0,133099	0,004132	0,004332	0,038052	0,008612	0,00845	0,025304	0,0517	0	0,012571	0,030813	0,020708	0,166966	0

k__Bacteria;p__Proteobacteria;c__Alphaproteobacteria;o__Sphingomonadales;f__Sphingomonadaceae;g__Sphingopyxis;s__alaskensis	0	0	0	0	0,008201	0	0	0	0	0	0	0	0	0	0	0	0	0	0	0,05361
k__Bacteria;p__Proteobacteria;c__Betaproteobacteria;Other;Other;Other;Other	0	0,004224	0	0	0	0	0,025761	0	0,017328	0,004228	0,038755	0,004225	0,025304	0,021542	0,01357	0,016761	0,044018	0,012425	0,016697	0,008248
k__Bacteria;p__Proteobacteria;c__Betaproteobacteria;o__;f__;g__;s__	0,499002	0,291434	0,224654	0,191062	0,291127	0,460526	0,798592	0,272727	0,19494	0,388973	0,210998	0,295733	0,198212	0,258498	0,189976	0,280746	0,576635	0,285773	0,400718	0,210318
k__Bacteria;p__Proteobacteria;c__Betaproteobacteria;o__Burkholderiales;Other;Other;Other	0,054059	0,033789	0,020801	0,004154	0	0,008224	0,184621	0,095041	0,038988	0,173347	0,103346	0,059147	0,156039	0,116324	0,067849	0,515399	0,303724	0,041416	0,158618	0,07423
k__Bacteria;p__Proteobacteria;c__Betaproteobacteria;o__Burkholderiales;f__;g__;s__	0,095642	0,042237	0,037442	0,024921	0,016402	0,065789	0,188914	0,020661	0,116964	0,232538	0,103346	0,122518	0,126518	0,150791	0,085942	0,083805	0,228893	0,028992	0,179488	0,041239
k__Bacteria;p__Proteobacteria;c__Betaproteobacteria;o__Burkholderiales;f__Alcaligenaceae;g__;s__	0	0,004224	0	0	0	0	0,008587	0	0,008664	0	0,004306	0	0	0,004308	0	0,00419	0,008804	0,004142	0	0
k__Bacteria;p__Proteobacteria;c__Betaproteobacteria;o__Burkholderiales;f__Alcaligenaceae;g__Achromobacter;s__	0	0	0	0	0	0,020559	0	0,004132	0	0	0	0	0	0	0	0	0	0	0	0,004124
k__Bacteria;p__Proteobacteria;c__Betaproteobacteria;o__Burkholderiales;f__Alcaligenaceae;g__Pigmentiphaga;s__	0,004158	0	0	0	0	0	0	0	0	0,012684	0	0,004225	0	0	0	0	0	0	0	0,004124
k__Bacteria;p__Proteobacteria;c__Betaproteobacteria;o__Burkholderiales;f__Burkholderiaceae;g__;s__	0,004158	0,008447	0,008321	0,004154	0,0041	0,061678	0,300545	0,07438	0,008664	0,012684	0,012918	0,016899	0,004217	0,004308	0,018093	0,00838	0,105643	0,016567	0,162792	0,057734
k__Bacteria;p__Proteobacteria;c__Betaproteobacteria;o__Burkholderiales;f__Burkholderiaceae;g__Lautropia;s__	0,020792	0,021118	0,012481	0,016614	0,020502	0,008224	0,051522	0,033058	0,04332	0,012684	0,038755	0,016899	0,008435	0,021542	0,009046	0,00419	0,008804	0,008283	0,037567	0,024743
k__Bacteria;p__Proteobacteria;c__Betaproteobacteria;o__Burkholderiales;f__Comamonadaceae;Other;Other	0,216234	0,304105	0,216333	0,08307	0,086108	0,119243	0,74707	0,140496	0,567493	0,693387	0,465056	0,481622	0,957321	0,637629	0,384476	0,561492	1,069636	0,182232	0,296364	0,243309
k__Bacteria;p__Proteobacteria;c__Betaproteobacteria;o__Burkholderiales;f__Comamonadaceae;g__;s__	4,441118	4,270147	4,11865	1,702941	1,95178	3,174342	6,204113	2,512397	3,656212	5,695079	2,587952	2,944656	6,043354	3,726681	2,822508	5,573015	8,997271	2,476703	6,76629	2,927956
k__Bacteria;p__Proteobacteria;c__Betaproteobacteria;o__Burkholderiales;f__Comamonadaceae;g__Comamonas;s__	0,016633	0,008447	0	0	0,0041	0	0,021468	0,004132	0,047652	0,033824	0,025836	0,021124	0,054825	0,017233	0,01357	0,020951	0,057223	0,020708	0,004174	0,008248
k__Bacteria;p__Proteobacteria;c__Betaproteobacteria;o__Burkholderiales;f__Comamonadaceae;g__Delftia;s__	1,804724	0,861632	0,112327	0,249211	0,147614	0,485197	1,013267	1,012397	3,8035	3,395062	3,070232	3,016477	4,972166	4,545259	1,623847	2,350723	1,342548	7,972665	3,623158	13,42736
k__Bacteria;p__Proteobacteria;c__Betaproteobacteria;o__Burkholderiales;f__Comamonadaceae;g__Diaphorobacter;s__	0	0	0	0	0	0	0	0	0	0	0	0,00845	0	0	0	0	0,004402	0	0,004174	0
k__Bacteria;p__Proteobacteria;c__Betaproteobacteria;o__Burkholderiales;f__Comamonadaceae;g__Hydrogenophaga;s__	0	0	0	0	0,016402	0	0	0	0	0	0	0	0	0	0,004523	0	0	0	0,271319	0,247433
k__Bacteria;p__Proteobacteria;c__Betaproteobacteria;o__Burkholderiales;f__Comamonadaceae;g__Hylemonella;s__	0	0	0	0	0	0	0,008587	0,004132	0,008664	0,008456	0	0,00845	0,029521	0,004308	0	0,00838	0,008804	0	0	0
k__Bacteria;p__Proteobacteria;c__Betaproteobacteria;o__Burkholderiales;f__Comamonadaceae;g__Leptothrix;s__	0	0	0	0	0	0	0	0	0,00433	0,00422	0	0	0	0,00861	0	0	0	0	0	0

									2	8				7						
k__Bacteria;p__Proteobacteria;c__Betaproteobacteria;o__Burkholderiales;f__Comamonadaceae;g__Limnohabitans;s__	0,158017	0,168947	0,033282	0,020768	0,020502	0,024671	0,704135	0,119835	0,12996	0,122611	0,094734	0,063371	0,223516	0,107708	0,076895	0,251414	1,074038	0,078691	0,617774	0,181451
k__Bacteria;p__Proteobacteria;c__Betaproteobacteria;o__Burkholderiales;f__Comamonadaceae;g__Methylibium;s__	0,066534	0,084474	0,020801	0,004154	0,0041	0,057566	0,24473	0,033058	0,116964	0,029596	0,030143	0,00845	0,248819	0,081858	0,099511	0,138278	0,09684	0,066266	0,137747	0,094849
k__Bacteria;p__Proteobacteria;c__Betaproteobacteria;o__Burkholderiales;f__Comamonadaceae;g__Paucibacter;s__	0	0,004224	0	0	0	0	0	0	0	0,008456	0,012918	0,00845	0,025304	0,030158	0	0,00419	0,008804	0	0,004174	0
k__Bacteria;p__Proteobacteria;c__Betaproteobacteria;o__Burkholderiales;f__Comamonadaceae;g__Polaromonas;s__	0,465735	0	0	0,004154	0	0	0	0	0	0	0	0	0	0	0	0,00419	0,004402	0	0	0
k__Bacteria;p__Proteobacteria;c__Betaproteobacteria;o__Burkholderiales;f__Comamonadaceae;g__Ramlibacter;s__	0,054059	0,101368	0,32866	0,07061	0,053305	0,131579	0,304839	0,057851	0,242592	0,266362	0,12057	0,152091	0,134953	0,073241	0,081418	0,104756	0,250902	0,066266	0,166966	0,070106
k__Bacteria;p__Proteobacteria;c__Betaproteobacteria;o__Burkholderiales;f__Comamonadaceae;g__Roseateles;s__depolymerans	0	0	0	0	0	0	0,004294	0	0,030324	0,004228	0,004306	0,004225	0,012652	0	0,01357	0	0,008804	0	0	0
k__Bacteria;p__Proteobacteria;c__Betaproteobacteria;o__Burkholderiales;f__Comamonadaceae;g__Rubrivivax;s__	0	0	0	0,004154	0,012301	0	0,08587	0	0	0,008456	0	0	0	0,008617	0,004523	0,012571	0	0	0	0,004124
k__Bacteria;p__Proteobacteria;c__Betaproteobacteria;o__Burkholderiales;f__Comamonadaceae;g__Variovorax;s__	0,004158	0,004224	0,00416	0	0	0	0,021468	0	0,008664	0	0	0,004225	0,021086	0,004308	0,004523	0,00419	0	0	0,004174	0
k__Bacteria;p__Proteobacteria;c__Betaproteobacteria;o__Burkholderiales;f__Oxalobacteraceae;Other;Other	0	0,004224	0	0	0	0	0,012881	0	0,012996	0,008456	0,051673	0,038023	0,071694	0,073241	0,027139	0,020951	0,162866	0	0,004174	0
k__Bacteria;p__Proteobacteria;c__Betaproteobacteria;o__Burkholderiales;f__Oxalobacteraceae;g__;s__	1,621756	0,810948	1,256396	0,265825	0,22142	0,682566	3,477738	0,946281	1,888754	3,745983	4,517074	2,43346	5,317982	6,01439	1,831916	6,687618	8,077296	1,992131	8,602914	4,767207
k__Bacteria;p__Proteobacteria;c__Betaproteobacteria;o__Burkholderiales;f__Oxalobacteraceae;g__Janthinobacterium;Other	0,004158	0	0	0	0	0	0	0,008264	0	0	0	0,004225	0	0,017233	0	0	0	0	0,133573	0,020619
k__Bacteria;p__Proteobacteria;c__Betaproteobacteria;o__Burkholderiales;f__Oxalobacteraceae;g__Janthinobacterium;s__	0	0	0	0	0	0	0	0	0	0	0	0	0,004217	0	0	0	0	0	0,004174	0,008248
k__Bacteria;p__Proteobacteria;c__Betaproteobacteria;o__Burkholderiales;f__Oxalobacteraceae;g__Oxalobacter;s__	0	0	0	0	0	0	0,004294	0,004132	0,004332	0,004228	0,004306	0	0	0	0	0,00419	0,004402	0	0	0
k__Bacteria;p__Proteobacteria;c__Betaproteobacteria;o__Burkholderiales;f__Oxalobacteraceae;g__Oxalobacter;s__formigenes	0	0	0	0	0	0	0	0	0	0	0,004306	0	0	0,004308	0	0	0	0	0	0
k__Bacteria;p__Proteobacteria;c__Betaproteobacteria;o__Burkholderiales;f__Oxalobacteraceae;g__Polynucleobacter;s__	0	0	0	0	0,0041	0	0	0	0	0	0	0	0	0,004308	0	0	0	0	0,004174	0,024743
k__Bacteria;p__Proteobacteria;c__Betaproteobacteria;o__Burkholderiales;f__Oxalobacteraceae;g__Ralstonia;s__	0	0	0	0	0	0,004112	0,004294	0,020661	0	0	0	0	0,004217	0	0	0	0	0,004142	0,012522	0,004124
k__Bacteria;p__Proteobacteria;c__Betaproteobacteria;o__Hydrogenophilales;f__Hydrogenophilaceae;g__Thiobacillus;s__	0,004158	0	0	0	0	0,004112	0,012881	0	0,069312	0,02114	0	0,004225	0	0	0	0	0	0	0	0

k__Bacteria;p__Proteobacteria;c__Betaproteobacteria;o__MND1;f__;g__;s__	0,652861	0,350566	0,611557	0,60226	0,992291	1,681743	0,442231	0,619835	0,30324	0,600372	0,31865	0,371779	0,122301	0,163715	0,212593	0,1718	0,325733	0,600538	0,116876	0,160831
k__Bacteria;p__Proteobacteria;c__Betaproteobacteria;o__Methylophilales;f__Methylophilaceae;g__;s__	0	0	0	0	0	0	0	0	0	0	0	0	0,008435	0	0	0	0	0	0	0,012372
k__Bacteria;p__Proteobacteria;c__Betaproteobacteria;o__Neisseriales;f__Neisseriaceae;Other;Other	0	0	0	0	0	0	0,004294	0,008264	0	0	0,004306	0	0	0	0,004523	0,00838	0,004402	0	0,016697	0
k__Bacteria;p__Proteobacteria;c__Betaproteobacteria;o__Neisseriales;f__Neisseriaceae;g__;s__	0	0	0	0	0	0	0	0	0	0	0	0	0	0	0	0	0	0	0	0,008248
k__Bacteria;p__Proteobacteria;c__Betaproteobacteria;o__Procabacteriales;f__Procabacteriaceae;g__;s__	0	0	0	0	0	0	0	0	0	0,008456	0	0	0	0	0	0	0	0	0	0
k__Bacteria;p__Proteobacteria;c__Betaproteobacteria;o__Rhodocyclales;f__Rhodocyclaceae;Other;Other	0,020792	0	0	0,008307	0,0041	0	0,008587	0	0,008664	0,012684	0,012918	0	0,021086	0,02585	0,004523	0,00419	0,030813	0	0,012522	0,008248
k__Bacteria;p__Proteobacteria;c__Betaproteobacteria;o__Rhodocyclales;f__Rhodocyclaceae;g__;s__	0,008317	0,008447	0,012481	0	0	0	0,103044	0,008264	0,090972	0,190259	0,167937	0,105619	0,274123	0,232648	0,045232	0,146658	0,290519	0,012425	0,116876	0,049487
k__Bacteria;p__Proteobacteria;c__Betaproteobacteria;o__Rhodocyclales;f__Rhodocyclaceae;g__Hydrogenophilus;s__	0,008317	0	0	0	0	0	0	0	0,017328	0	0	0	0	0	0,004523	0	0	0	0	0
k__Bacteria;p__Proteobacteria;c__Betaproteobacteria;o__Rhodocyclales;f__Rhodocyclaceae;g__KD1-23;s__	0	0	0	0	0,0041	0	0,012881	0	0,004332	0	0,017224	0	0,008435	0,030158	0	0,00419	0,008804	0,004142	0,016697	0
k__Bacteria;p__Proteobacteria;c__Betaproteobacteria;o__Rhodocyclales;f__Rhodocyclaceae;g__Thauera;s__	0	0	0	0	0	0	0	0	0,004332	0	0	0,004225	0	0	0	0	0	0	0	0
k__Bacteria;p__Proteobacteria;c__Betaproteobacteria;o__SBla14;f__;g__;s__	0	0	0	0	0	0	0	0	0	0	0,004306	0	0	0	0	0	0	0	0,004174	0
k__Bacteria;p__Proteobacteria;c__Betaproteobacteria;o__SC-I-84;f__;g__;s__	0	0	0	0	0	0	0	0	0	0	0	0	0	0	0	0	0	0	0,008348	0
k__Bacteria;p__Proteobacteria;c__Deltaproteobacteria;o__;f__;g__;s__	0	0	0,054083	0,008307	0	0,004112	0	0	0	0	0	0,004225	0	0	0	0	0	0,008283	0	0,004124
k__Bacteria;p__Proteobacteria;c__Deltaproteobacteria;o__Bdellovibrionales;f__Bacteriovoracaceae;g__;s__	0,016633	0,008447	0	0	0	0,004112	0,012881	0,028926	0,038988	0,012684	0,004306	0,00845	0,008435	0,012925	0,01357	0,00419	0,026411	0,033133	0	0,008248
k__Bacteria;p__Proteobacteria;c__Deltaproteobacteria;o__Bdellovibrionales;f__Bdellovibrionaceae;g__Bdellovibrio;s__	0,008317	0,008447	0,008321	0,15368	0	0,004112	0,004294	0,008264	0,008664	0,012684	0	0,00845	0,004217	0,008617	0,004523	0	0,013205	0	0,008348	0
k__Bacteria;p__Proteobacteria;c__Deltaproteobacteria;o__FAC87;f__;g__;s__	0	0	0	0	0	0	0	0	0,004332	0	0,012918	0	0	0	0	0	0	0	0	0
k__Bacteria;p__Proteobacteria;c__Deltaproteobacteria;o__MIZ46;f__;g__;s__	0,012475	0,012671	0,16225	0,128759	0,0041	0,008224	0	0	0,012996	0	0	0	0	0	0,009046	0,00838	0,004402	0,028992	0	0,004124
k__Bacteria;p__Proteobacteria;c__Deltaproteobacteria;o__Myxococcales;f__;g__;s__	2,20392	1,67258	1,09414	0,64379	0,56175	0,76069	2,8423	1,27272	2,57754	3,33587	2,72574	2,69539	2,79605	2,52466	1,86810	2,56442	2,02482	3,37129	0,73047	1,03509

	5		7	5	2	1		7	3		6	5	3	5	2	5	6	8	5	4
k__Bacteria;p__Proteobacteria;c__Deltaproteobacteria;o__Myxococcales;f__0319-6G20;g__;s__	0	0,012671	0	0	0	0	0	0	0	0	0	0,004225	0	0	0,004523	0,00419	0	0,008283	0	0
k__Bacteria;p__Proteobacteria;c__Deltaproteobacteria;o__Myxococcales;f__Haliangiaceae;g__;s__	0,241184	0,122487	0,9943	0,228443	3,977366	3,527961	0,317719	0,140496	0,15162	0,803315	0,611463	1,681453	0,04639	0,02585	0,298534	0,636916	0,083634	0,219507	0,004174	0,008248
k__Bacteria;p__Proteobacteria;c__Deltaproteobacteria;o__Myxococcales;f__Myxococcaceae;g__Myxococcus;s__	0	0,004224	0	0	0	0	0,004294	0	0,004332	0	0	0	0	0	0,004523	0,012571	0,008804	0,033133	0,004174	0,004124
k__Bacteria;p__Proteobacteria;c__Deltaproteobacteria;o__Myxococcales;f__OM27;g__;s__	0,004158	0,008447	0,320339	0,024921	0,098409	0,106908	0,004294	0	0,008664	0,004228	0	0,004225	0	0	0	0,00419	0	0,004142	0	0
k__Bacteria;p__Proteobacteria;c__Deltaproteobacteria;o__PB19;f__;g__;s__	0,016633	0,118263	2,95378	0,647948	0,270625	0,291941	0	0	0	0,016912	0,004306	0,00845	0	0	0	0,012571	0	0,008283	0	0
k__Bacteria;p__Proteobacteria;c__Deltaproteobacteria;o__Spirobacillales;f__;g__;s__	0,029108	0,008447	0	0,004154	0	0,024671	0,030055	0	0,02166	0,059192	0,004306	0,00845	0,004217	0	0	0	0	0,004142	0,012522	0
k__Bacteria;p__Proteobacteria;c__Deltaproteobacteria;o__Syntrophobacterales;f__Syntrophobacteraceae;g__;s__	0,0499	0,033789	0,016641	0,029075	0,028703	0,012336	0,038642	0,041322	0,038988	0,067648	0,047367	0,063371	0,054825	0,021542	0,058802	0,050283	0,12325	0,144958	0,008348	0,037115
k__Bacteria;p__Proteobacteria;c__Epsilonproteobacteria;o__Campylobacterales;f__Campylobacteraceae;g__Sulfurospirillum;s__	0	0	0	0	0	0	0,004294	0	0	0	0	0	0	0	0	0	0	0	0	0,004124
k__Bacteria;p__Proteobacteria;c__Epsilonproteobacteria;o__Campylobacterales;f__Helicobacteraceae;g__Helicobacter;s__pylori	0	0,012671	0	0	0	0	0	0,239669	0	0,008456	0	0,00845	0	0,004308	0,009046	0,00419	0	0,004142	0,066786	0
k__Bacteria;p__Proteobacteria;c__Gammaproteobacteria;Other;Other;Other;Other	0	0	0	0	0	0	0	0	0,004332	0	0	0	0	0,004308	0	0	0	0	0	0
k__Bacteria;p__Proteobacteria;c__Gammaproteobacteria;o__Aeromonadales;f__Aeromonadaceae;g__;s__	0	0	0	0	0	0	0	0	0	0,008456	0	0	0	0	0	0	0	0	0	0,012372
k__Bacteria;p__Proteobacteria;c__Gammaproteobacteria;o__Alteromonadales;f__211ds20;g__;s__	0,174651	0,050684	0,00416	0,004154	0,008201	0,016447	0,206088	0,028926	0,069312	0,587688	0,02153	0,097169	0,105432	0,068933	0,036186	0,142468	0,105643	0,033133	0,346454	0,313415
k__Bacteria;p__Proteobacteria;c__Gammaproteobacteria;o__Alteromonadales;f__Alteromonadaceae;g__Cellvibrio;s__	0,004158	0	0,008321	0	0	0	0	0	0	0	0	0	0	0	0,004523	0,00419	0	0	0	0
k__Bacteria;p__Proteobacteria;c__Gammaproteobacteria;o__Alteromonadales;f__[Chromatiaceae];g__Rheinheimera;s__	0	0	0	0	0	0	0	0	0	0	0,004306	0	0	0	0	0,00419	0	0	0	0
k__Bacteria;p__Proteobacteria;c__Gammaproteobacteria;o__Chromatiales;f__;g__;s__	0,145542	0,084474	0,012481	0	0,020502	0,008224	0,090164	0,024793	0,069312	0,038052	0,073203	0,054922	0,050607	0,064625	0,131174	0,062854	0,154063	0,236074	0,175314	0,041239
k__Bacteria;p__Proteobacteria;c__Gammaproteobacteria;o__Enterobacteriales;f__Enterobacteriaceae;Other;Other	0,029108	0,021118	0,00416	0	0	0,008224	0,012881	0,016529	0,047652	0,02114	0,038755	0,025349	0,04639	0,103399	0,040709	0,041902	0,022009	0,004142	0,008348	0,024743
k__Bacteria;p__Proteobacteria;c__Gammaproteobacteria;o__Enterobacteriales;f__Enterobacteriaceae;g__;s__	5,168829	2,377935	0,312019	0,394584	0,233722	1,76398	2,030827	32,33471	3,508924	4,443599	6,420359	4,803549	5,963225	12,45099	3,387914	5,891473	1,897174	7,740733	4,412072	11,74894

k__Bacteria;p__Proteobacteria;c__Gammaproteobacteria;o__Enterobacteriales;f__Enterobacteriaceae;g__Escherichia;s__coli	0	0	0	0	0	0	0	0,008264	0	0	0	0	0	0	0	0	0	0	0	0
k__Bacteria;p__Proteobacteria;c__Gammaproteobacteria;o__Enterobacteriales;f__Enterobacteriaceae;g__Plesiomonas;s__	0	0	0	0	0	0	0,004294	0	0	0	0	0	0	0	0,004523	0	0	0	0	0
k__Bacteria;p__Proteobacteria;c__Gammaproteobacteria;o__Enterobacteriales;f__Enterobacteriaceae;g__Providencia;Other	0	0	0	0	0	0	0	0,008264	0	0	0	0	0	0	0	0	0	0	0	0
k__Bacteria;p__Proteobacteria;c__Gammaproteobacteria;o__Enterobacteriales;f__Enterobacteriaceae;g__Serratia;Other	0	0,004224	0	0	0	0	0	0	0	0	0	0	0,004217	0	0	0,00419	0,008804	0	0	0
k__Bacteria;p__Proteobacteria;c__Gammaproteobacteria;o__Enterobacteriales;f__Enterobacteriaceae;g__Serratia;s__marcescens	0,054059	0,046461	0,008321	0,004154	0,0041	0,008224	0,042935	0,024793	0,125628	0,084559	0,133488	0,122518	0,164474	0,314506	0,144744	0,075424	0,079232	0,053841	0,029219	0,020619
k__Bacteria;p__Proteobacteria;c__Gammaproteobacteria;o__Enterobacteriales;f__Enterobacteriaceae;g__Trabulsiella;Other	0	0	0	0	0	0	0	0,004132	0	0	0	0,004225	0	0	0	0,00419	0	0	0,008348	0
k__Bacteria;p__Proteobacteria;c__Gammaproteobacteria;o__Legionellales;Other;Other;Other	0	0	0,00416	0	0	0	0	0	0	0,008456	0,008612	0	0	0	0	0	0	0	0	0
k__Bacteria;p__Proteobacteria;c__Gammaproteobacteria;o__Legionellales;f__Coxiellaceae;g__;s__	0,004158	0	0	0	0	0	0,008587	0	0	0	0	0	0	0	0	0	0	0	0	0
k__Bacteria;p__Proteobacteria;c__Gammaproteobacteria;o__Legionellales;f__Coxiellaceae;g__Rickettsiella;s__	0,004158	0	0	0	0	0	0	0,004132	0	0	0	0	0	0,004308	0	0	0	0,012425	0	0
k__Bacteria;p__Proteobacteria;c__Gammaproteobacteria;o__Legionellales;f__Legionellaceae;g__Legionella;s__	0	0	1,335441	0,46104	0	0	0,07299	0	0	0	0	0	0	0	0	0,00838	0	0	0	0
k__Bacteria;p__Proteobacteria;c__Gammaproteobacteria;o__Pasteurellales;f__Pasteurellaceae;g__Haemophilus;Other	0	0	0	0	0	0	0	0	0	0,008456	0	0	0	0	0	0	0	0,008283	0	0,012372
k__Bacteria;p__Proteobacteria;c__Gammaproteobacteria;o__Pseudomonadales;Other;Other;Other	0,008317	0,008447	0	0	0,008201	0,049342	0	0,004132	0,004332	0,008456	0,008612	0,00845	0	0,02585	0,022616	0,00838	0,004402	0,004142	0	0,008248
k__Bacteria;p__Proteobacteria;c__Gammaproteobacteria;o__Pseudomonadales;f__Moraxellaceae;g__Acinetobacter;s__	0,045742	0	0	0,170294	0,127112	0,550987	0,038642	0,38843	0,012996	0,093015	0,008612	0,00845	0,004217	0,008617	0,009046	0,163419	0,013205	0,157382	0,179488	0,334034
k__Bacteria;p__Proteobacteria;c__Gammaproteobacteria;o__Pseudomonadales;f__Moraxellaceae;g__Acinetobacter;s__rhizosphaerae	0	0	0	0	0	0	0	0	0	0	0	0	0	0	0	0	0	0,004142	0,033393	0,004124
k__Bacteria;p__Proteobacteria;c__Gammaproteobacteria;o__Pseudomonadales;f__Moraxellaceae;g__Enhydrobacter;s__	0	0	0	0,004154	0,008201	0,004112	0,017174	0,049587	0	0,004228	0,004306	0	0	0	0	0,012571	0	0	0,033393	0,094849
k__Bacteria;p__Proteobacteria;c__Gammaproteobacteria;o__Pseudomonadales;f__Moraxellaceae;g__Psychrobacter;s__	0	0	0	0	0	0	0	0	0	0	0	0	0	0	0	0	0	0	0,012522	0
k__Bacteria;p__Proteobacteria;c__Gammaproteobacteria;o__Pseudomonadales;f__Pseudomonadaceae;Other;Other	0	0	0	0	0	0,008224	0,004294	0	0,004332	0,004228	0,008612	0	0,004217	0,004308	0	0,00419	0	0,004142	0,004174	0,008248
k__Bacteria;p__Proteobacteria;c__Gammaproteobacteria;o__Pseudomonadales;f__Pseudomonadaceae;g__;s__	0,07069	0	0	0	0,00820	4,35855	0,02146	0,01239	0,01299	0,01691	0,01291	0	0,01686	0,01292	0,00904	0,00838	0,00880	0,00828	0,01252	0,01237

	2				1	3	8	7	6	2	8		9	5	6		4	3	2	2
k__Bacteria;p__Proteobacteria;c__Gammaproteobacteria;o__Pseudomonadales;f__Pseudomonadaceae;g__Pseudomonas;s__	0,153859	0,021118	0	0,012461	0,036903	0,102796	0,055816	0,061983	0,082308	0,059192	0,025836	0,004225	0,054825	0,02585	0,045232	0,054473	0,074831	0,066266	0,100179	0,177327
k__Bacteria;p__Proteobacteria;c__Gammaproteobacteria;o__Pseudomonadales;f__Pseudomonadaceae;g__Pseudomonas;s__fragi	0	0	0	0	0	0,004112	0	0	0	0	0	0	0	0	0	0	0	0,004142	0	0
k__Bacteria;p__Proteobacteria;c__Gammaproteobacteria;o__Pseudomonadales;f__Pseudomonadaceae;g__Pseudomonas;s__stutzeri	0	0	0	0	0	0,004112	0	0	0	0,004228	0	0	0	0	0	0	0	0	0,004174	0
k__Bacteria;p__Proteobacteria;c__Gammaproteobacteria;o__Pseudomonadales;f__Pseudomonadaceae;g__Pseudomonas;s__viridiflava	0	0	0	0	0	0	0,012881	0	0	0	0,004306	0,00845	0,008435	0,004308	0	0	0	0	0	0
k__Bacteria;p__Proteobacteria;c__Gammaproteobacteria;o__Xanthomonadales;f__Sinobacteraceae;g__;s__	0,004158	0	0	0	0	0	0	0	0,008664	0	0	0	0	0	0,004523	0	0	0	0,008348	0
k__Bacteria;p__Proteobacteria;c__Gammaproteobacteria;o__Xanthomonadales;f__Sinobacteraceae;g__Steroidobacter;s__	0	0	0	0	0	0	0	0	0	0	0	0,016899	0	0,004308	0	0	0,004402	0	0	0
k__Bacteria;p__Proteobacteria;c__Gammaproteobacteria;o__Xanthomonadales;f__Xanthomonadaceae;Other;Other	0	0,004224	0,00416	0,008307	0,008201	0,896382	0,163153	0,057851	0	0,224083	0,783706	0,097169	0,193995	0,034466	0,094988	0,842238	0,325733	0,323048	0,705431	2,123799
k__Bacteria;p__Proteobacteria;c__Gammaproteobacteria;o__Xanthomonadales;f__Xanthomonadaceae;g__;s__	0,170492	0,063355	0,16641	0,045689	0,069706	0,131579	0,145979	0,136364	0,246924	0,219855	0,176549	0,198564	0,134953	0,224032	0,144744	0,129897	0,193679	0,289915	0,175314	0,07423
k__Bacteria;p__Proteobacteria;c__Gammaproteobacteria;o__Xanthomonadales;f__Xanthomonadaceae;g__Luteibacter;s__rhizovicinus	0	0	0	0	0	0	0	0	0	0	0	0	0,012652	0,004308	0	0	0	0	0	0,004124
k__Bacteria;p__Proteobacteria;c__Gammaproteobacteria;o__Xanthomonadales;f__Xanthomonadaceae;g__Luteimonas;s__	0	0	0,008321	0,004154	0,008201	0,086349	0,128805	0	0,012996	0,097243	0	0,012674	0	0	0,004523	0	0	0	0	0
k__Bacteria;p__Proteobacteria;c__Gammaproteobacteria;o__Xanthomonadales;f__Xanthomonadaceae;g__Lysobacter;s__	0,611277	0,527961	0,312019	0,103838	0,123011	0,324836	1,288051	0,400826	0,745105	0,866734	0,542566	0,688635	1,25253	1,339882	0,493034	0,540541	1,091645	1,010561	3,431147	0,672193
k__Bacteria;p__Proteobacteria;c__Gammaproteobacteria;o__Xanthomonadales;f__Xanthomonadaceae;g__Pseudoxanthomonas;s__mexicana	0	0	0	0	0,0041	0,004112	0	0	0	0	0	0	0	0	0	0,00419	0	0	0	0,004124
k__Bacteria;p__Proteobacteria;c__Gammaproteobacteria;o__Xanthomonadales;f__Xanthomonadaceae;g__Stenotrophomonas;Other	0	0	0	0	0	0	0	0,004132	0	0	0	0	0	0	0	0	0	0,016567	0	0,004124
k__Bacteria;p__Proteobacteria;c__Gammaproteobacteria;o__Xanthomonadales;f__Xanthomonadaceae;g__Thermomonas;Other	0	0	0	0	0	0	0	0	0	0,012684	0,025836	0	0	0	0	0,00838	0,052822	0,004142	0,004174	0,004124
k__Bacteria;p__Proteobacteria;c__Gammaproteobacteria;o__Xanthomonadales;f__Xanthomonadaceae;g__Thermomonas;s__	0	0	0	0	0	0	0	0	0	0	0	0	0,004217	0	0	0	0	0	0	0,004124
k__Bacteria;p__Proteobacteria;c__Gammaproteobacteria;o__Xanthomonadales;f__Xanthomonadaceae;g__Thermomonas;s__fusca	0	0	0	0	0	0	0,004294	0	0	0	0,004306	0	0,004217	0	0	0	0,013205	0	0	0
k__Bacteria;p__Proteobacteria;c__Gammaproteobacteria;o__Xanthomonadales;f__Xanthomonadaceae;g__Xanthomonas;s__	0	0	0	0	0	0	0	0	0	0	0	0	0	0	0	0	0	0	0,008348	0

k__Bacteria;p__Proteobacteria;c__TA18;o__CV90;f__;g__;s__	0	0	0	0	0	0,004112	0,004294	0	0,004332	0,004228	0,004306	0	0	0	0	0	0	0	0	0
k__Bacteria;p__Proteobacteria;c__TA18;o__PHOS-HD29;f__;g__;s__	0,041583	0,021118	0,091526	0,066456	0,012301	0,004112	0,07299	0,045455	0,017328	0,02114	0	0,004225	0,012652	0,012925	0,022616	0,016761	0,008804	0,033133	0	0
k__Bacteria;p__Spirochaetes;c__[Leptospirae];o__[Leptospirales];f__Leptospiraceae;g__Turneriella;s__	0	0	0	0	0	0	0	0	0	0	0,004306	0	0,008435	0,004308	0	0,00419	0,004402	0,004142	0,012522	0
k__Bacteria;p__TM7;c__MJK10;o__;f__;g__;s__	0,079009	0,016895	0,024962	0,041535	0,036903	0,028783	0,034348	0	0,017328	0,004228	0,004306	0,00845	0,059042	0,034466	0	0,00419	0,057223	0,041416	0,033393	0,004124
k__Bacteria;p__TM7;c__SC3;o__;f__;g__;s__	0,070692	0,021118	0,220493	0,415351	0,155814	0,065789	0,025761	0	0,060648	0,02114	0,008612	0,054922	0,016869	0	0,058802	0,050283	0,044018	0,02485	0,016697	0,008248
k__Bacteria;p__TM7;c__TM7-1;o__;f__;g__;s__	1,230872	0,223855	0,341141	0,610567	0,369034	0,296053	0,386415	0,107438	0,355224	0,359378	0,288507	0,299958	0,286775	0,443755	0,384476	0,196941	0,51501	0,439014	0,154443	0,136088
k__Bacteria;p__TM7;c__TM7-3;Other;Other;Other;Other	0	0	0	0	0	0	0,008587	0	0	0	0	0	0	0	0	0	0	0,012425	0	0
k__Bacteria;p__TM7;c__TM7-3;o__;f__;g__;s__	0,145542	0,084474	0,108167	0,103838	0,086108	0,160362	0,115925	0,020661	0,125628	0,308642	0,163631	0,143642	0,037955	0,353281	0,085942	0,079614	0,127652	0,219507	0,367325	0,024743
k__Bacteria;p__TM7;c__TM7-3;o__EW055;f__;g__;s__	0	0	0	0	0	0	0	0	0	0	0	0	0	0	0	0	0	0	0,008348	0
k__Bacteria;p__TM7;c__TM7-3;o__I025;f__;g__;s__	0,133067	0,046461	0,037442	0,053996	0,065606	0,04523	0,077283	0,033058	0,06498	0,190259	0,116264	0,147866	0,033738	0,284348	0,045232	0,033522	0,158465	0,07455	0,07096	0,107221
k__Bacteria;p__Tenericutes;c__Mollicutes;o__RF39;f__;g__;s__	0	0	0	0	0	0	0,004294	0	0	0	0	0	0	0	0	0	0	0	0,004174	0
k__Bacteria;p__Verrucomicrobia;c__Opitutae;o__Opitutales;f__Opitutaceae;g__Opitutus;s__	0	0	0	0	0	0	0	0	0	0	0	0,021124	0	0	0	0	0	0	0	0,008248
k__Bacteria;p__Verrucomicrobia;c__Verrucomicrobiae;o__Verrucomicrobiales;f__Verrucomicrobiaceae;g__;s__	0	0	0	0	0	0	0	0	0	0	0	0	0	0	0	0	0	0	0,008348	0
k__Bacteria;p__Verrucomicrobia;c__[Pedosphaerae];o__[Pedosphaerales];f__;g__;s__	0,012475	0,008447	0	0	0	0	0,004294	0,016529	0,004332	0	0,004306	0,004225	0,008435	0,008617	0,018093	0,00838	0,017607	0,020708	0,004174	0
k__Bacteria;p__Verrucomicrobia;c__[Spartobacteria];o__[Chthoniobacterales];f__[Chthoniobacteraceae];g__;s__	0	0	0	0	0	0,008224	0,008587	0,008264	0	0	0	0	0	0	0	0	0,008804	0	0,008348	0
k__Bacteria;p__Verrucomicrobia;c__[Spartobacteria];o__[Chthoniobacterales];f__[Chthoniobacteraceae];g__DA101;s__	0,045742	0,004224	0,099846	0,029075	0,008201	0,037007	0,055816	0,045455	0,103968	0,046508	0,038755	0,042248	0,025304	0,034466	0,040709	0,029332	0,092438	0,082833	0,008348	0,012372
k__Bacteria;p__WPS-2;c__;o__;f__;g__;s__	0,178809	0,325224	0,786288	0,024921	0,049205	0,139803	0,081577	0,421488	0,19494	0,799087	1,347802	1,170258	0,075911	0,848736	0,036186	0,473497	0,506207	0,078691	0,083483	0,049487
k__Bacteria;p__WS2;c__SHA-109;o__;f__;g__;s__	0	0	0	0	0	0	0	0	0	0	0	0	0	0	0	0	0	0	0	0,00824

																				8
k__Bacteria;p__[Thermi];c__Deinococci;o__Deinococcales;f__Deinococcaceae;g__Deinococcus;s__	0,083167	0,126711	0	0,020768	0,036903	0,028783	0,038642	0,041322	0,1083	0,06342	0,034449	0,063371	0,147605	0,116324	0,049756	0,17599	0,074831	0,132533	0,258797	0,131964
k__Bacteria;p__[Thermi];c__Deinococci;o__Deinococcales;f__Deinococcaceae;g__R18-435;s__	0,020792	0,059132	0,062404	0,016614	0,061506	0,012336	0,051522	0,020661	0,008664	0,004228	0,008612	0,016899	0,008435	0,008617	0,004523	0,00838	0,008804	0,004142	0	0,016496
k__Bacteria;p__[Thermi];c__Deinococci;o__Deinococcales;f__Trueperaceae;g__Truepera;s__	0,041583	0,038013	0,016641	0,15368	0,073807	0,049342	0,094457	0,016529	0,082308	0,038052	0,047367	0,033798	0,075911	0,030158	0,076895	0,050283	0,066027	0,0497	0,029219	0,037115
k__Bacteria;p__[Thermi];c__Deinococci;o__Thermales;f__Thermaceae;g__Meiothermus;s__	0	0	0	0	0	0	0	0	0,004332	0	0	0	0,004217	0	0	0	0	0,020708	0	0
