# Supplementary material for: Microbial diversity and mineral composition of weathered serpentine rock of the Khalilovsky massif
Source: PLoS One. 2019 Dec 12;14(12):e0225929. doi: 10.1371/journal.pone.0225929 (PMC6907791; doi:10.1371/journal.pone.0225929)
Supplement: S4 Table — (PDF) [file pone.0225929.s010.pdf]

Table S4. Spearman's rank correlation coefficients between relative abundance of OTUs and depth of serpentine minerals.

Taxonomy	Spearman's rank correlation coefficient	p-value*
k__*Bacteria*; p__*Acidobacteria*; c__*Acidobacteria*-6; o__iii1-15; f__; g__; s__	-0.7698168	0.0138233
k__*Bacteria*; p__*Actinobacteria*; c__*Acidimicrobiia*; o__*Acidimicrobiales*; f__; g__; s__	-0.81075183	0.00425812
k__*Bacteria*; p__*Actinobacteria*; c__*Acidimicrobiia*; o__*Acidimicrobiales*; f__; g__; s__	-0.77387235	0.01349225
k__*Bacteria*; p__*Actinobacteria*; c__*Acidimicrobiia*; o__*Acidimicrobiales*; f__; g__; s__	-0.7698168	0.0138233
k__*Bacteria*; p__*Actinobacteria*; c__*Acidimicrobiia*; o__*Acidimicrobiales*; f__; g__; s__	-0.76385923	0.0138233
k__*Bacteria*; p__*Actinobacteria*; c__*Actinobacteria*; o__*Actinomycetales*; f__*Actinosynnemataceae*; g__; s__	**0.73764792**	0.02773051
k__*Bacteria*; p__*Actinobacteria*; c__*Actinobacteria*; o__*Actinomycetales*; f__*Nocardioidaceae*; g__; s__	-0.7757131	0.0131689
k__*Bacteria*; p__*Actinobacteria*; c__*Actinobacteria*; o__*Actinomycetales*; f__*Pseudonocardiaceae*; g__*Amycolatopsis*; s__	**0.76431618**	0.0138233
k__*Bacteria*; p__*Actinobacteria*; c__*Actinobacteria*; o__*Actinomycetales*; f__*Streptomycetaceae*; g__*Streptomyces*; s__	**0.73537608**	0.02932494
k__*Bacteria*; p__*Actinobacteria*; c__*Actinobacteria*; o__*Actinomycetales*; f__*Streptomycetaceae*; g__*Streptomyces*; s__	**0.80667195**	0.00478713
k__*Bacteria*; p__*Actinobacteria*; c__MB-A2-108; o__0319-7L14; f__; g__; s__	**0.72063347**	0.04441844
k__*Bacteria*; p__*Actinobacteria*; c__*Thermoleophilia*; o__*Gaiellales*; f__*Gaiellaceae*; g__; s__	-0.78609424	0.00854366
k__*Bacteria*; p__*Actinobacteria*; c__*Thermoleophilia*; o__*Gaiellales*; f__*Gaiellaceae*; g__; s__	-0.74966213	0.02017219
k__*Bacteria*; p__*Actinobacteria*; c__*Thermoleophilia*; o__*Gaiellales*; f__*Gaiellaceae*; g__; s__	-0.720236	0.04441844
k__*Bacteria*; p__*Actinobacteria*; c__*Thermoleophilia*; o__*Gaiellales*; f__*Gaiellaceae*; g__; s__	**0.75973718**	0.01472673
k__*Bacteria*; p__*Actinobacteria*; c__*Thermoleophilia*; o__*Solirubrobacterales*; f__; g__; s__	-0.71579186	0.04931098
k__*Bacteria*; p__*Actinobacteria*; c__*Thermoleophilia*; o__*Solirubrobacterales*; f__*Conexibacteraceae*; g__; s__	**0.82413459**	0.00299772

k__*Bacteria*; p__*Actinobacteria*; c__*Thermoleophilia*; o__*Solirubrobacterales*; f__*Solirubrobacteraceae*; g__; s__	**0.75985048**	0.01472673
k__*Bacteria*; p__*Chloroflexi*; c__Ellin6529; o__; f__; g__; s__	-0.72190739	0.04399233
k__*Bacteria*; p__*Chloroflexi*; c__P2-11E; o__; f__; g__; s__	**0.73471789**	0.02932494
k__*Bacteria*; p__*Chloroflexi*; c__P2-11E; o__; f__; g__; s__	**0.74567669**	0.0222152
k__*Bacteria*; p__*Chloroflexi*; c__P2-11E; o__; f__; g__; s__	**0.75027611**	0.02017219
k__*Bacteria*; p__*Chloroflexi*; c__TK10; o__B07_WMSP1; f__; g__; s__	-0.79882273	0.0055392
k__*Bacteria*; p__GAL15; c__; o__; f__; g__; s__	**0.7561646**	0.01652607
k__*Bacteria*; p__GAL15; c__; o__; f__; g__; s__	**0.75985048**	0.01472673
k__*Bacteria*; p__*Gemmatimonadetes*; c__*Gemmatimonadetes*; o__; f__; g__; s__	-0.83956694	0.00130184
k__*Bacteria*; p__*Planctomycetes*; c__*Phycisphaerae*; o__WD2101; f__; g__; s__	-0.88922654	5.27E-05
k__*Bacteria*; p__*Planctomycetes*; c__*Phycisphaerae*; o__WD2101; f__; g__; s__	-0.81089351	0.00425812
k__*Bacteria*; p__*Proteobacteria*; c__; o__; f__; g__; s__	-0.76722679	0.0138233
k__*Bacteria*; p__*Proteobacteria*; c__*Alphaproteobacteria*; o__*Rhizobiales*; f__*Methylobacteriaceae*; g__*Methylobacterium*; s__	**0.84975305**	0.00119961
k__*Bacteria*; p__*Proteobacteria*; c__*Betaproteobacteria*; o__; f__; g__; s__	**0.80045081**	0.0055392
k__*Bacteria*; p__*Proteobacteria*; c__*Betaproteobacteria*; o__*Burkholderiales*; f__*Comamonadaceae*	**0.74372798**	0.0232746
k__*Bacteria*; p__*Proteobacteria*; c__*Betaproteobacteria*; o__*Burkholderiales*; f__*Comamonadaceae*; g__; s__	**0.74102212**	0.0250986
k__*Bacteria*; p__*Proteobacteria*; c__*Betaproteobacteria*; o__*Burkholderiales*; f__*Comamonadaceae*; g__; s__	**0.74639294**	0.0222152
k__*Bacteria*; p__*Proteobacteria*; c__*Betaproteobacteria*; o__*Burkholderiales*; f__*Comamonadaceae*; g__*Delftia*; s__	**0.76377902**	0.0138233
k__*Bacteria*; p__*Proteobacteria*; c__*Betaproteobacteria*; o__*Burkholderiales*; f__*Oxalobacteraceae*; g__; s__	**0.71636767**	0.04931098
k__*Bacteria*; p__*Proteobacteria*; c__*Betaproteobacteria*; o__*Burkholderiales*; f__*Oxalobacteraceae*; g__; s__	**0.76464189**	0.0138233
k__*Bacteria*; p__*Proteobacteria*; c__*Betaproteobacteria*; o__*Burkholderiales*; f__*Oxalobacteraceae*; g__; s__	**0.78802707**	0.00831419

k__*Bacteria*; p__*Proteobacteria*; c__*Betaproteobacteria*; o__*Burkholderiales*; f__*Oxalobacteraceae*; g__; s__	**0.84139357**	0.00130184
k__*Bacteria*; p__*Proteobacteria*; c__*Betaproteobacteria*; o__MND1; f__; g__; s__	-0.81784035	0.00371264
k__*Bacteria*; p__*Proteobacteria*; c__*Betaproteobacteria*; o__MND1; f__; g__; s__	-0.76848635	0.0138233
Unassigned	-0.7990866	0.0055392
Unassigned	-0.79150895	0.00750589
Unassigned	-0.76889889	0.0138233
Unassigned	-0.72782432	0.03667223

* p-value corrected by the Benjamini-Hochberg FDR procedure for multiple comparisons.
